# Early life high fructose exposure disrupts microglia phagocytosis and impedes neurodevelopment

**DOI:** 10.1038/s41586-025-09098-5

**Published:** 2025-06-11

**Authors:** Zhaoquan Wang, Allie Lipshutz, Celia Martínez de la Torre, Alissa J. Trzeciak, Zong-Lin Liu, Isabella C. Miranda, Tomi Lazarov, Ana C. Codo, Jesús E. Romero-Pichardo, Achuth Nair, Tanya Schild, Waleska Saitz Rojas, Pedro H. V. Saavedra, Ann Baako, Kelvin Fadojutimi, Michael S. Downey, Frederic Geissmann, Giuseppe Faraco, Li Gan, Jon Iker Etchegaray, Christopher D. Lucas, Marina Tanasova, Christopher N. Parkhurst, Melody Y. Zeng, Kayvan R. Keshari, Justin S. A. Perry

**Affiliations:** 1 Immunology Program, Sloan Kettering Institute, Memorial Sloan Kettering Cancer Center, New York, NY, USA; 2 Immunology and Microbial Pathogenesis Program, Weill Cornell Medicine, New York, NY, USA; 3 Department of Radiology, Sloan Kettering Institute, Memorial Sloan Kettering Cancer Center, New York, NY, USA; 4 Molecular Pharmacology Program, Sloan Kettering Institute, Memorial Sloan Kettering Cancer Center, New York, NY, USA; 5 Division of Pulmonary and Critical Care Medicine, Weill Cornell Medicine, New York, NY, USA; 6 Louis V. Gerstner Jr. Graduate School of Biomedical Sciences, Memorial Sloan Kettering Cancer Center, New York, NY, USA; 7 Neuroscience Graduate Program, Weill Cornell Medicine, New York, NY, USA; 8 Feil Family Brain and Mind Research Institute, Weill Cornell Medicine, New York, NY, USA; 9 Helen and Robert Appel Alzheimer’s Disease Research Institute, Feil Family Brain and Mind Research Institute, Weill Cornell Medicine, New York, NY, USA; 10 Department of Pathology and Immunology, Washington University School of Medicine, St. Louis, MO, USA; 11 University of Edinburgh Centre for Inflammation Research, Queen’s Medical Research Institute, Edinburgh BioQuarter, Edinburgh, Scotland, UK; 12 Institute for Regeneration and Repair, Edinburgh BioQuarter, Edinburgh, Scotland, UK; 13 Department of Chemistry, Michigan Technological University, Houghton, MI, USA; 14 Health Research Institute, Michigan Technological University, Houghton, MI, USA; 15 Gale and Ira Drukier Institute for Children’s Health, Weill Cornell Medicine, New York, NY, USA; 16 Department of Pediatrics, Neonatal Medicine, Weill Cornell Medicine, New York, NY, USA

## Abstract

Despite the success of fructose as a low-cost food additive, recent epidemiological evidence suggests that high fructose consumption by pregnant mothers or during adolescence is associated with disrupted neurodevelopment^1–3^. An essential step in appropriate mammalian neurodevelopment is the phagocytic elimination of newly-formed neurons by microglia, the central nervous system’s (CNS) resident professional phagocyte^4^. Whether early life high fructose consumption affects microglia phagocytosis and if this directly impacts neurodevelopment remains unknown. Here, we show that both offspring born to dams fed a high fructose diet and neonates exposed to high fructose exhibit decreased phagocytic activity *in vivo*. Importantly, deletion of the high affinity fructose transporter SLC2A5 (GLUT5) in neonatal microglia completely reversed microglia phagocytic dysfunction, suggesting that high fructose directly affects neonatal development by suppressing microglia phagocytosis. Mechanistically, we found that high fructose treatment of both mouse and human microglia suppresses phagocytosis capacity which is reversed in GLUT5-deficient microglia. Additionally, we found that high fructose drives significant GLUT5-dependent fructose uptake and catabolism into fructose 6-phosphate, rewiring microglia metabolism towards a hypo-phagocytic state in part by enforcing mitochondrial localization of the enzyme hexokinase 2. Importantly, mice exposed to high fructose as neonates develop anxiety-like behavior as adolescents which is rescued in GLUT5-deficient animals. Our findings provide a mechanistic explanation for the epidemiological observation that early life high fructose exposure is associated with increased prevalence of adolescent anxiety disorders.

High fructose corn syrup (HFCS)-containing foods and beverages have faced increasing scrutiny over the past few decades, as the health hazards of overconsumption continue to be highlighted^5^. Recently, multiple epidemiological studies have suggested that high fructose consumption by pregnant mothers or during adolescence negatively impacts neurodevelopment, including increased risk of mood and anxiety disorder development^1–3^. Furthermore, a series of independent studies found that fructose metabolism can occur in the central nervous system (CNS)^6, 7^. Whether the neurodevelopmental effects observed in epidemiological studies are a result of fructose metabolism in the CNS and how high fructose affects CNS cellular function remains unknown.

In mammals, neurodevelopment occurs through well-defined stages that are tightly temporally controlled^8^. In particular, neurons proliferate and form synapses which, if unreinforced, die by neglect via programmed cell death and must be cleared via phagocytosis^9^. The phagocytic clearance of dying neurons (termed efferocytosis) is executed by microglia, the CNS’s resident macrophage and professional phagocyte^10, 11^. Importantly, disrupted removal of dying neurons can have catastrophic consequences on neurodevelopment in animals, including the development of anxiety-like behavior^12, 13^. Here, we investigated the hypothesis that early life high fructose exposure directly affects microglia phagocytosis resulting in defective learning and the development of anxiety-like behavior.

## Early life high fructose exposure suppresses microglial phagocytosis *in vivo*

**Extended Data Fig. 1:**
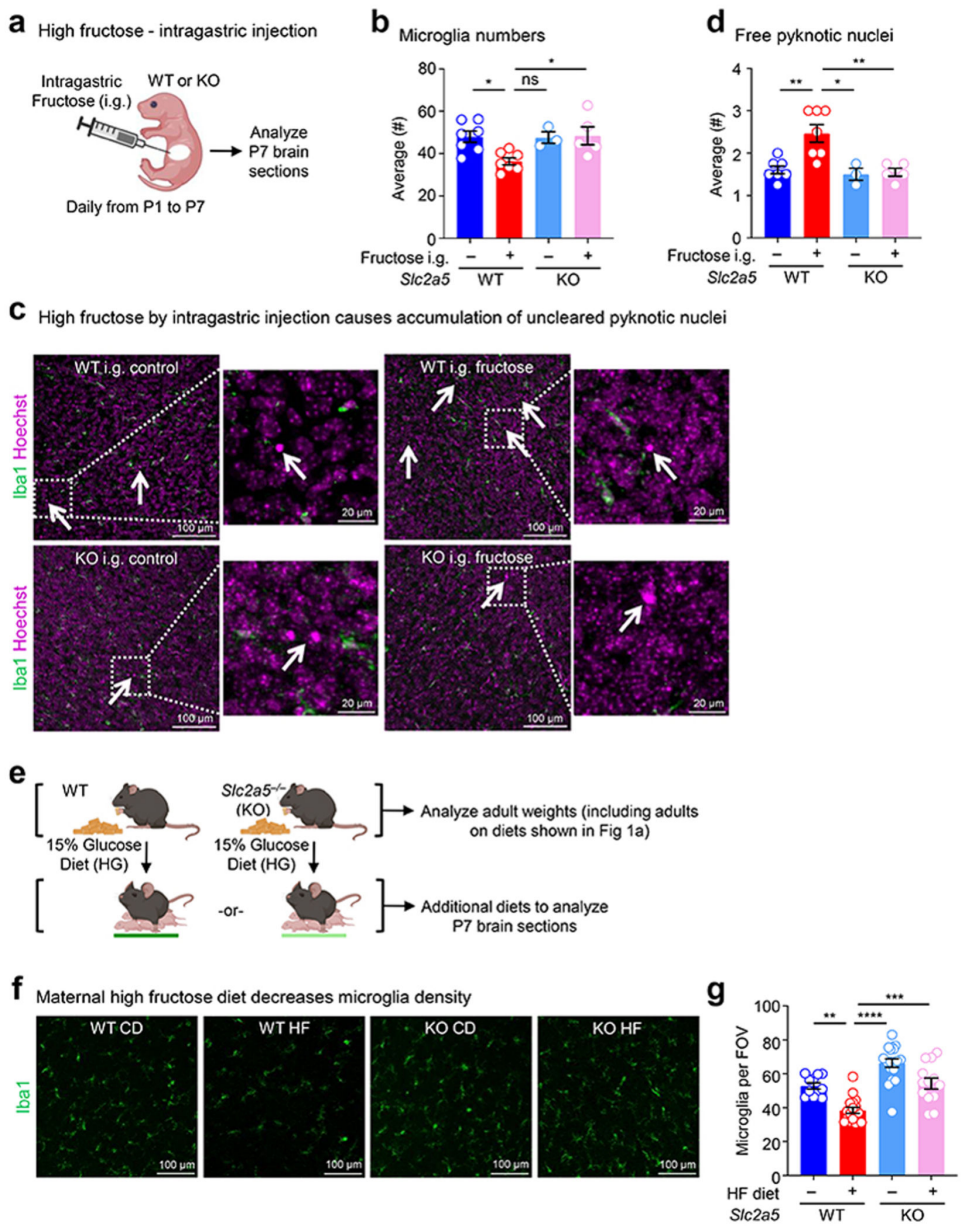
The effect of high fructose or glucose on neonatal microglia numbers and function *in vivo*. **(a-d)** Intragastric delivery of fructose to neonates leads to decreased microglia numbers and increased uncleared apoptotic cells. **(a)** Neonatal wildtype (WT) and *Slc2a5*^–/–^ (KO) mice were injected daily into the visible milk spot from P1 to P7 with fructose (45 mg per mouse) or sterile water control. Brains were then collected and analyzed by confocal microscopy (20x objective, 20 μm thick image stacks with 2 μm step-size). **(b)** Microglial cell count was quantified from four fields of view (FOVs) per mouse for each condition from **(a)** and each dot represents the mean for one mouse, with *N* = 7 for WT CD, *N* = 7 for WT HF, *N* = 3 for KO CD, and *N* = 5 for KO HF. Data are shown as mean ± SEM. Significance was determined by two-way ANOVA with Tukey’s multiple comparisons test. ns = not significant, *p < .05. Representative images **(c)** and **(d)** quantitation of uncleared (free) apoptotic cells (pyknotic nuclei) from **(a)**, determined using Iba1 staining of microglia and Hoechst staining of nuclei. Arrows denote condensed chromatin (pyknotic nuclei) which is a hallmark indicator of apoptosis. Scale bars, 100 µm (inlay, 20 µm). Quantitation of free pyknotic nuclei in **(c)** is based on four FOVs per mouse and each dot represents the mean for one mouse, with *N* = 7 for WT CD, *N* = 7 for WT HF, *N* = 3 for KO CD, and *N* = 5 for KO HF. Data are shown as mean ± SEM. Significance was determined by two-way ANOVA with Tukey’s multiple comparisons test. *p < .05, **p < .01. **(e)** Early life dietary high glucose exposure in WT and *Slc2a5*^*-/-*^ mice. Schematic of wildtype (WT) and *Slc2a5*^*-/-*^ (KO) dams placed on a third isocaloric diet, 15% kcal% glucose diet (HG), for at least one week prior to pregnancy and maintained through gestation and lactation. Brains from postnatal day 7 (P7) mice were collected and analyzed using confocal microscopy. For weight measurements shown in Extended Data Fig. 4b, 8-10-week-old adults separated by sex were placed on all three isocaloric diets in Fig. 1a and here for 3 weeks and weighed daily. **(f, g)** Maternal high fructose diet decreases microglia density. Representative images **(f)** and quantitation **(g)** of microglia (Iba1+) numbers in the prefrontal cortex from P7 WT and KO mice exposed to CD or HF. Confocal microscopy (20x objective, 20 μm thick image stacks with 2 μm step-size) was used to obtain imaging data. Number of microglia were counted in four FOVs (fields of view) across two tissue sections per mouse for each condition in Fiji, and each dot represents the mean for one animal, with *N* = 11 for WT CD, *N* = 17 for WT HF, *N* = 18 for KO CD, and *N* = 13 for KO HF. Scale bar, 100 µm. Data are shown as mean ± SEM. Significance was determined using two-way ANOVA with Tukey’s multiple comparisons test. **p < .01, ***p < 0.001, ****p < .0001.

**Extended Data Fig. 2:**
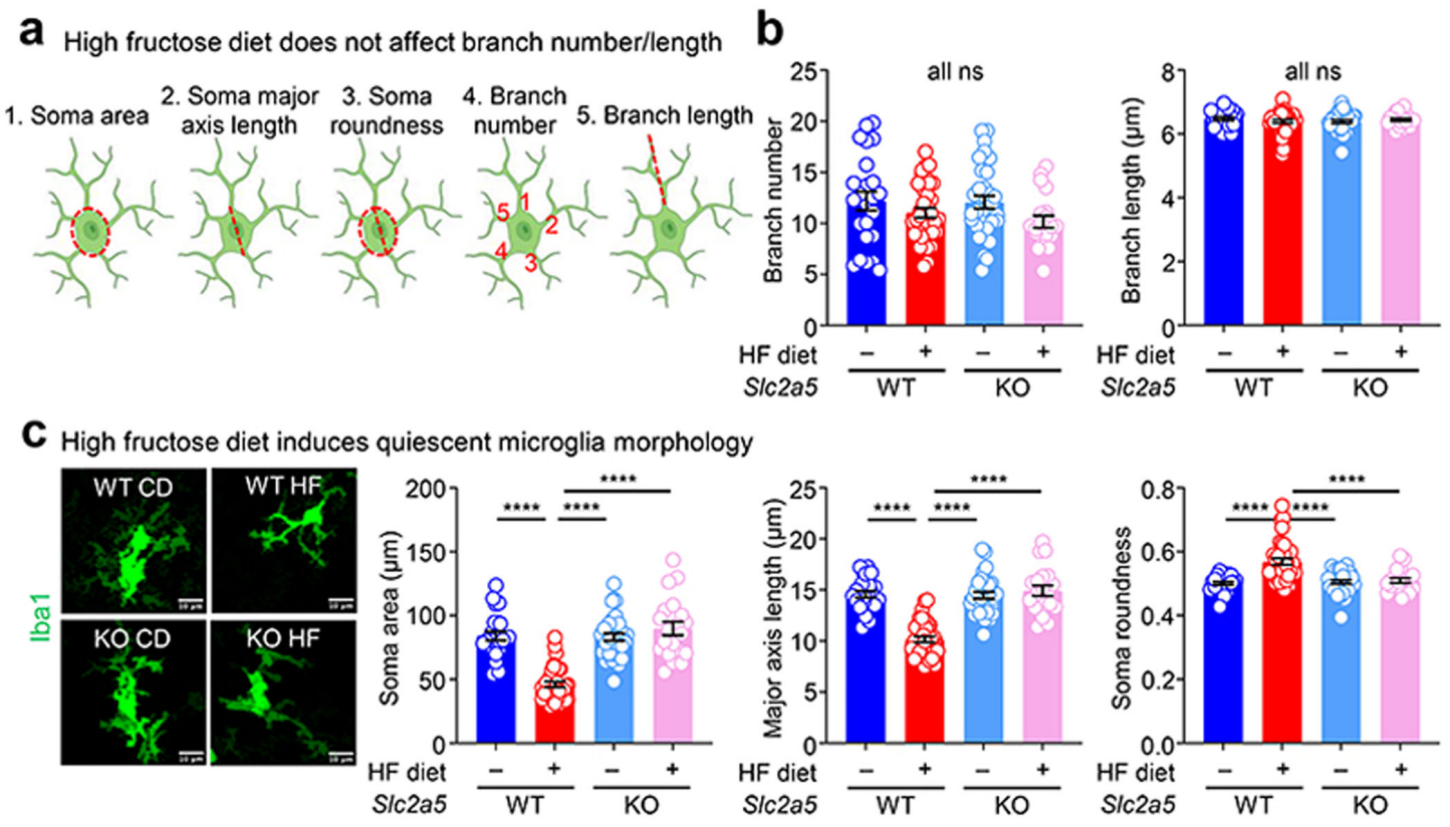
The effect of high fructose on neonatal microglia morphology *in vivo*. **(a)** Schematics of microglial morphological features analyzed. Five features shown are highlighted in red. **(b)** High fructose diet does not affect branch number or length. Quantitation of branch number (4) and branch length (5) of microglia in the prefrontal cortex of P7 wildtype (WT) and *Slc2a5*^–/–^ (KO) neonates born to dams on control diet (CD) or high fructose diet (HF) based on analysis of all cells from four fields of view (FOVs) per mouse obtained by confocal microscopy as in Fig. 1b. *N* = 24 for WT CD, *N* = 36 for WT HF, *N* = 32 for KO CD, and *N* = 20 for KO HF with *n* = 1056 to 1907 cells per condition. Each dot represents one cell whose morphological features were analyzed in a semi-automated process described in the Methods. Data are shown as mean ± SEM. Significance was determined by two-way ANOVA with Tukey’s multiple comparisons test. ns = not significant. **(c)** Maternal high fructose diet induces quiescent microglia morphology. Representative max intensity z-stack projections (left) of microglia in the prefrontal cortex from WT and KO P7 mice exposed to maternal CD or HF used to analyze microglia morphology. Quantitation of soma area (left graph), soma major axis length (middle), and soma roundness (right) from microglial morphological analysis of all cells from four FOVs per animal obtained by confocal microscopy as in Fig. 1b. *N* = 24 for WT CD, *N* = 36 for WT HF, *N* = 32 for KO CD, and *N* = 20 for KO HF with 1056 to 1907 cells per condition. Each dot represents one cell whose morphological features were analyzed in a semi-automated process described in the Methods. Data are shown as mean ± SEM. Significance was determined by two-way ANOVA with Tukey’s multiple comparisons test. ****p < .0001. Scale bar, 10 µm.

High fructose consumption, either by pregnant mothers or during adolescence, is correlated with neurodevelopmental sequalae including mood and anxiety disorder development. To test whether high fructose exposure directly affects microglia, the CNS-resident phagocyte essential for neurodevelopment, we delivered fructose to neonates using intragastric injection (Extended Data Fig. 1a). We focused on the prefrontal cortex (PFC) of neonatal mice because the PFC accounts for a significant amount of the brain volume at this early stage and its healthy development is critical for motor, cognitive, affective, and social behavior in mice and humans^14, 15^. Additionally, defective Pavlovian fear extinction is strongly associated with the development of mood and anxiety disorders^16^ and a key region in this neural circuit is the PFC^17^. Strikingly, we observed significant reductions in microglia numbers and increased numbers of uncleared dying cells in neonates treated with high fructose compared to vehicle-treated neonates (Extended Data Fig. 1b-d). We next queried whether this phenotype would extend to neonates born to dams on high fructose. This is especially important, because a recent study found that fructose is transmitted via breast milk and that increased maternal fructose consumption (e.g., via sugar-sweetened beverages or juice) correlates with negative neurodevelopmental outcomes^18, 19^. Here, we placed mouse dams on isocaloric diets containing either 0 kcal% sucrose/0 kcal% fructose (control diet, CD) or clinically-relevant 15 kcal% high fructose diet (HF) that more accurately reflects average consumption in the United States^20^ (Extended Data Fig. 1e). We then analyzed the prefrontal cortex (PFC) in brains of offspring at perinatal day 7 (P7), which represents the key period where microglia-mediated clearance of dying neurons peaks^9^. We found that neonates born to and nursed by dams on HF diet had significantly fewer microglia compared to mice born to and nursed by dams given control diet (Extended Data Fig. 1f,g). We also observed altered cell morphology in neonates born to and nursed by dams on HF diet using a battery of approaches based on literature defining microglia morphological changes indicative of cellular function (and dysfunction; Extended Data Fig. 2a, see Method). Using this battery, we found that microglia in neonates born to and nursed by dams on HF diet exhibit decreased soma size, decreased major axis length, and increased soma roundness (Extended Data Fig. 2b,c), all previously shown as phenotypes associated with quiescent, non-phagocytic microglia^21^. Contrarily, we did not detect significant changes in microglia branching or branch junctions, which are not associated with neonatal microglia phagocytic activity (Extended Data Fig. 2b).

**Extended Data Fig. 3:**
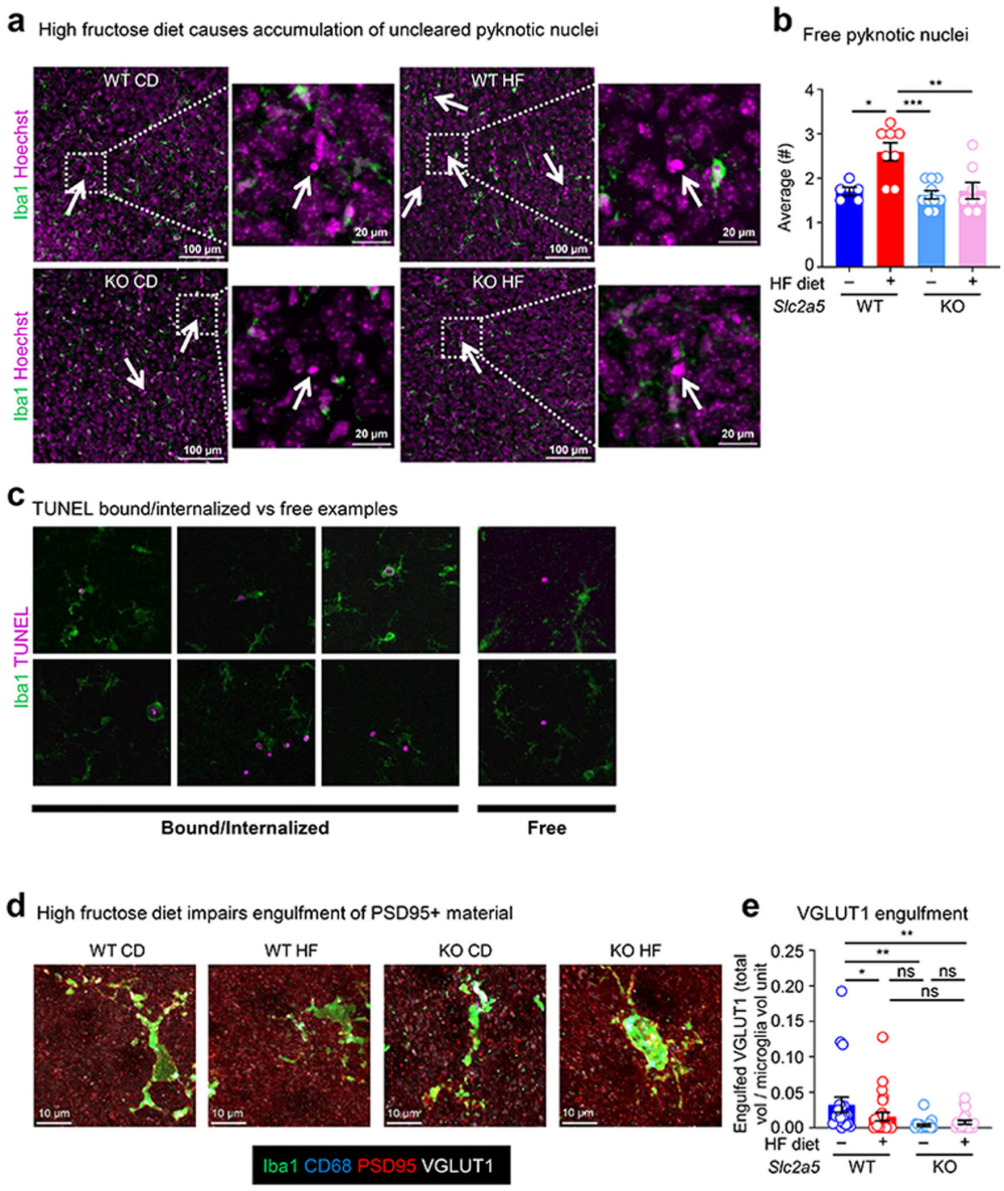
Additional assessments of microglia phagocytosis under high fructose exposure *in vivo*. **(a, b)** High fructose diet causes accumulation of uncleared pyknotic nuclei. Representative images **(a)** and quantitation **(b)** of the prefrontal cortex from wildtype (WT) and *Slc2a5*^–/–^ (KO) P7 neonates exposed to maternal CD or HF in Fig. 1b showing Iba1 staining of microglia and Hoechst staining of nuclei. Arrows denote condensed chromatin of apoptotic cells by pyknotic nuclei accumulation. Scale bars, 100 µm (inlay, 20 µm). **(b)** Quantitation of average number of free pyknotic nuclei in **(a)**, from four FOVs per mouse, with each dot representing the mean for one mouse, with *N* = 5 for WT CD, *N* = 8 for WT HF, *N* = 10 for KO CD, and *N* = 8 for KO HF. Data are shown as mean ± SEM. Significance was determined by two-way ANOVA with Tukey’s multiple comparisons test. *p < .05, **p < .01, ***p < .001. **(c)** Distinguishing bound or internalized versus free TUNEL. Representative images of both Iba1+ microglia associated with TUNEL (bound or internalized) and free TUNEL+ puncta from experiments performed in Fig. 1. **(d)** High fructose diet does not significantly affect engulfment of VGLUT1 material. Representative max intensity z-projections (left) corresponding to 3D surface reconstruction performed in Fig. 1e-f using Imaris, and quantitation (right) of Iba1+ microglia (green) containing engulfed VGLUT1 puncta (white) within CD68+ phagolysosomal structures (blue), from the prefrontal cortex of P7 WT and KO mice exposed to CD or HF. Confocal microscopy (63x oil immersion objective, 20 μm thick image stacks with 0.3 μm step-size) was used to obtain imaging data for Imaris. For quantitation, engulfed VGLUT1 material **(e)** was normalized to Iba+ cell volume using Imaris. Data are from *N*=4 WT CD, *N*=5 WT HF, *N*=5 KO CD, *N*=5 KO HF mice with points representing individual cells (*n* = 21, 25, 21, and 21, respectively). Data are shown as mean ± SEM. Significance was determined using two-way ANOVA with Tukey’s multiple comparisons test. ns = not significant, *p < .05, **p < .01. Scale bar, 10μm with 4x digital zoom.

**Extended Data Fig. 4:**
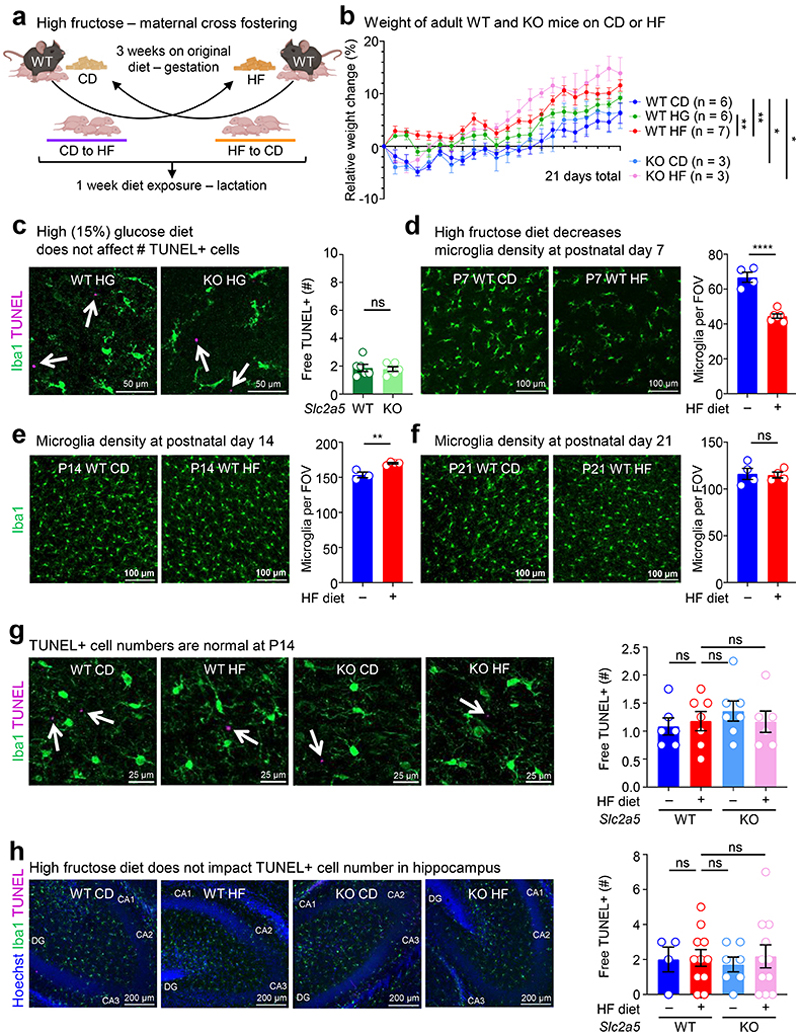
Additional analyses of microglia numbers and function at different times post-birth and brain regions. **(a)** Schematic of cross-fostering experiments. Timed matings were used for cross-fostering experiments, in which litters born to WT dams on high fructose diet were switched with litters born to dams on control diet on P0. These litters were either exposed to high fructose during gestation (~3 weeks) but control diet during lactation (1 week) or were exposed to control diet during gestation but high fructose diet during lactation. **(b)** High fructose diet marginally increases the weight of both WT and KO mice. For weight measurements, 8–10-week-old adults separated by sex were placed on one of three isocaloric diets (CD = control diet, HG = high glucose, HF = high fructose) for 3 weeks and weighed daily, with *N* = 6 for WT CD, *N* = 7 for WT HF, *N* = 3 for KO CD, *N* = 3 for KO HF, and *N* = 6 for WT HG. Data are shown as mean ± SEM. Significance was determined using two-way ANOVA with Tukey’s multiple comparisons test of area under the curve (AUC) for each cohort. *p < .05, **p < .01. **(c)** Maternal high glucose diet does not affect the number of TUNEL+ cells. Representative images (left) and quantitation (right) of uncleared (‘free’) TUNEL+ puncta in the prefrontal cortex of P7 WT and KO mice exposed to CD or HG as shown in Extended Data Fig. 1e. Confocal microscopy (20x objective, 20 μm thick image stacks with 2 μm step-size) was used to obtain imaging data. Free and bound/internalized TUNEL+ puncta (see Extended Data Fig. 3c) were identified in four FOVs (fields of view) across two tissue sections per mouse for each condition, and each dot represents the mean for one mouse, with *N* = 6 mice per condition. Data are shown as mean ± SEM. Significance was determined using an unpaired t-test. ns = not significant. Scale bar, 50 μm. **(d)** Maternal high fructose diet decreases microglia density at postnatal day 7. Representative images (left) and quantitation (right) of Iba1+ microglia in the prefrontal cortex of P7 WT mice exposed to CD or HF, obtained before Extended Data Fig 1f which includes genetic deletion of GLUT5. Confocal microscopy (20x objective, 20 μm thick image stacks with 2 μm step-size) was used to obtain imaging data. Number of microglia were counted in four FOVs (fields of view) across two tissue sections per mouse for each condition in Fiji, and each dot represents the mean for one mouse, with *N* = 4 for WT CD and *N* = 5 for WT HF. Data are shown as mean ± SEM. Significance was determined using an unpaired t-test. ****p < .0001. Scale bar, 100 μm. **(e)** Maternal high fructose slightly increases microglia density at postnatal day 14. Representative images (left) and quantitation (right) of Iba1+ microglia in the prefrontal cortex of P14 WT mice exposed to CD or HF. Confocal microscopy (20x objective, 20 μm thick image stacks with 2 μm step-size) was used to obtain imaging data. Number of microglia were counted in four FOVs (fields of view) across two tissue sections per mouse for each condition in Fiji, and each dot represents the mean for one mouse, with *N* = 3 for WT CD and *N* = 4 for WT HF. Data are shown as mean ± SEM. Significance was determined using an unpaired t-test. **p < .01. Scale bar, 100 μm. **(f)** Maternal high fructose has no effect on microglia density at postnatal day 21. Representative images (left) and quantitation (right) of Iba1+ microglia in the prefrontal cortex of P21 WT mice exposed to CD or HF. Confocal microscopy (20x objective, 20 μm thick image stacks with 2 μm step-size) was used to obtain imaging data. Number of microglia were counted in four FOVs (fields of view) across two tissue sections per mouse for each condition in Fiji, and each dot represents the mean for one mouse, with *N* = 4 mice per condition. Data are shown as mean ± SEM. Significance was determined using an unpaired t-test. ns = not significant. Scale bar, 100 μm. **(g)** Maternal high fructose does not impact TUNEL+ cell numbers at postnatal day P14. Representative images (left) and quantitation (right) of uncleared (‘free’) TUNEL+ puncta in the prefrontal cortex of P14 WT and KO mice exposed to CD or HF. Confocal microscopy (20x objective, 20 μm thick image stacks with 2 μm step-size) was used to obtain imaging data. Free and bound/internalized TUNEL+ puncta (see Extended Data Fig. 3c) were identified in four FOVs (fields of view) across two tissue sections per mouse for each condition, and each dot represents the mean for one mouse, with *N* = 6 for WT CD, *N* = 7 for WT HF, *N* = 7 for KO CD, and *N* = 6 for KO HF. Data are shown as mean ± SEM. Significance was determined using two-way ANOVA with Tukey’s multiple comparisons test. ns = not significant. Scale bar, 25 μm. **(h)** Maternal high fructose does not impact TUNEL+ cell numbers in the hippocampus at postnatal day P7. Representative images (left) and quantitation (right) of uncleared (‘free’) TUNEL+ puncta in the hippocampus of P14 WT and KO mice exposed to CD or HF. Confocal microscopy (10x objective) was used to obtain images encompassing the CA1, CA2, and CA3 regions within one FOV, with free TUNEL+ puncta identified in one hippocampal region per mouse (further magnification and z-axis movement was performed as necessary for resolution purposes). Each dot represents the quantification of one mouse’s hippocampal region, with *N* = 4 for WT CD, *N* = 11 for WT HF, *N* = 7 for KO CD, and *N* = 11 for KO HF. Data are shown as mean ± SEM. Significance was determined using two-way ANOVA with Tukey’s multiple comparisons test. ns = not significant. Scale bar, 200 μm.

**Fig. 1:**
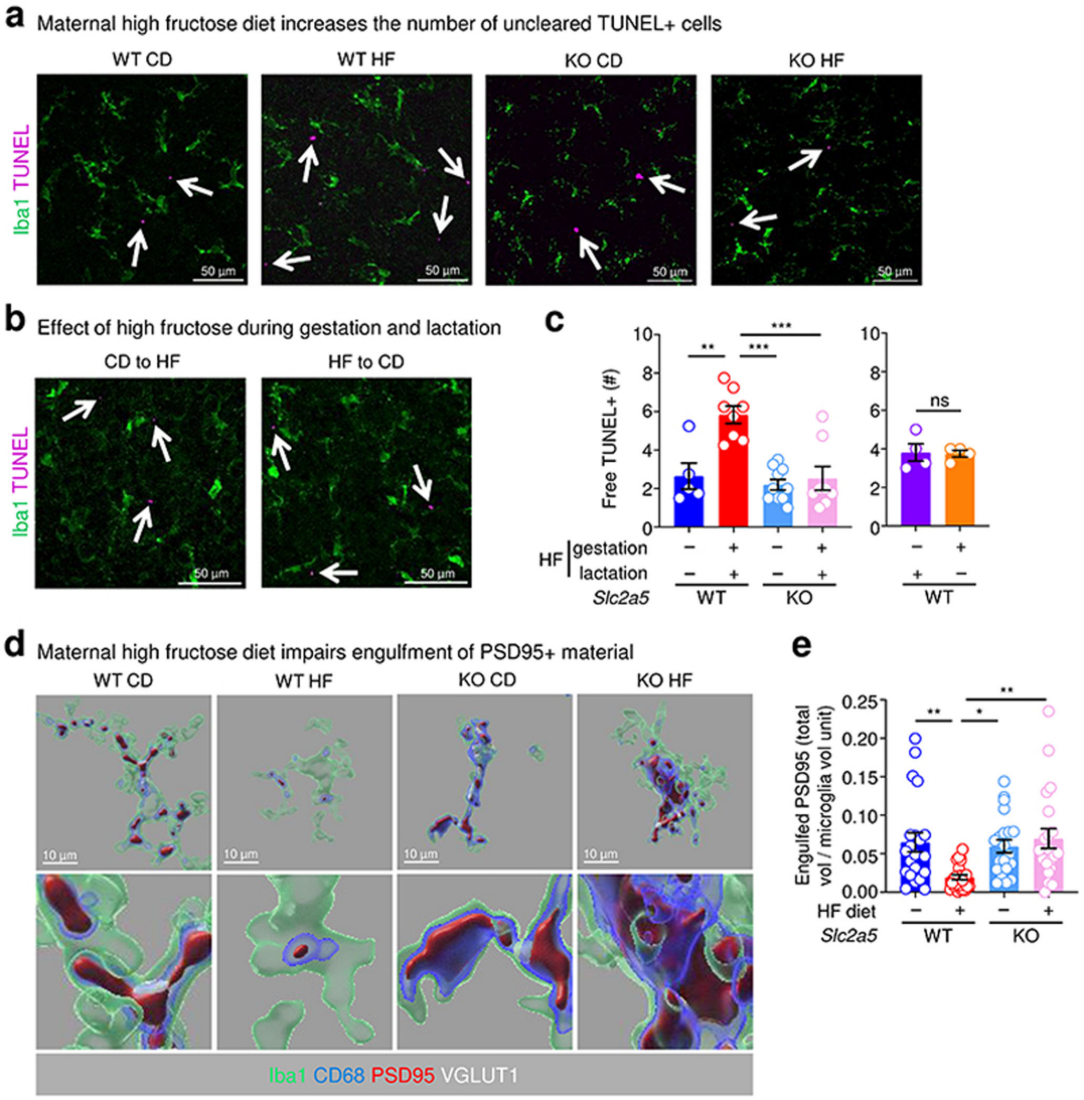
Early life high fructose exposure suppresses microglial phagocytosis *in vivo*. **(a)** Representative images of uncleared (‘free’) TUNEL+ puncta in the prefrontal cortex (PFC) of WT and KO P7 mice exposed to CD or HF diet. Confocal microscopy (20x objective, 20 μm thick image stacks with 2μm step-size) was used to obtain imaging data. Free TUNEL+ puncta were identified in four FOVs across two tissue sections per mouse/condition. Scale bar, 50μm. **(b, c)** Representative images (**b**) and summary (**c**) from PFC of P7 WT and KO mice exposed to HF diet during gestation or lactation. Free TUNEL+ puncta were identified in four FOVs across two tissue sections per mouse/condition. Each data point represents the mean for one mouse, with *N*=5 WT CD, *N*=8 WT HF, *N*=10 KO CD, *N*=8 KO HF, *N*=4 gestation, and *N*=4 lactation mice. Scale bar, 50μm. **(d, e)** 3D surface reconstruction of confocal microscopy **(d)** showing representative Iba1+ microglia containing engulfed PSD-95 puncta within CD68+ phagolysosomals in PFC of P7 WT and KO mice exposed to CD or HF. Quantitation of engulfed PSD-95 material **(e)** was normalized to Iba+ cell volume using Imaris. Data are from *N*=4 WT CD, *N*=5 WT HF, *N*=5 KO CD, *N*=5 KO HF mice with points representing individual cells (*n* = 21, 25, 21, and 21, respectively). Scale bar, 10μm, 4x digital zoom. Data are shown as mean±SEM. Significance was determined using two-way ANOVA with Tukey’s post-hoc or unpaired t-test. ns = not significant, *p<.05, **p<.01, ***p<.001.

We next examined whether neonates born to and nursed by dams on HF diet also display evidence of phagocytosis defects. Indeed, we observed a significant increase in uncleared (‘free’) pyknotic (dying cell) nuclei in neonates born to and nursed by dams on HF diet (Extended Data Fig. 3a, b). As an orthogonal approach to determine if microglia phagocytosis is disrupted in neonates born to and nursed by dams on HF diet, we took two complimentary approaches. First, we used the terminal deoxynucleotidyl transferase dUTP nick end labeling (TUNEL) assay to identify uncleared dead cells (Extended Data Fig. 3c). Second, we analyzed microglia for the presence of postsynaptic density protein 95 (PSD-95) material, an established readout for phagocytosis of neuronal debris^22^. Similar to our results showing a significant increase in uncleared pyknotic nuclei, neonates born to and nursed by dams on HF diet exhibited a significant increase in uncleared TUNEL-positive cells (Fig. 1a-c). Interestingly, the effect of high fructose on microglia phagocytosis was imparted during both gestation and lactation, as both neonates born to dams on high fructose but nursed by dams on normal diet and neonates born to dams on normal diet but nursed by dams on high fructose diet exhibited significant defects in microglia phagocytosis, albeit both lower than neonates born to and nursed by dams on high fructose diet (Fig. 1b,c; Extended Data Fig. 4a). Additionally, significantly fewer microglia in neonates born to and nursed by dams on HF diet contained PSD-95 material and contained significantly less engulfed PSD-95+ material on a per-cell basis (Fig. 1d,e and Extended Data Fig. 3d,e), further supporting the hypothesis that HF diet suppresses microglia phagocytic activity. On the other hand, we observed no significant difference in microglia phagocytic activity in neonates born to and nursed by dams on high glucose diet (Extended Data Fig. 4b,c), suggesting that the effect of HF diet on microglia phagocytic activity is specifically due to fructose and not because of a non-specific effect of sugar overabundance. Finally, the effect of HF diet on microglia numbers and phagocytic activity in neonates appeared to be restricted to the developing cortex (especially prefrontal cortex) during this early critical window (Extended Data Fig. 4d-h). Taken together, our results suggest that microglia in neonates exposed to high fructose have disrupted phagocytic function *in vivo*.

### The effect of early life high fructose exposure depends on GLUT5-mediated fructose transport in neonates

**Extended Data Fig. 5:**
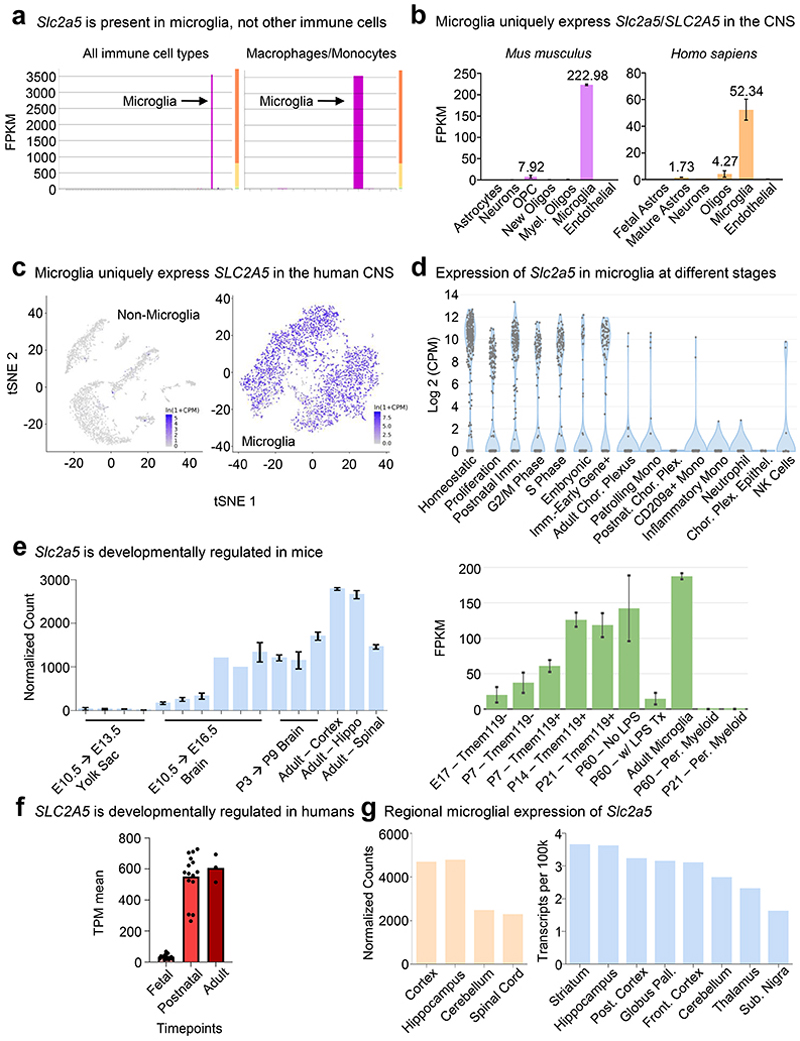
Secondary data analysis of mouse and human microglia RNA-seq datasets. **(a-g)** Unique expression of *Slc2a5* in the mouse CNS and *SLC2A5* in the human CNS. *Slc2a5/SLC2A5* expression plotted using data in published and publicly available mouse and human datasets from the following references (see references section for full details). **(a)** Immgen.org, 2020 – *Slc2a5* expression in all immune cell types (left) and in macrophages and monocytes (right). **(b)** Zhang et al., J. Neurosci., 2014 (left) and Zhang et al., Neuron, 2016 (right) – *Slc2a5/SLC2A5* expression in microglia compared to other CNS cells in mouse (left) and human (right). Data are shown as mean ± SD. **(c)** The Tabula Muris Consortium, Nature, 2018 – *SLC2A5* expression in human microglia and non-microglia cells in the CNS. **(d)** Li et al., Neuron, 2019 – *Slc2a5* expression in microglia at different stages. **(e)** Matcovitch-Natan et al., Science, 2016 (left) and Bennett et al., PNAS, 2016 (right) – *Slc2a5* expression in the mouse CNS during development (left) and in TMEM119+ microglia at P7, P14, P21, and P60 (right). Data are shown as mean ± SD. **(f)** adapted from Han et al., Immunity, 2023 – Enhanced *SLC2A5* expression in human microglia at postnatal timepoints compared to fetal timepoints. **(g)** Immgen.org, 2020 (left) and Saunders et al., Cell, 2018 (right) – regional expression of *Slc2a5* in mouse microglia.

**Extended Data Fig. 6:**
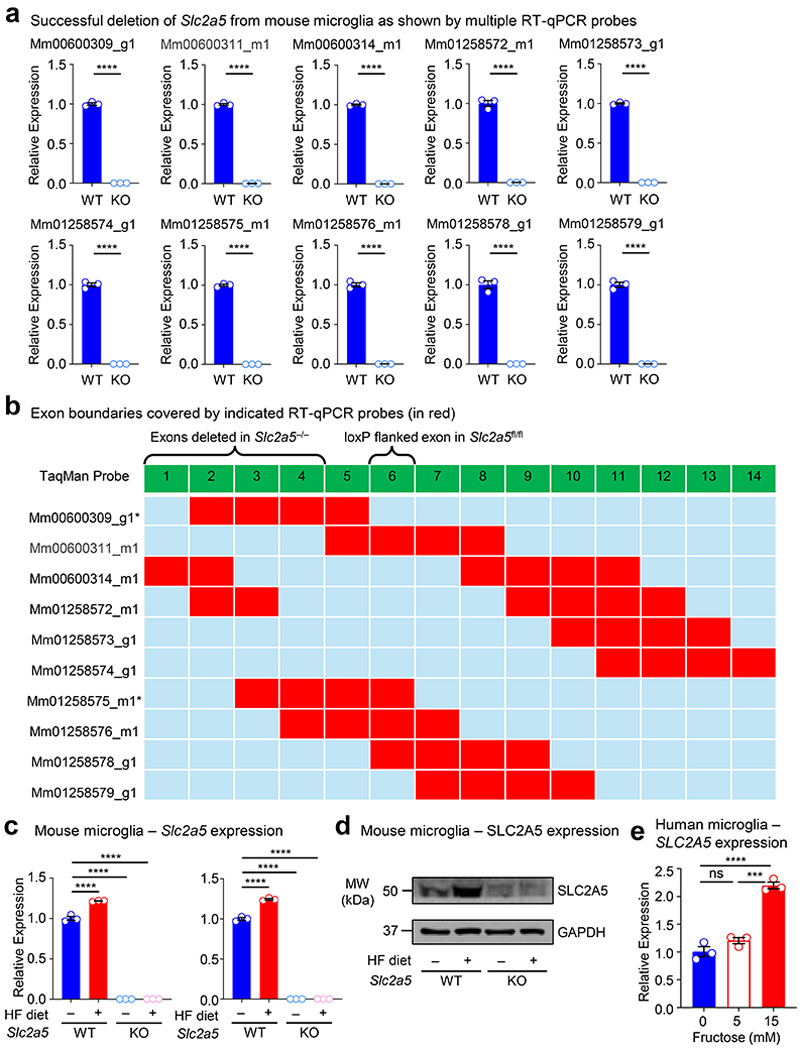
Evaluation of *Slc2a5/SLC2A5* transcription and SLC2A5 protein levels in microglia. **(a)** Absence of *Slc2a5* in murine microglia confirmed by RT-qPCR. All commercially available TaqMan probes were tested using P2-P4 neonatal microglia isolated from WT and *Slc2a5*^*-/-*^ (KO) mice. Each dot represents one mouse. Data are shown as mean ± SEM. Significance was determined using a two-tailed, unpaired t-test. ****p < .0001. **(b)** Coverage of murine *Slc2a5* TaqMan probes spanning all 14 exons. The first four exons of *Slc2a5* are deleted in *Slc2a5*^–/–^ (KO) mice and exon 6 is flanked by loxP sites in *Slc2a5*^fl/fl^ CSF1R-Cre (cKO) mice. Indicated probes (*) were further validated in Fig. 2a and Extended Data Fig. 6c using P2-P4 neonatal microglia isolated from WT and KO mice exposed to CD or HF. **(c)** High fructose exposure enhances *Slc2a5* expression in neonatal microglia. Microglia from P7 wildtype (WT) and *Slc2a5*^–/–^ (KO) neonates born to and nursed by dams on control diet (CD) or high fructose diet (HF) as in Fig. 1 were isolated for analysis of *Slc2a5* expression via RT-qPCR using the probes Mm00600309_g1 and Mm01258575_m1, then normalized to 18s. Each dot represents one mouse (n=3 per condition). Data are shown as mean ± SEM. Significance was determined using two-way ANOVA with Tukey’s multiple comparisons test. ****p < .0001. **(d)** High fructose exposure enhances SLC2A5 protein levels in neonatal microglia. Experiments were performed as in **(c)** but microglia from 4 mice were pooled per lane and processed for analysis via western blot. Expression of SLC2A5 with GAPDH as a loading control is shown, representative of two independent experiments. For gel source data, see Supplementary Figure 1. **(e)** High fructose exposure enhances *SLC2A5* expression in human pluripotent stem cell (PSC)-derived microglia. Human PSC-derived microglia were cultured in complete RPMI containing 10 mM glucose with either 0 mM fructose and 20 mM mannitol, 5 mM fructose and 15 mM mannitol, or 15 mM fructose and 5 mM mannitol for 6 d and were then harvested for analysis of *SLC2A5* expression via RT-qPCR. Data is from three independent experiments, with expression normalized to 18s. Significance was determined using one-way ANOVA with Tukey’s multiple comparisons test. ****p < .0001.

Fructose transport is achieved by the facilitative glucose transport family members GLUT2 (*Slc2a2*) and GLUT5 (*Slc2a5*)^23^. In adults, GLUT5 mediates transport of dietary fructose from the intestinal lumen across the brush border membrane, followed by GLUT2-mediated transport across the basolateral membrane into the blood^24^. Contrarily, gestational and pre-weaning neonates express little if any GLUT5 but stably express GLUT2, including reportedly in the brush-border membrane^25, 26^, suggesting that fructose delivered to pre-weaning neonates could gain access to the brain independent of GLUT5 in the intestinal lumen. Interestingly, GLUT5 was previously identified as a core microglia signature gene/protein together with TMEM119 and P2Y12^27^. Analysis of previously published informatics data revealed that microglia (including neonatal microglia) are the only immune cell in mice and humans that express GLUT5 (Extended Data Fig. 5a,b) and are the only CNS-resident cell in mice and humans to express GLUT5 (Extended Data Fig. 5c,d). Additionally, microglia GLUT5 is developmentally and regionally regulated in mice and humans, with its expression significantly lower in microglia during development than adulthood (Extended Data Fig. 5e,f) and lower in regions of high microglia phagocytosis than regions of low microglia phagocytosis (Extended Data Fig. 5g). Given this series of observations, we sought to determine if neonatal GLUT5 is necessary for the microglia phenotypes observed in high fructose-treated mice. To this end, we used *Slc2a5* deficient mice in which exons 1-4 are deleted^28^ (*Slc2a5*^–/–^ mice; Extended Data Fig. 6a,b) to perform high fructose diet experiments. Strikingly, microglia numbers, morphology, and phagocytic capacity were completely reversed in neonates lacking GLUT5 born to and nursed by dams on HF diet compared to wildtype neonates born to and nursed by dams on HF diet (Fig. 1a-e; Extended Data Figs. 1-3). Thus, neonatal GLUT5 facilitates the negative effects of early life high fructose on the developing CNS.

### High fructose exposure directly suppresses microglia phagocytosis *in vitro*

**Extended Data Fig. 7:**
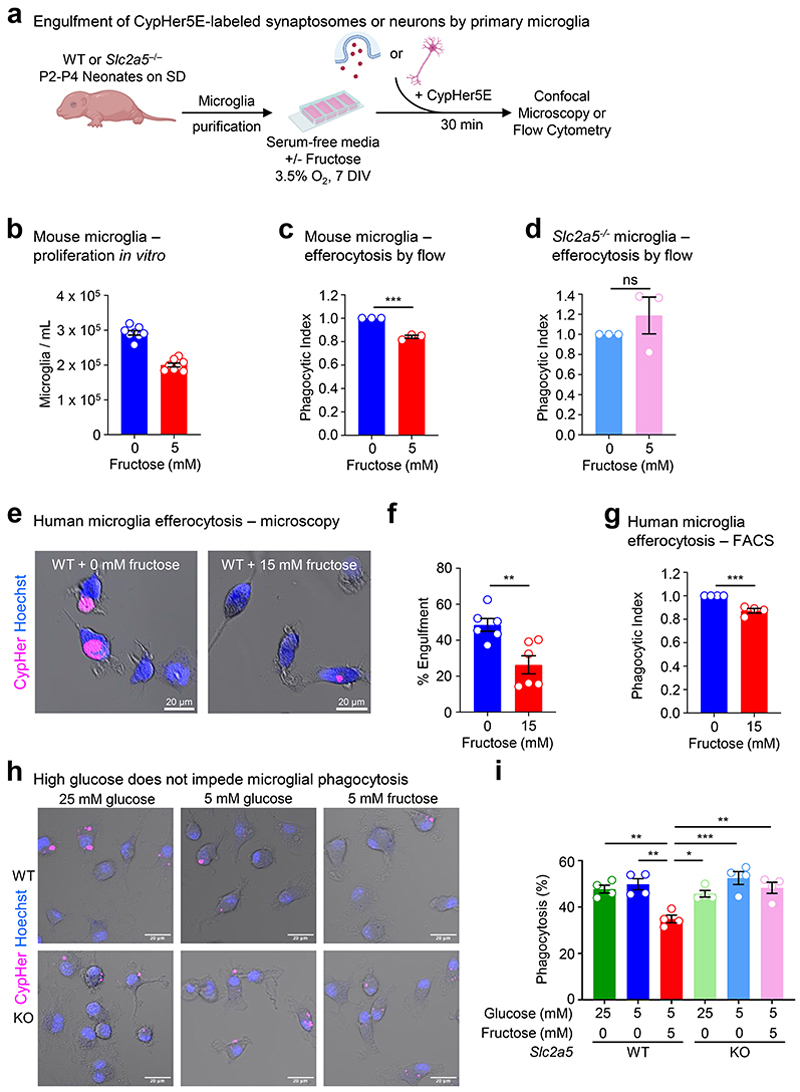
Additional analyses of mouse and human microglia phagocytosis *in vitro*. **(a)** Schematic of assays performed to assess microglia phagocytosis in Fig. 2b-i. Microglia were isolated from P2-P4 WT and KO mice and cultured at physiological oxygen (3.5%) in serum-free TIC media containing 5 mM glucose with either 0 mM fructose and 20 mM mannitol, 1 mM fructose and 19 mM mannitol, or 5mM fructose and 15 mM mannitol for one week. Synaptosomes or apoptotic neural cells were labeled with CypHer5E and then cultured with conditioned microglia for 30 min. Phagocytosis was subsequently analyzed via flow cytometry and/or confocal microscopy. **(b)** High fructose inhibits primary microglial expansion in mixed glial cultures. Mixed glial cultures were generated using dissected cortices from 2 WT P2-P4 neonates to seed each T75 flask. Cultures were grown in 20 mM glucose in the presence or absence of 5 mM fructose. Microglia yield was quantified after 18 d of culture. Each dot represents a culture (mouse), and all 8 cultures from 2 independent experiments are plotted. Data are shown as mean ± SEM. Significance was determined using a two-tailed, unpaired t-test. ****p < .0001. **(c**,**)** High fructose impairs mouse primary microglia efferocytosis. Apoptotic neural cells were generated by treating the neural cell line N2A with 0.25μM staurosporine for 12-14 h and then labeling with CypHer5E. These cells were then incubated with WT primary microglia as described in **(a)** at a 1:1 target:phagocyte ratio for 30 min. Microglia were collected and analyzed via flow cytometry. Data are presented as phagocytic index (percent phagocytosis in experimental microglia divided by percent phagocytosis in control microglia) and encompasses three independent experiments each with three technical replicates per condition. Data are shown as mean ± SEM. Significance was determined using a two-tailed, unpaired t-test. ***p < .001. **(d)** *Slc2a5* deletion rescues impaired phagocytosis of apoptotic neural cells by primary microglia. Experiments were performed similarly to **(c)**, except that primary microglia were isolated from KO mice. Data are presented as phagocytic index and includes three independent experiments with three technical replicates per condition. Data are shown as mean ± SEM. Significance was determined using a two-tailed, unpaired t-test. ns = not significant. **(e, f)** High fructose impedes phagocytosis of apoptotic neural cells by human PSC-derived microglia. Experiments were performed similarly to **(c, d)** except with hPSC-derived microglia instead of mouse microglia, and confocal microscopy was used instead of flow cytometry, and the number of hPSC-derived microglia containing CypHer5E+ apoptotic corpses were quantified in Fiji, with each dot representing one of six FOVs per condition. Data is representative of three independent experiments and are shown as mean ± SEM. Significance was determined using a two-tailed, unpaired t-test. **p < .01. Scale bar, 20 μm. **(g)** hPSC-derived microglia efferocytosis by flow cytometry. Experiments were performed similarly to **(c, d)** except with hPSC-derived microglia and flow cytometry. Data are presented as phagocytic index and includes four independent experiments with three technical replicates per condition. Data are shown as mean ± SEM. Significance was determined using a two-tailed, unpaired t-test. ***p < .001. **(h, i)** High glucose does not impede microglial phagocytosis. Experiments were performed similarly to Fig. 2c-i, except with an additional media condition containing high (25 mM) glucose commonly used in commercially available media formulations. Confocal microscopy and Fiji were used to quantify WT or KO microglia that engulfed CypHer5E-labeled synaptosomes under each of the three media conditions, with 4 FOVs per condition. Each dot represents one FOV, and data is representative of two independent experiments. Data are shown as mean ± SEM. Significance was determined using two-way ANOVA with Tukey’s multiple comparisons test. *p < .05, **p < .01. ***p < .001. Scale bar, 20 μm.

**Fig. 2:**
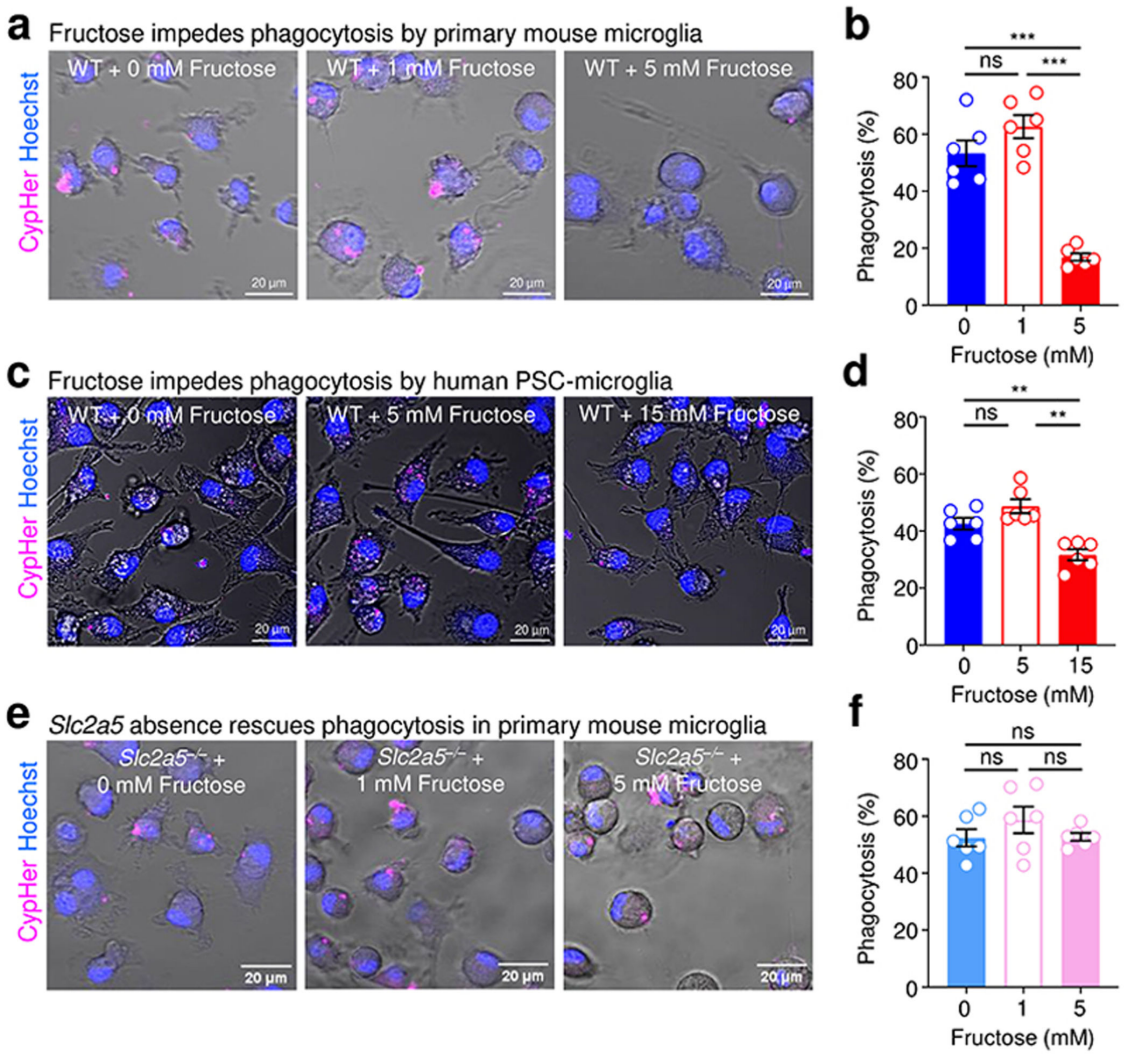
High fructose exposure directly suppresses microglia phagocytosis. **(a, b)** Representative images **(a)** and quantitation **(b)** of microglia phagocytosis of CypHer5E-labeled synaptosomes. CypHer5E+ microglia were identified in six different FOVs per condition and the percentage for each FOV is shown. Data represents three independent experiments. Scale bar, 20μm. **(c, d)** Representative images **(c)** and quantitation **(d)** of hPSC-derived microglia phagocytosis of CypHer5E-labeled synaptosomes. Cypher5E+ microglia were identified in six different FOVs per condition and the percentage for each FOV is shown. Data represents three independent experiments. Scale bar, 20μm. **(e, f)** Representative microscopy images **(h)** and quantitation **(i)** of GLUT5-deficient microglia phagocytosis of CypHer5E-labeled synaptosomes. Cypher5E+ microglia were identified in six different FOVs per condition and the percentage for each FOV is shown. Data represents three independent experiments. Scale bar, 20μm. Data are shown as mean±SEM. Significance was determined using ordinary **(f)** or Brown-Forsythe and Welch’s **(b, d)** one-way ANOVA. ns = not significant, **p<.01, ***p<.001, ****p<.0001.

Given our finding that high fructose affects neonatal microglia form and function *in vivo* and microglia are the only cell to express SLC2A5 (GLUT5) in both the CNS and the immune system at large (Extended Data Fig. 5), we speculated that high fructose was acting directly on microglia via GLUT5. In support of this hypothesis, microglia from neonates born to and nursed by dams on HF diet exhibited a significant increase in *Slc2a5* (Extended Data Fig. 6c) and SLC2A5 (Extended Data Fig. 6d) expression compared to microglia from neonates born to and nursed by dams on control diet. To address this hypothesis directly, we next tested the effect of high fructose on primary microglia function at a physiological level of oxygen *in vitro*. Specifically, we used a dedicated chamber system to culture microglia at a constant 3.5% O_2_, consistent with the oxygen level observed in the CNS parenchyma where microglia reside (~3-4% O_2_)^29^. Furthermore, we used an established media formulation that excludes serum because serum exposure has been shown to artificially affect phagocytosis^30^. Primary microglia isolated from P2-P4 neonates were cultured in a closed-state level of low (1 mM) and high (5 mM) fructose with a hexose osmolarity control (mannitol) in fixed physiological glucose (5 mM) for 7 days (Extended Data Fig. 7a). Similar to our *in vivo* observations, mixed glial cultures cultured with high fructose generated fewer numbers of primary mouse microglia (Extended Data Fig. 7b). We next assessed microglia phagocytosis of two key targets: synaptic terminals (synaptosomes) and apoptotic neurons (Extended Data Fig. 7a). Strikingly, we observed a drastic decrease in phagocytosis of both synaptosomes (Fig. 2a,b) and apoptotic neurons (Extended Data Fig. 7c) by primary mouse microglia cultured in high fructose compared to microglia cultured in control media or low fructose. These findings are important because even modest reductions in apoptotic neuron clearance by microglia can have catastrophic effects on neurodevelopment^13, 31, 32^.

Next, we queried whether high fructose has a similar direct effect on human microglia. To test this, we derived microglia-like cells from human pluripotent stem cells (hPSCs) and cultured resultant cells in fructose conditions that significantly induce *SLC2A5* upregulation (Extended Data Fig. 6e). Importantly, hPSC-derived microglia-like cells cultured in high fructose exhibited significantly decreased phagocytosis of synaptosomes (Fig. 2c,d) and apoptotic neurons (Extended Data Fig. 7e-g), similar to mouse primary microglia cultured in high fructose. Taken together, high fructose directly suppresses phagocytosis capacity by both mouse and human microglia.

Finally, it is possible that the effect of high fructose on microglia is unrelated to fructose transport and metabolism itself, especially because the use of closed-state cultures requires the use of higher levels of sugars. To test this, we first isolated primary microglia from GLUT5-deficient neonates and analyzed phagocytosis capacity under high fructose conditions. Importantly, GLUT5 deficiency completely reversed the decreased phagocytosis of synaptosomes (Fig. 2e,f) and apoptotic neurons (Extended Data Fig. 7d) observed in primary microglia cultured in high fructose. Thus, the phagocytosis-suppressive effect of high fructose on microglia is specific for fructose and mediated by the primary fructose transporter GLUT5.

### High fructose directly alters microglial metabolism

**Fig. 3:**
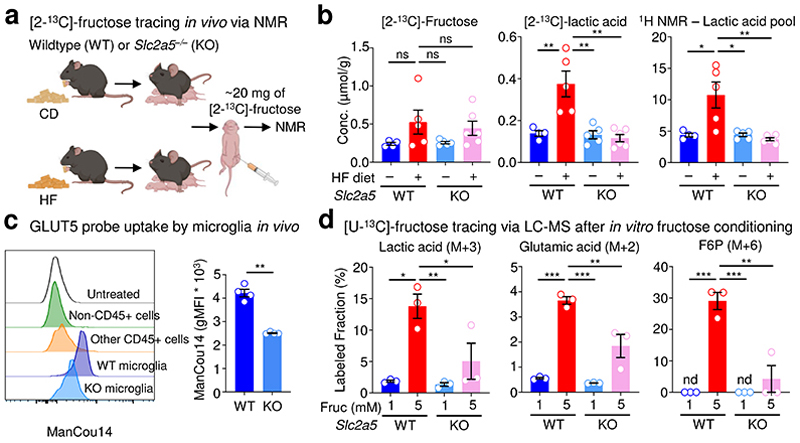
High fructose directly alters microglial metabolism. **(a, b)** (**a**) Wildtype (WT) and *Slc2a5*^–/–^ (KO) P7 mice born to and nursed by dams on control diet (CD) or high fructose (HF) diet, injected intraperitoneally with 4 g/kg [2-^13^C]-fructose, then analyzed via carbon (^13^C) and proton (^1^H) NMR. (**b**) Quantitation of [2-^13^C]-fructose, [2-^13C^]-lactic acid, and total lactic acid pool from *N*=4 WT CD, *N*=5 WT HF, *N*=5 KO CD, and *N*=5 KO HF. Data shown as mean±SEM. Significance was determined by two-way ANOVA with Tukey’s post-hoc. ns = not significant, *p<.05, **p<.01. **(C)** Flow cytometry analysis of ManCou14 in 8-10-week-old WT and KO mice in non-immune cells (CD45-), non-myeloid immune cells (CD45^hi^CX3CR1-CD11b-), and microglia (CD45^int^CX3CR1+CD11b+), with *N*=4 mice per condition. Data shown as mean±SEM and significance was determined by Welch’s t-test. **p<.01. **(D)** Microglia were isolated from P2-P4 wildtype (WT) and *Slc2a5*^–/–^ (KO) mice and cultured at 3.5% O_2_ in indicated conditions for one week, then cultured with [U-^13^C]-fructose for 24h and analyzed using LC-MS. Shown is the fractional enrichment of labeled lactic acid, glutamic acid, and fructose 6-phosphate (F6P). See also Extended Data Fig. 9 for additional analyses. Each dot represents a biological replicate obtained by pooling microglia purified from littermates, with a total of three biological replicates per condition. Data are shown as mean±SEM. Significance was determined by two-way ANOVA with Tukey’s post-hoc. *p<.05, **p<.01, ***p<.001.

Whether fructose metabolism occurs in the CNS, especially in adults, remains an open question and may depend on a series of factors, including the technology used to measure fructose metabolism, what approach is used/which metabolites are measured, and the timing at which one looks. Given our finding that high fructose directly affects microglia phagocytosis, we sought to first determine if we could detect and quantify fructose metabolism in the neonatal brain. We turned to [2-^13^C]-fructose carbon nuclear magnetic resonance (NMR) because of its ability to rapidly and directly quantify fructose metabolism *in vivo*^33, 34^. We injected [2-^13^C]-fructose into the intraperitoneal cavity of wildtype or GLUT5-deficient P7 neonates born to and nursed by dams on HF diet or control diet 15 min prior to analysis (Fig. 3a), which allowed us to accurately measure fructose metabolism in the CNS while limiting the potential confound of fructose conversion in the small intestine or liver^35^. Along with detecting appreciable labeled fructose in the brain across all conditions, we found increased catabolism of [2-^13^C]-fructose in the brains of wildtype neonates born to and nursed by dams on HF diet compared to wildtype neonates born to and nursed by dams on control diet ([2-^13^C]-lactic acid; Fig. 3b). This increased fructose catabolism was commensurate with an increase in the total lactate pool observed only in wildtype neonates born to and nursed by dams on HF diet (Fig. 3b). Importantly, both the increased catabolism of [2-^13^C]-fructose and the increased total lactate pool observed in wildtype neonates born to and nursed by dams on HF diet was completely reversed in neonates born to and nursed by dams on HF diet lacking GLUT5 (Fig. 3b). Thus, fructose catabolism is observed in the brains of neonates on HF diet in a GLUT5-dependent manner.

**Extended Data Fig. 8:**
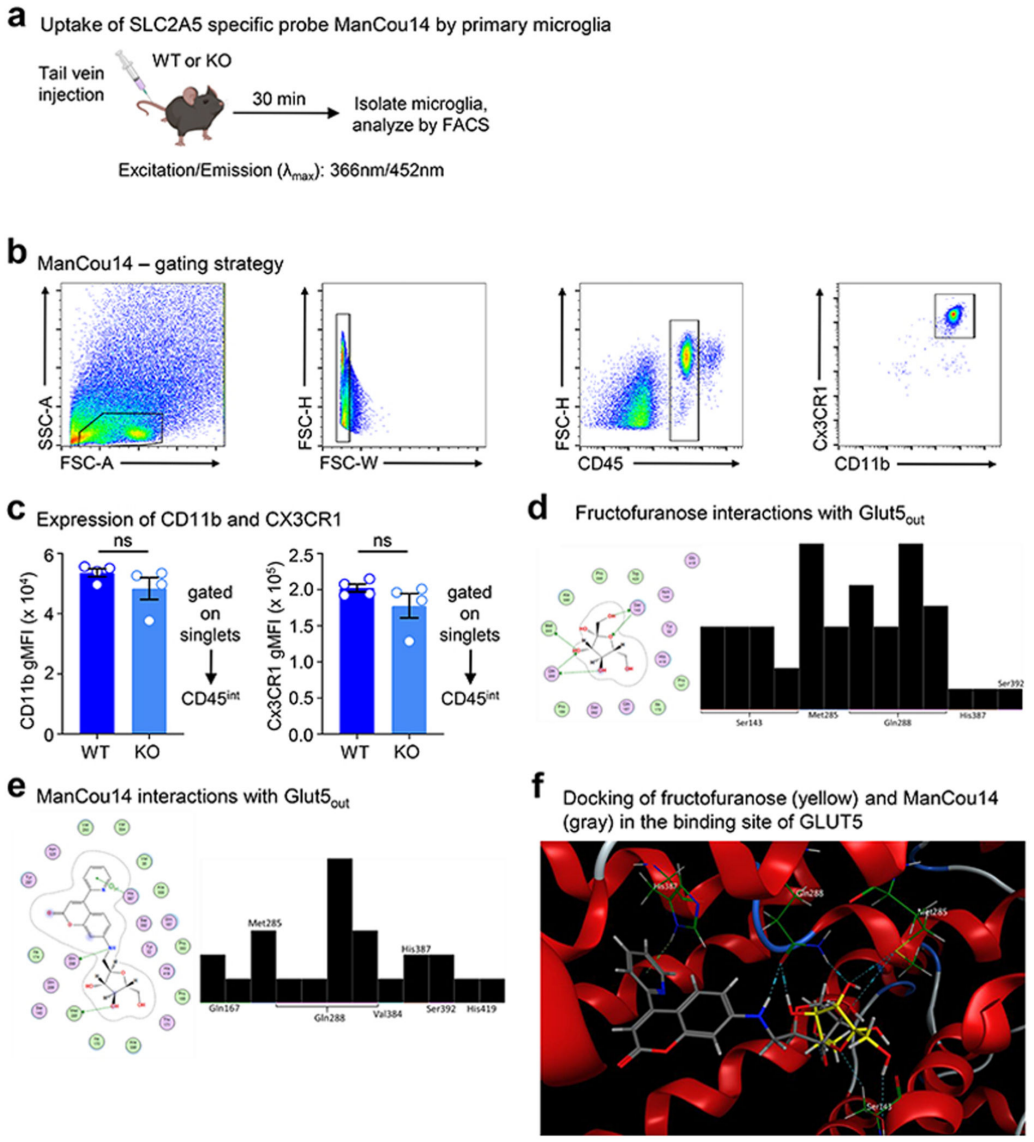
Analysis of ManCou14 uptake by microglia *in vivo*. **(a)** Schematic of experiment using the GLUT5-specific fluorescent probe ManCou14. 1mM ManCou14 was obtained by diluting a 10mM stock with PBS. 200μL was injected via tail vein into 8-10-week-old WT and KO mice, and microglia were isolated 30 min later for flow cytometry. **(b)** Gating strategy to obtain CD45^int^CX3CR1+CD11b+ microglia. Gating used for both ManCou14 and efferocytosis experiments requiring flow cytometry. **(c)** Normal expression of CD11b and CX3CR1 by WT and KO microglia. Each dot represents one mouse from the experiment in Fig. 2c, and the gMFI is plotted for each sample, with n = 4 mice per condition. Data are shown as mean ± SEM. Significance was determined using a two-tailed, unpaired t-test. ns = not significant. **(d-f)** Docking analysis of fructofuranose, the cyclic structure of fructose, and ManCou14 (Mancou-Pyr). **(d-e) (d)** Docking diagram projection of the major binding interactions of fructofuranose with Glut5_out_ and graphical summary of number of interactions per position in the docked site. (**e**) Diagram projection of the major binding interactions of ManCou14 with Glut5out and graphical summary of number of interactions per position in the docked site. (**f**) Overlay of fructofuranose (yellow) and ManCou14 (gray) in the binding site of GLUT5. Molecular Operating Environment (MOE) was used to perform docking and generate images. Energy-refined structures for both fructofuranose and ManCou14 were employed to generate docking poses, which were further refined using a simple scoring function. During the docking process, the GLUT5 structure was kept rigid. For the analysis in (d-f), we evaluated GLUT5 activity in microglia by using GLUT5-specific fluorescent molecular probes. We utilized coumarin conjugates of 2-amino-2,5-anhydro-D-mannitol (ManCous) as high-affinity, GLUT5-specific substrates (Begoyan et al., Chem. Commun., 2018). The fructose-like structure of 2-amino-2,5-anhydro-D-mannitol facilitates key interactions within the active site of the transporter, promoting the effective uptake of these probes. Additionally, the presence of coumarin enhances the GLUT5-ManCou binding (Ainsley et al., Chem. Commun., 2018). Variations in coumarin substitution at the C4 position supported tuning of binding affinity, resulting in analogs with over 200-fold higher affinity for GLUT5 compared to fructose (with a Km for fructose of approximately 11 mM) (Begoyan et al., Chem. Commun., 2018). As a result, ManCous competes with fructose for GLUT5 at physiological concentrations and effectively translocates through the transporter, enabling fluorescence-based monitoring of GLUT5 activity in live cells (Kannan et al., Biosensors, 2018). The ability of ManCous to bind and pass through GLUT5 was validated through previous inhibition studies using competitive and non-competitive substrates for GLUT5 and other GLUTs, as well as through modeling studies of GLUT5-probe complexes. Specifically for the probe used in our study (ManCou14), the overlaid docked structures with fructose within GLUT5 demonstrate both substrates to be positioned within the fructose-binding site, facilitating hydrogen bonding interactions with key residues essential for fructose recognition and uptake via GLUT5 (Gln288, Ser143, Ser392, Gln167) (Nomura et al., Nature, 2015). In addition to the sugar-binding site, ManCou14 interacts with His387— a residue identified as contributing to substrate selection by GLUT5 (Nomura et al., Nature, 2015). The greater number of hydrogen bonding and secondary interactions explains the high affinity of ManCou14, while interaction with specific residues supports the specific recognition by GLUT5.

**Extended Data Fig. 9:**
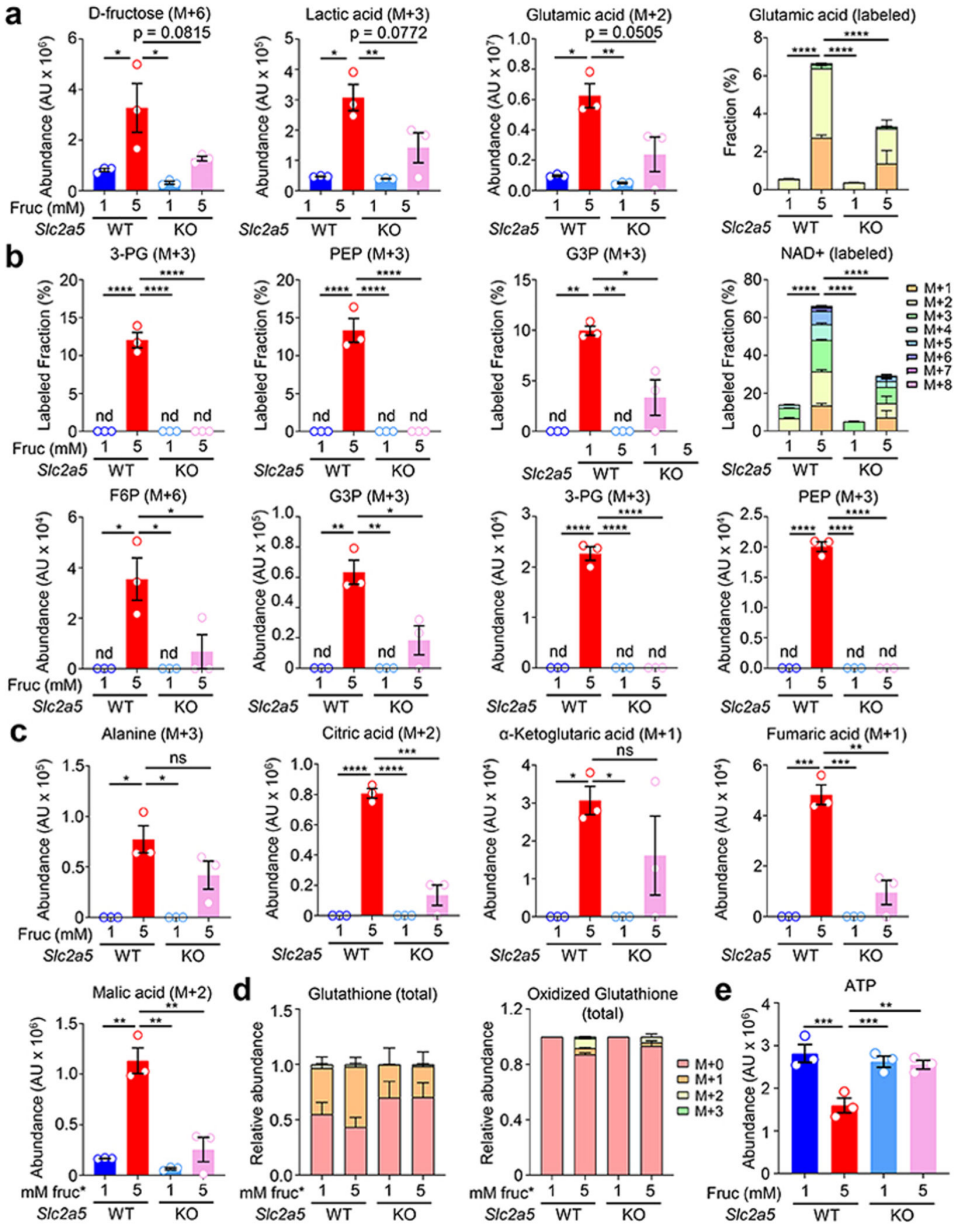
Additional analyses of 13C-labeled metabolites in primary microglia *in vitro*. **(a)** Relative abundance of indicated fructolysis metabolites. All data from **(a-e)** was obtained from experiments detailed in Fig. 3d and Extended Data Fig. 9 and are shown as mean ± SEM. Significance was determined using two-way ANOVA with Tukey’s multiple comparisons test. *p < .05, **p < .01, ****p < .0001. D-fructose, lactic acid, and glutamic acid (fractional enrichment far right) are shown. **(b)** Fractional enrichment and relative abundance of additional intermediates. Data are shown as mean ± SEM. Significance was determined using two-way ANOVA with Tukey’s multiple comparisons test. *p < .05, **p < .01, ****p < .0001. Fructose 6-phosphate (F6P), glyceraldehyde 3-phosphate (G3P), 3-phosphoglyceric acid (3-PG), phosphoenolpyruvic acid (PEP), and nicotinamide adenine dinucleotide (NAD+) are shown. **(c)** Relative abundance of alanine and TCA cycle intermediate downstream of fructolysis. Data are shown as mean ± SEM. Significance was determined using two-way ANOVA with Tukey’s multiple comparisons test. ns = not significant, *p < .05, **p < .01, ***p < .001, ****p < .0001. Alanine, citric acid, α-ketoglutaric acid, fumaric acid, and malic acid are shown. **(d)** Relative abundance of Glutathione and Oxidized Glutathione. Data are shown as mean ± SEM. **(e)** Relative abundance of ATP. Data are shown as mean ± SEM. Significance was determined using two-way ANOVA with Tukey’s multiple comparisons test. **p < .01, ***p < .001.

We next sought to determine if microglia specifically internalize and catabolize fructose and how high fructose exposure affects microglia metabolism. To this end, we took two orthogonal approaches. First, we used a novel 2,5-anhydro-D-mannitol-coumarin based GLUT5-specific probe (ManCou14)^36^ to assess cell type-specific GLUT5 activity in the brain (Extended Data Fig. 8a-f). Consistent with the restricted expression of GLUT5 by microglia, we found that microglia, but not CD45- or other CD45+ cells, exhibited ManCou14 fluorescent signal which was significantly reduced in microglia lacking GLUT5 (Fig. 3c). Second, we performed tracing of uniformly-labeled ^13^C [U-^13^C]-fructose in wildtype and GLUT5-deficient primary microglia that were first conditioned in either low (1 mM) or high (5 mM) fructose in the presence of physiological glucose and oxygen then pulsed with a fixed concentration of labeled fructose. Wildtype microglia conditioned in either low or high fructose were capable of consuming and catabolizing fructose, demonstrated by ^13^C-labeled lactic acid (M+3) and glutamic acid (M+2; Fig. 3d; Extended Data Fig. 9a). However, consistent with our observation that microglia upregulate GLUT5 in response to high fructose, we observed that high fructose-conditioned microglia consumed more fructose (M+6 fructose; Extended Data Fig. 9a) and catabolized significantly more fructose as indicated by increased fractional enrichment and relative abundance of ^13^C-labeled lactic acid and glutamic acid (Fig. 3d; Extended Data Fig. 9a). We also found that high fructose-conditioned microglia exhibited significant increases in both relative abundance and fractional enrichment of fructose catabolism intermediates (Fig. 3d; Extended Data Fig. 9b). This difference was particularly striking for the hexokinase (HK) product fructose 6-phosphate (F6P; Fig. 3d; Extended Data Fig. 9b).

Although our data to this point suggest that fructose transport via GLUT5 is necessary for the detrimental effects of high fructose on microglia phagocytosis, it remains possible that the rescue observed in GLUT5-deficient microglia is independent of the metabolic changes observed in high fructose-treated microglia. However, consistent with our hypothesis that high fructose-induced alterations of microglia metabolism result in decreased phagocytosis capacity, we found that fructose uptake and catabolism in high fructose-treated, GLUT5-deficient microglia were reverted to levels near that of microglia conditioned in low fructose (Fig. 3d; Extended Data Fig. 9a). Additionally, the striking shift towards HK-mediated fructolysis observed in high fructose-conditioned microglia was almost completely reversed in high fructose-treated, GLUT5-deficient microglia (Fig. 3d; Extended Data Fig. 9b).

A recent study demonstrated that deletion or inhibition of the HK isoform HK2 resulted in increased microglia phagocytosis, whereas enforcing HK2 activity resulted in defective microglia phagocytosis, in part because of decreased ATP production^37^. Furthermore, another recent study found that deletion of translocator protein (TSPO) from microglia led to increased HK2 localization and binding to mitochondria, which similarly decreased ATP production and phagocytic activity^38^. Interestingly, we found that high fructose-conditioned microglia had increased fractional enrichment and relative abundance of fructose-derived NAD+ (Extended Data Fig. 9b) and increased contribution of fructose carbons into the TCA cycle (Extended Data Fig. 9c,d). Strikingly, we also observed significantly less total ATP in high fructose-conditioned microglia (Extended Data Fig. 9e). Importantly, the increased contribution of fructose to the TCA cycle and decreased total ATP observed in high fructose-treated microglia was significantly reversed in GLUT5-deficient microglia (Fig. 3d; Extended Data Fig. 9a-e).

**Extended Data Fig. 10:**
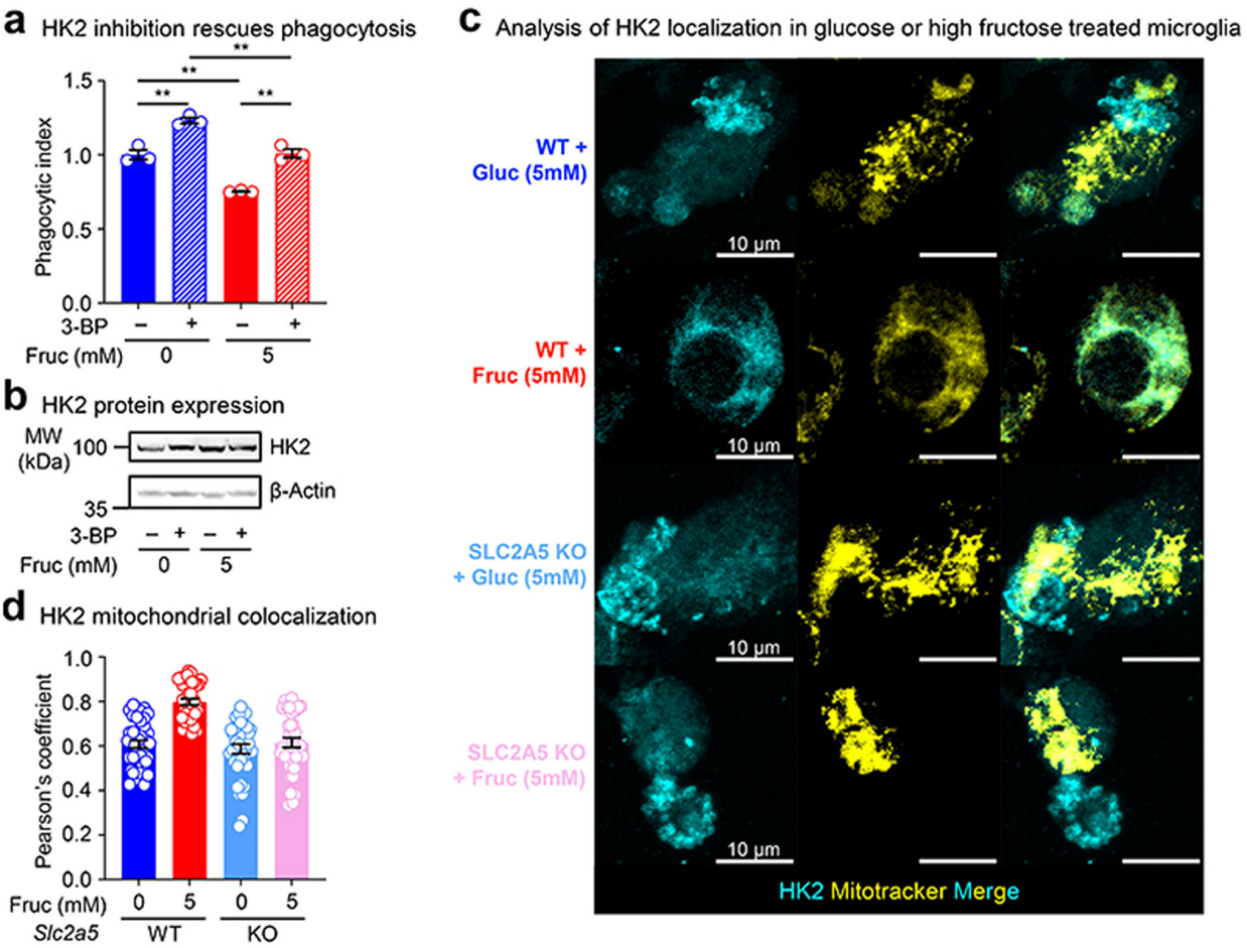
Determination of the HK2 relevance for microglia phagocytosis. **(a)** Inhibition of HK2 activity rescues high fructose-induced suppression of microglia phagocytic activity. Microglia were plated and conditioned as in Fig. 2c, but with the inclusion of the HK2-specific inhibitor 3-BP. Conditioned microglia were cultured with CypHer5E-labeled apoptotic neurons for 1 h at a 0.5:1 target-to-phagocyte ratio, then isolated and analyzed for phagocytosis via flow cytometry. Shown is the phagocytic index (percent phagocytosis in experimental microglia divided by percent phagocytosis in control microglia) calculated from three independent experiments. Data are shown as mean ± SEM. Significant was calculated by two-way ANOVA with Tukey’s multiple comparisons test. **p < .01. **(b)** High fructose enhances HK2 expression. Microglia were treated identically to cells in **(a)**, but pooled from multiple independent wells for western blot analysis. Data is representative of two independent experiments. For gel source data, see Supplementary Figure 1. **(c, d)** High fructose increases mitochondrial HK2 colocalization in a GLUT5-dependent manner. Primary microglia from WT and KO mice were cultured with 5 mM glucose or 5 mM glucose and 5 mM fructose with mannitol as a control for osmolarity. Mitotracker Red CMXRos-labeled microglia were cultured with CypHer5E-labeled apoptotic neurons at a 1:1 ratio for 30 min, then fixed and analyzed via confocal microscopy. **(c)** Representative images were taken using confocal microscopy (63x oil immersion objective, 7 μm thick image stacks with 0.5 μm step-size), and max-intensity z-projections were used to analyze colocalization between HK2 and Mitotracker. **(d)** Pearson’s coefficients were calculated in Fiji, with 1 denoting perfect colocalization and 0 denoting no colocalization. Data shown is representative of two independent experiments, with *n* = 37 to 40 cells per condition. Scale bar, 10μm.

Lastly, in lieu of our findings and previous reports that suggest that HK2 localization and activity suppress microglia phagocytosis, we tested the hypothesis that HK2 mitochondrial localization and activity underlies high fructose-mediated suppression of microglia phagocytosis. Indeed, blocking HK2 activity using the isoform-specific inhibitor 3-BP significantly boosted microglia phagocytosis in glucose-only conditions and, importantly, rescued microglia phagocytosis capacity under high fructose conditions (Extended Data Fig. 10a). Furthermore, we found that high fructose conditioning significantly increases mitochondrial localization of HK2 that was reversed in microglia lacking GLUT5 (Extended Data Fig. 10b-d). Collectively, our data suggest that high fructose exposure, via GLUT5-dependent transport and fructose catabolism, alters microglia metabolism towards a non-phagocytic metabolic state in part mediated through increased HK2 mitochondrial localization and activity.

### Early life high fructose exposure contributes to cognitive defects and the development of anxiety-like behavior

**Extended Data Fig. 11:**
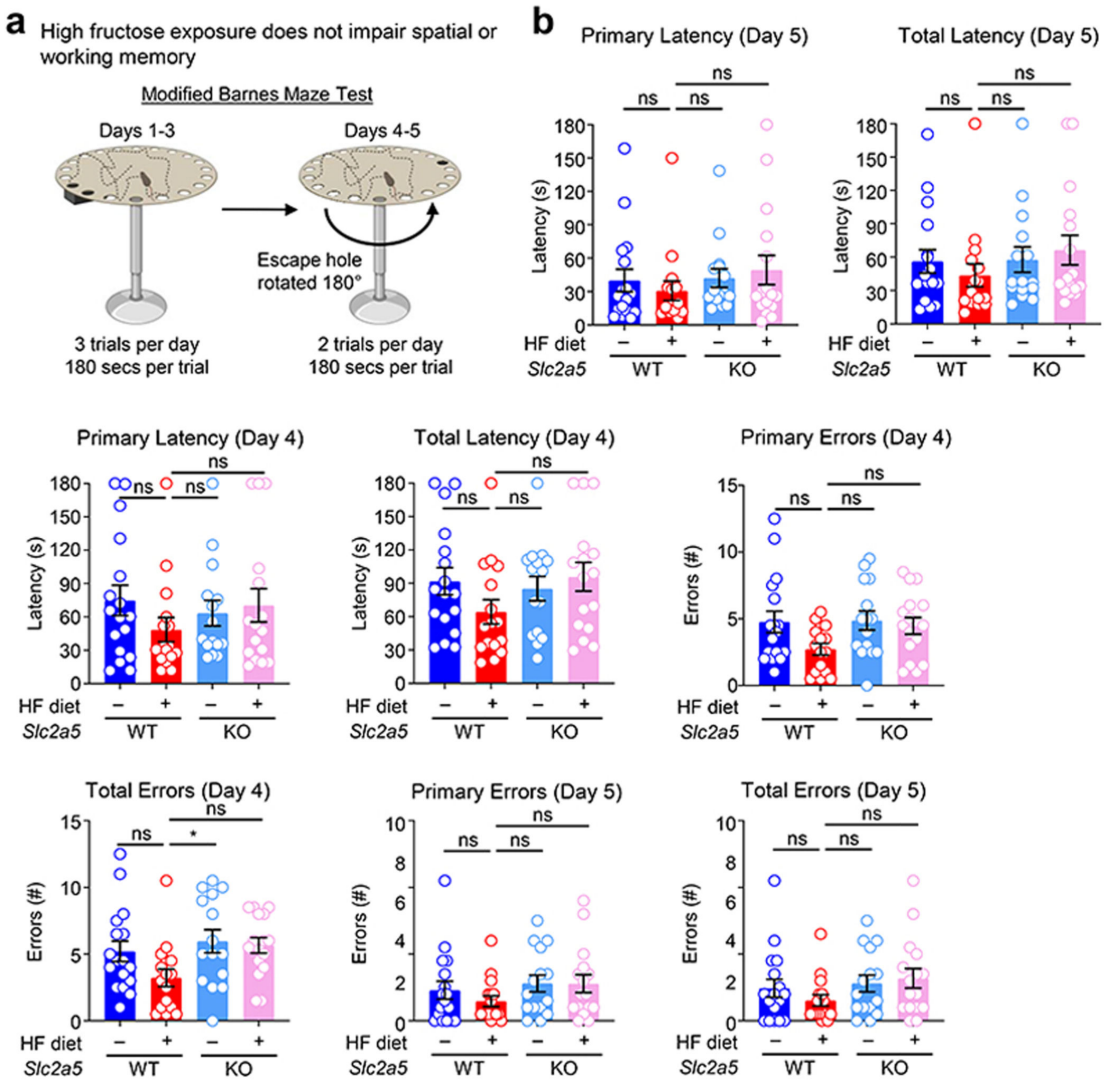
Analysis of Barnes Maze results. **(a, b)** Evaluation of spatial learning and memory via the modified Barnes Maze. Experiments were performed as in Fig. 4 but used to assess spatial learning and memory. **(a)** Schematic of experiments used to analyze spatial learning and memory using the modified Barnes Maze. After three training days during which the escape hole was placed at the same location for three trials each day, the escape hole was then moved 180° on days 4 and 5 and two trials per day were performed. **(b)** Each of the four cohorts were tested as outlined in **(a)**, with the numbers of mice at *N* = 17 for WT CD, *N* = 16 for WT HF, *N* = 15 for KO CD, and *N* = 16 for KO HF. Data are shown as mean ± SEM. Significance was determined using two-way ANOVA with Tukey’s multiple comparisons test. ns = not significant.

**Fig. 4:**
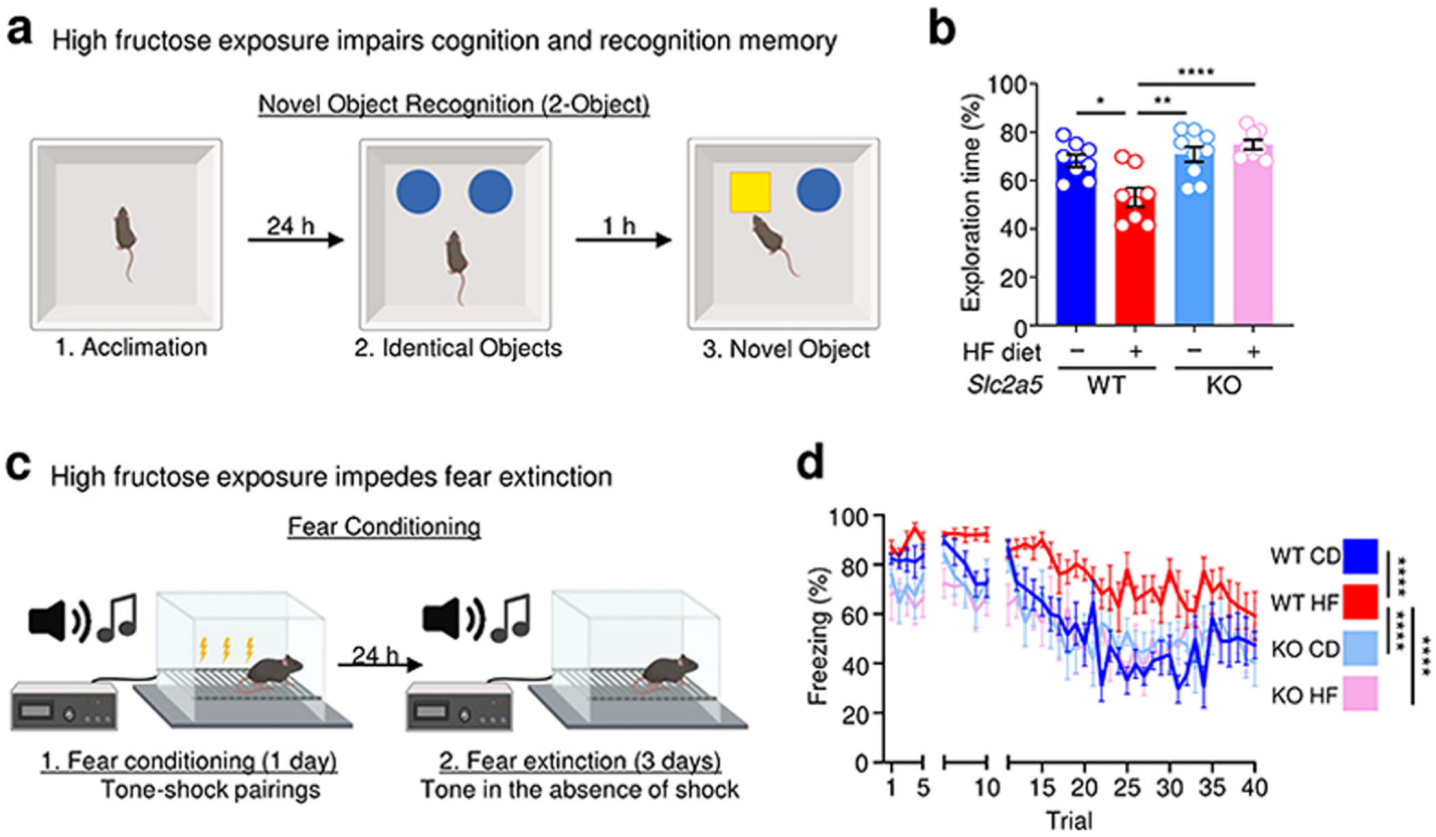
Early-life high fructose exposure impairs adolescent learning and cognition. **(a, b)** (**a**) Schematic of the novel object recognition (NOR, 2-object) task used to measure animal cognition and recognition memory. **(b)** Offspring from wildtype (WT) and *Slc2a5*^–/–^ (KO) mice were raised by dams on control diet (CD) or high fructose (HF) diet until weaning. Weaned juveniles (age range: P26-P34) were subsequently tested as described in **(a)**, with the numbers of mice at *N*=8 for WT CD, *N*=8 for WT HF, *N*=9 for KO CD, and *N*=8 for KO HF. Data shown as mean±SEM. Significance was determined using two-way ANOVA with Tukey’s post-hoc. *p<.05, **p<.01, ****p<.0001. **(c, d)** Experiments were performed as in **(b)** but used to assess fear extinction. **(c)** Schematic of experiments used to analyze fear extinction. **(d)** Each of the four cohorts in were tested as outlined in **(c)**. Shown is the percentage of time mice freeze, with *N*=8 mice per condition. Data shown as mean±SEM. Significance was determined using two-way ANOVA with Tukey’s post-hoc on AUCs. ****p<.0001.

Given the importance of microglial phagocytosis in the first week of life, we sought to determine whether excess early life fructose exposure directly causes cognitive defects, and whether those impairments were dependent on GLUT5. To address this, wildtype or GLUT5-deficient mice were raised by dams on control diet (CD) or high fructose (HF) diet until weaning, then subjected to three key behavioral assessments previously shown to be affected by perturbed microglial phagocytosis: novel object recognition, modified Barnes maze, and fear extinction^39–41^. First, we performed novel object recognition (NOR; Fig. 4a), which is an experiment that broadly assesses animal cognition and recognition memory by applying the principle that mice prefer novel objects over objects previously introduced. We found that adolescent mice born to and nursed by dams on HF diet exhibited no preference for the novel object (~50%) compared to adolescent mice born to and nursed by dams on CD (~75%, Fig. 4b). On the other hand, the loss of novel object preference observed in adolescent mice born to and nursed by dams on HF diet was completely reversed in GLUT5-deficient adolescent mice (Fig. 4b). Second, we performed the modified Barnes maze task (Extended Data Fig. 11a), which specifically assesses spatial and working memory. In contrast to our results with the NOR task, adolescent mice born to and nursed by dams on HF did not exhibit a significant defect in any parameters assessed, including the time to locate the escape hole (primary latency; Extended Data Fig. 11b), time to enter the escape hole (total latency; Extended Data Fig. 11b), number of holes incorrectly checked before locating the correct one (primary errors; Extended Data Fig. 11b), and number of holes incorrectly checked before entering the correct one (Extended Data Fig. 11b). Instead, adolescent mice born to and nursed by dams on HF diet displayed modestly better performance across some metrics that was reversed in GLUT5-deficient adolescent mice (Extended Data Fig. 11b). The contradiction between NOR and Barnes maze performance suggests that HF diet affects cognition (assessed by NOR) and not memory (assessed by both NOR and Barnes maze). Finally, we performed fear extinction (Fig. 4c), which is an associative learning task that tests both the acquisition of fear through Pavlovian learning (conditioning) and the subsequent reversal of that acquired fear (extinction). This test is particularly important because deficits in fear extinction after an environmental threat has passed have been implicated in various anxiety disorders including PTSD^42^. Consistent with our observation that adolescent mice born to and nursed by dams on HF do not have altered memory, an equivalent number of adolescent of mice acquired a fear response across conditions (Fig. 4d). Strikingly, however, adolescent mice born to and nursed by dams on HF exhibited drastically impaired fear extinction compared to adolescent mice born to and nursed by dams on control diet (Fig. 4d). Importantly, the deficits in fear extinction observed in adolescent mice born to and nursed by dams on HF diet were completely reversed in GLUT5-deficient adolescent mice (Fig. 4d). Thus, exposure to fructose in early life contributes to cognitive defects and the development of anxiety-like behavior in adolescent mice that is rescued by global GLUT5 deficiency.

### Effects of early life high fructose exposure *in vivo* are myeloid-specific

**Extended Data Fig. 12:**
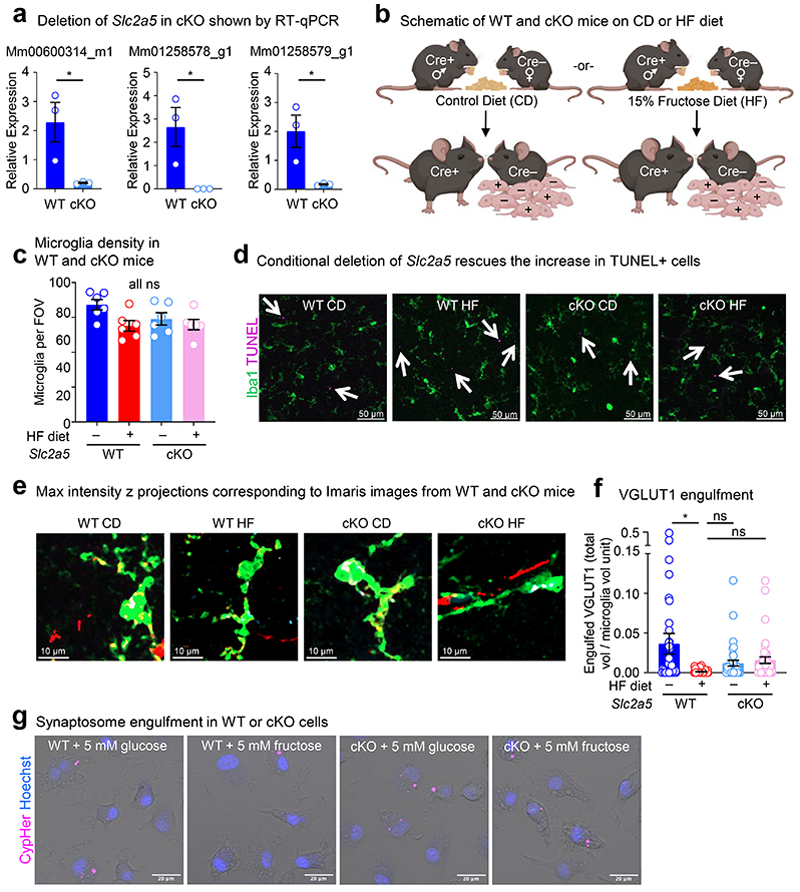
Analysis of microglia phagocytosis in mice with myeloid-specific deletion of *Slc2a5*. **(a)** Absence of *Slc2a5* in murine microglia from conditional knockout mice confirmed by RT-qPCR. Three TaqMan probes were tested using P2-P4 neonatal microglia isolated from WT and cKO mice. Each dot represents one mouse. Data are shown as mean ± SEM. Significance was determined using a two-tailed, unpaired t-test. *p < .05. **(b)** Schematic of WT (cre-negative) and cKO (cre-positive) mice used in diet experiments. 8–10-week-old Cre-negative females were paired with cre-positive males, and placed on either CD or HF. For *in vitro* experiments, P2-P4 neonatal mice were genotyped immediately before microglia isolation to enable pooling. **(c)** Microglia density in WT and cKO mice. Quantitation of microglia (Iba1+) numbers in the prefrontal cortex from P7 WT and cKO mice (littermates) exposed to CD or HF. Confocal microscopy (20x objective, 20 μm thick image stacks with 2 μm step-size) was used to obtain imaging data. Number of microglia were counted in four FOVs (fields of view) across two tissue sections per mouse for each condition in Fiji, and each dot represents the mean for one animal, with *N* = 6 mice per condition. Data are shown as mean ± SEM. Significance was determined using two-way ANOVA with Tukey’s multiple comparisons test. ns = not significant. **(d)** Representative images from WT and cKO mice corresponding to quantitation of TUNEL in Fig. 5a. Scale bar, 50 μm. **(e)** Max intensity z-projections of cells used for Imaris 3D reconstruction in Fig. 5b. Scale bar, 10 μm. **(f)** High fructose diet does not significantly affect engulfment of VGLUT1 material. Quantitation of engulfed VGLUT1 material in WT and cKO cells in Fig. 5b,c, with normalization Iba+ cell volume using Imaris. Data points represent individual cells with *N*=8 WT CD, *N*=8 WT HF, *N*=8 cKO CD, and *N*=7 cKO HF mice, with points representing individual cells (*n* = 44, 37, 39, and 39, respectively). Data are shown as mean ± SEM. Significance was determined using two-way ANOVA with Tukey’s multiple comparisons test. ns = not significant, *p < .05. **(g)** Representative images of in vitro phagocytosis corresponding to Fig. 5b. Scale bar, 20 μm.

**Extended Data Fig. 13:**
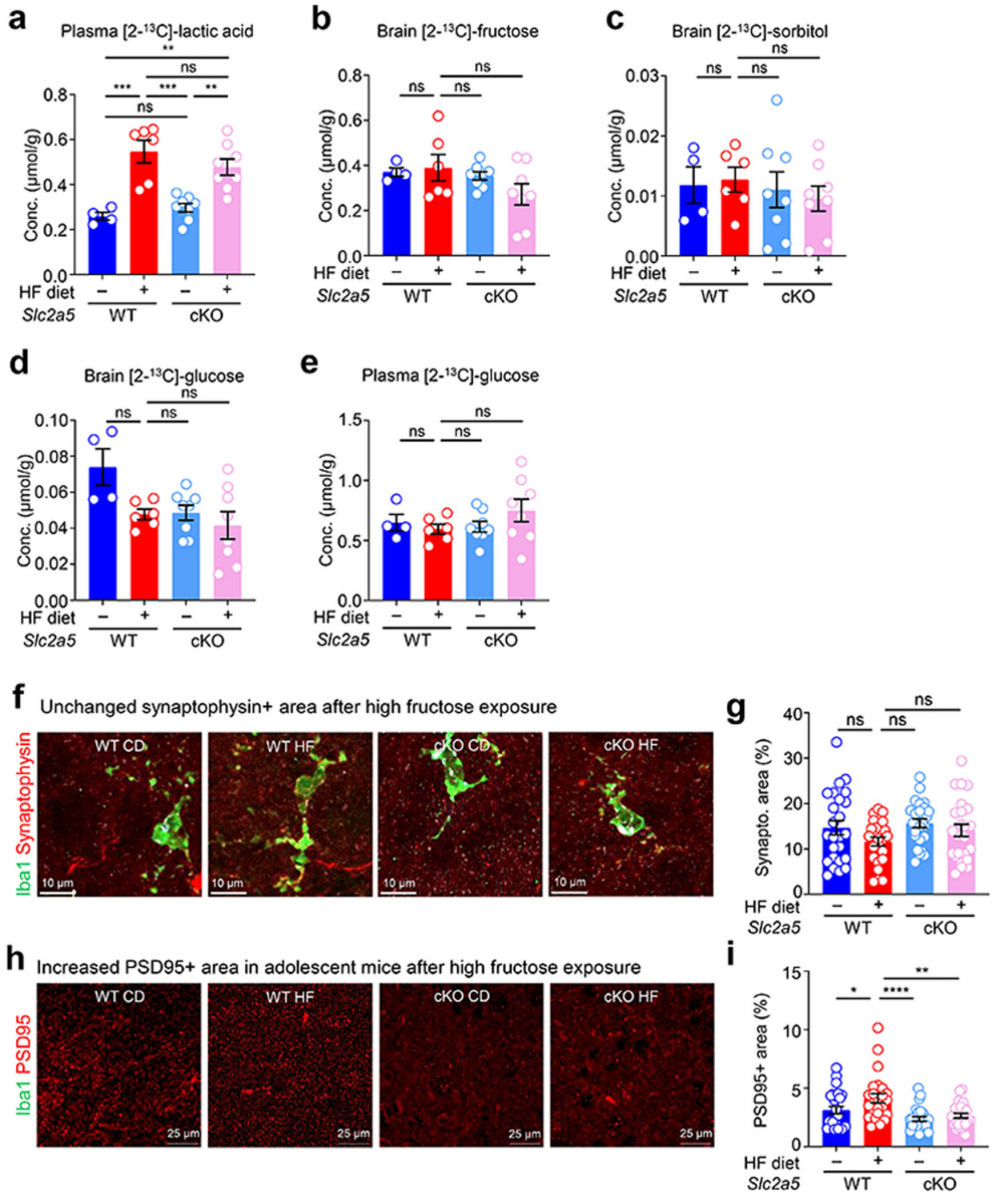
Analysis of behavior in mice with myeloid-specific deletion of *Slc2a5*. **(a-e)** Additional NMR measurements from the brains of WT and cKO mice corresponding to Fig. 5e. Quantitation of labeled plasma lactic acid (a), brain fructose (b), brain sorbitol (c), brain glucose (d), and plasma glucose (e) are shown, with *N* = 4 for WT CD, *N* = 6 for WT HF, *N* = 8 for cKO CD, and *N* = 8 for cKO HF. Data are shown as mean ± SEM. Significance was determined using two-way ANOVA with Tukey’s multiple comparisons test. ns = not significant, **p < .01, ***p < .001. **(f, g)** High fructose does not alter synaptophysin+ area after fear extinction. Representative images (**f**) and quantitation (**g**) of synaptophysin+ area corresponding to Fig. 5h,i. Quantitation was performed as described in Chu et al. 2019, with each dot representing one FOV and five FOVs per mouse, with *N* = 5 mice per condition and 25 FOVs per mouse. Data are shown as mean ± SEM. Significance was determined using two-way ANOVA with Tukey’s multiple comparisons test. ns = not significant. Scale bar, 25 μm. **(h, i)** High fructose exposure enhances PSD-95+ area immediately after fear extinction experiments. Brains from juvenile mice were immediately collected after fear extinction experiments in **(g)**. Confocal microscopy of the prefrontal cortex was performed using a 63x oil immersion objective (5μm thick image stacks with step-size of 0.5μm) and analyzed using Fiji. **(h)** Representative images of PSD-95+ area are shown, with representative images of synaptophysin area from the same brains are shown in Extended Data Fig. 13f. **(i)** Quantitation was performed as described in Chu et al. 2019, with each dot representing one FOV and five FOVs per mouse, with N = 5 mice per condition and 25 FOVs per mouse. Data are shown as mean ± SEM. Significance was determined using two-way ANOVA with Tukey’s multiple comparisons test. *p < .05, **p < .01, ****p < .0001. Scale bar, 25μm.

**Fig. 5:**
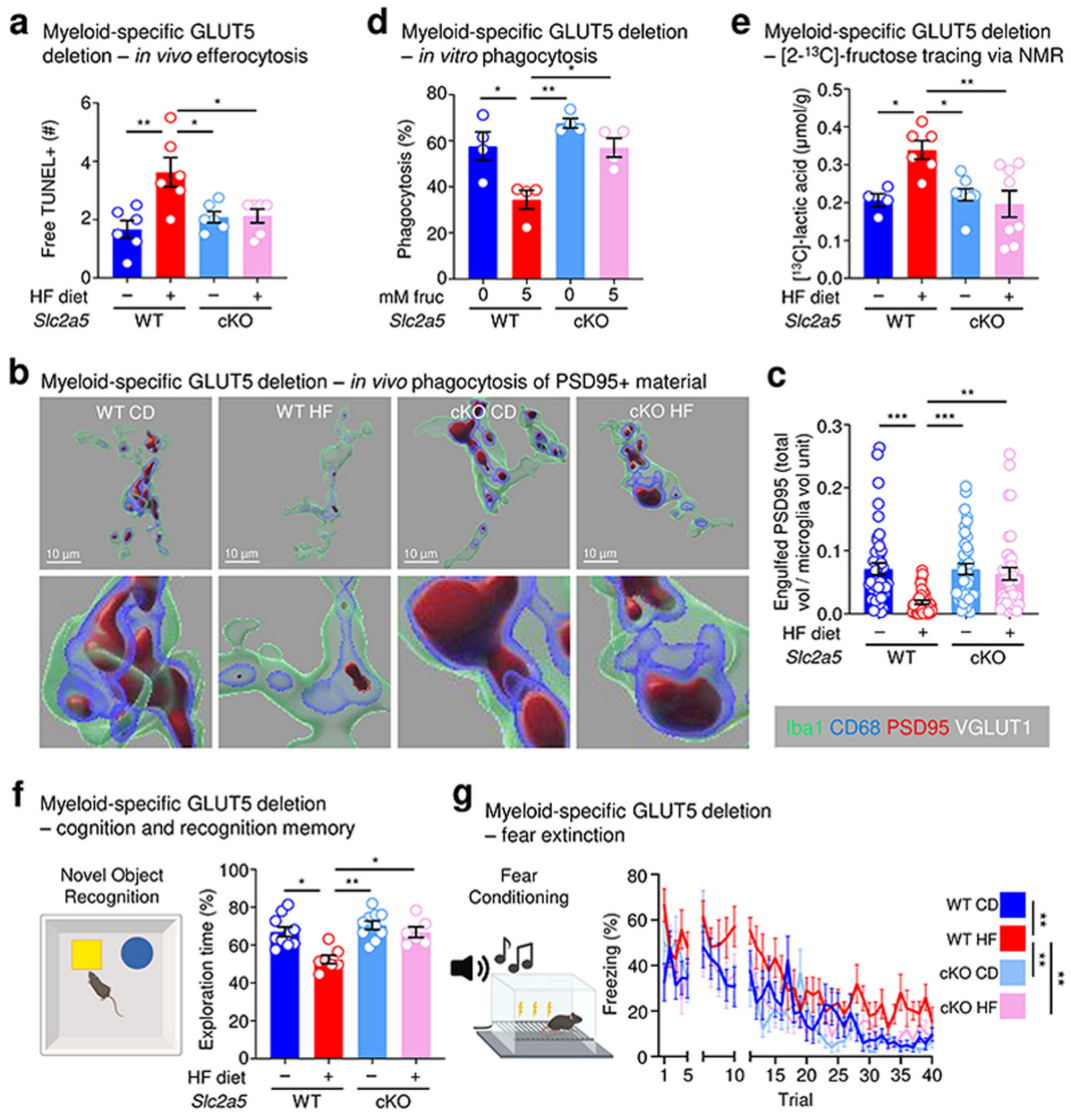
High fructose-dependent deficits in microglia function are cell-intrinsic **(a)** Quantitation of uncleared (free) TUNEL+ puncta in the prefrontal cortex (PFC) of P7 wildtype (WT) or CSF1R^Cre^ x *Slc2a5*^fl/fl^ (cKO) mice exposed to CD or HF. Data are from four FOVs across two tissue sections per mouse/condition. Each point represents the mean for one mouse, with *N*=6 mice per condition. **(b, c)** 3D reconstruction **(b)** and quantitation **(c)** of representative Iba1+ microglia containing PSD-95 puncta within CD68+ phagolysosomes in the PFC of P7 WT and cKO mice exposed to CD or HF. Data points represent individual cells with *N*=8 WT CD, *N*=8 WT HF, *N*=8 cKO CD, and *N*=7 cKO HF mice, with points representing individual cells (*n* = 44, 37, 39, and 39, respectively). Scale bar, 10μm with 4x digital zoom. **(d)** Analysis of WT and cKO microglia phagocytosis as in Fig. 2d. Data represents three independent experiments (four FOVs per condition). **(e)** Quantitation of [2-^13^C]-lactic acid in brains of WT and cKO P7 mice as in Fig. 3a, with *N*=4 WT CD, *N*=6 WT HF, *N*=8 for KO CD, and *N*=8 KO HF mice. **(f)** WT and cKO mice were tested for novel object recognition as in Fig. 4a, with *N*=10 WT CD, *N*=7 WT HF, *N*=10 cKO CD, and *N*=6 cKO HF mice. **(g)** Adolescent WT and cKO mice were tested for fear extinction as in Fig. 4e, with *N*=9 WT CD, *N*=10 WT HF, *N*=10 cKO CD, and *N*=8 cKO HF mice. Data are shown as mean±SEM. Significance was determined using two-way ANOVA with Tukey’s post-hoc. For (**g**), statistical analysis was performed on AUCs. *p<.05, **p<.01, ***p<.001.

Although we demonstrate that the effect of early life fructose exposure on neonatal microglia function and neurodevelopment could be reversed in neonates lacking GLUT5 globally, it remains unclear whether this effect is direct or indirect *in vivo*. To determine specificity, we generated *Slc2a5*^fl/fl^ mice and bred them to a Cre (Csf1r^Cre^) that allows us to delete GLUT5 from myeloid cells as early as embryonic day (E)6.5 (Extended Data Fig. 12a). Using these mice, we placed dams on isocaloric diets containing either 0 kcal% sucrose/0 kcal% fructose (control diet, CD) or 15 kcal% high fructose (HF) diet, then analyzed resultant offspring (Extended Data Fig. 12b). Importantly, myeloid-specific deletion of GLUT5 completely reversed the HF diet-induced microglia phagocytosis defect, but not microglia numbers, observed *in vivo* (Fig. 5a-c; Extended Data Fig. 12d-f) and the high fructose conditioning-induced suppression of microglia phagocytosis *in vitro* (Fig. 5d; Extended Data Fig. 12g). Additionally, myeloid-specific GLUT5 deletion reversed the increased fructose catabolism observed in brains from neonates born to and nursed by dams on HF diet (Fig. 5e; Extended Data Fig. 13a-e). Interestingly, this increased fructose catabolism is likely not due to polyol activity, as labeled plasma lactic acid levels, unlike in the brain, remained elevated in mice with myeloid-specific GLUT5 deletion (Extended Data Fig. 13a) nor did we detect the presence of labeled sorbitol or differences in labeled glucose (Extended Data Fig. 13b-e).

We next sought to determine if the effect of high fructose on neurodevelopment depends on microglial GLUT5. To test this, wildtype or myeloid-specific GLUT5-deficient mice were raised by dams on CD or HF diet until weaning, and we then analyzed adolescent mice for novel object recognition (NOR) and fear extinction. Strikingly, similar to adolescent mice globally lacking GLUT5, adolescent mice with myeloid-specific GLUT5 deficiency recovered preference for the novel object lost in adolescent mice born to and nursed by dams on HF diet (Fig. 5f). Additionally, the deficit in fear extinction observed in adolescent mice born to and nursed by dams on HF diet was reversed in adolescent mice with myeloid-specific GLUT5 deficiency (Fig. 5g). The defects in NOR and fear extinction observed in adolescent mice born to and nursed by dams on HF diet were concomitant to increased total PSD-95+ area relative to total synaptophysin+ area (Extended Data Fig. 13f-i), a measure of extinction-related neuron dendritic spine remodeling^17^. Consistently, the increased total PSD-95+ area in adolescent mice born to and nursed by dams on HF diet was reversed in adolescent mice with myeloid-specific GLUT5 deficiency (Extended Data Fig. 13f-i). Collectively, our data suggest that the microglia phagocytosis defect and the development of anxiety-like behavior that occurs in mice exposed to high fructose in early life is due to microglial GLUT5-dependent fructose metabolism.

## Discussion

The chronic excessive consumption of fructose has contributed to a major public health crisis because of its contribution to the increased prevalence of metabolic disease. Recent epidemiological evidence suggests that the negative effects of high fructose diet may extend beyond metabolic disease and be associated with the onset of mood and anxiety disorders especially in adolescents. Here, we find that high fructose diet, delivered either during pregnancy or directly to early perinatal mice, dramatically decreases microglia phagocytic activity Importantly, we show that deletion of the high affinity fructose transporter SLC2A5 (GLUT5) in neonates both globally and specifically in microglia completely reverses microglia phagocytic dysfunction. We found that high fructose conditioning of both mouse and human microglia *in vitro* directly suppresses phagocytosis capacity which was reversed in GLUT5-deficient mouse microglia. We also found that high fructose exposure drives increased GLUT5 dependent fructose uptake and catabolism into fructose 6-phosphate, rewiring microglia metabolism towards a hypo-phagocytic state in part by increasing the mitochondrial localization and activity of the enzyme hexokinase 2. Importantly, mice exposed to high fructose as neonates develop anxiety-like behavior as adolescents which was rescued in GLUT5-deficient animals. Taken together, our work provides a mechanistic explanation for the epidemiological observation that exposure to high fructose early in life is associated with increased anxiety disorder prevalence in adolescents.

Our work revealed that exposing microglia to high fructose drives the catabolism of fructose into fructose 6-phosphate. Fructose is known to be catabolized via two different enzymes: ketohexokinase into fructose 1-phosphate, which is dominant in the liver and the intestine, or hexokinase into fructose 6-phosphate. Interestingly, two recent studies found that hexokinase 2 (HK2), the main isoform in microglia, is a regulator of microglia phagocytosis^37, 43^. Specifically, Leng et al. demonstrated that deletion or pharmacological inhibition of HK2 caused increased microglia phagocytosis. On the other hand, supplementation with glucose 6-phosphate or fructose 6-phosphate, but not fructose 1,6-bisphosphate, suppressed microglia phagocytosis activity^37^. Additionally, a recent study^38^ found that deletion of TSPO, which increases localization of HK2 to the mitochondria, also significantly decreased microglia phagocytosis. These findings suggest that HK2 mitochondria localization and enzymatic activity directly impedes microglial phagocytosis, consistent with our finding that microglia exposed to HF display increased synthesis of fructose 6-phosphate from fructose and increased mitochondrial localization concomitant with decreased phagocytosis capacity which could be reversed by inhibiting HK2 activity. Our findings, and the previous work on HK2, are particularly interesting when considering that mitochondrial metabolic activity is enhanced in early neonatal mice (P9) and is required for microglia phagocytosis, but subsides after the critical microglia phagocytic window^44^. Future work will be required to determine how hexose metabolism, HK2 enzymatic activity, the fructolysis enzyme KHK, and mitochondrial metabolism specifically function to support or suppress microglia phagocytosis across development into adulthood.

Although microglia uniquely express SLC2A5 (GLUT5), its expression increases in both mouse and human microglia during aging. It remains unclear what, if any, homeostatic function GLUT5/fructose transport and metabolism serve in adult microglia. Here, we show that neonatal microglia use GLUT5 to import and metabolize fructose, but doing so suppresses capacity to perform phagocytic functions essential during development. Given that microglia transition from a highly phagocytic state during the critical neonatal window to a quiescent, non-phagocytic state in adulthood, we speculate that GLUT5 upregulation, and concomitant increased fructose import/catabolism, is determinative of this transition and supports microglial longevity. This hypothesis is supported by multiple observations. For instance, microglia are metabolically flexible, capable of surviving in an environment that preferentially delivers glucose to astrocytes and neurons^45^. Additionally, microglia reside in physiologically low oxygen often for the entire life of the host. We and others recently reported that peripheral tissue-resident macrophages residing in physiological hypoxia downregulate total glucose consumption and catabolism, instead efficiently shunting glucose into a noncanonical pentose phosphate pathway loop that supports redox homeostasis^46, 47^. Whether increased GLUT5 expression allows microglia to better use the ~200 mM fructose present in the human adult CNS^48^ and if this goes awry in unhealthy aging (e.g., neurodegeneration) remains unknown. Interestingly, a recent study of CNS resident myeloid subsets found that Kolmer’s epiplexus cells^49^, Sall1-dependent microglia-like cells residing at the apical surface of the choroid plexus, also express GLUT5 concomitant with direct exposure to the fructose-abundant cerebrospinal fluid. Here, we found that high fructose exposure induces increased GLUT5 mRNA and protein in mouse and human microglia. Consistent with this, a previous study found that the microglia lineage-determining transcription factor Sall1 is active at the enhancer of *Slc2a5*/*SLC2A5 in vivo*^50^, suggesting that exposure to prolonged fructose, either in the CNS parenchyma or at the choroid plexus, is necessary for adult microglia lineage stability and quiescence. How fructose enters the brain remains controversial^23^, several previous studies have shown that fructose is significantly higher in the cerebrospinal fluid than in the serum of adults^48, 51, 52^. Thus, understanding how adult microglia use GLUT5 and fructose in health and disease will be an important area of work moving forward, particularly given the prevalent use of high fructose corn syrup as an additive in the common Western diet.

Finally, our results have potentially profound implications not only for pregnancy but also adolescent development. In humans, microglial phagocytosis and neurodevelopment continues well into the second decade of life^53, 54^ potentially broadening the window when high fructose consumption could be detrimental. High fructose consumption itself likely does not cause the development of mood or anxiety disorders. However, it is plausible that high fructose consumption serves as the first ‘hit’, while a subsequent trauma is required to trigger the development of a disorder^55–57^. Although work from several groups has demonstrated that intestinal epithelial cells and the liver primarily metabolize dietary fructose, it was also shown that excess fructose can overwhelm this “shield”, particularly when consumed in liquid form^35^. This is especially important when considering that fructose is detected in human breast milk and significantly increases after drinking a high-fructose corn syrup-sweetened beverage^18, 19, 58^. Nevertheless, we show for the first time that a common constituent of Western diet, high fructose, directly impacts microglia phagocytosis and neurodevelopment. How this factors into human adolescent development itself, given the recent rise in mood and anxiety disorder development, especially post-COVID19 pandemic, remains an important public health question.

## Materials and Methods

### Contact for Reagent and Resource Sharing

For further information and requests for resources and reagents should be directed to the Lead Contact, Justin S. A. Perry (perryj@mskcc.org).

### Mice

Wild-type C57BL/6J mice (Stock No. 000664) were purchased from The Jackson Laboratory. *Slc2a5*^–/–^ (KO) mice were originally generated by Wu et al.^28^ and provided by M. Zeng. For experiments using standard diet (SD), dams were provided PicoLab Rodent Diet 5053 (LabDiet 5053, PMI). All mice were housed at Memorial Sloan Kettering Cancer Center (MSKCC) or Weill Cornell Medicine under specific pathogen-free (SPF) conditions with 12-hour light/dark cycles and were cared for by Research Animal Resource Center (RARC). Mice were housed under ambient conditions and provided *ad libitum* access to water and food. All studies conducted were approved by the Sloan Kettering Institute (SKI) and Weill Cornell Medicine Institutional Animal Care and Use Committee (IACUC).

To generate conditional knockout mice (cKO), cryopreserved sperm for the C57BL/6N-*Slc2a5*^tm1a(EUCOMM)Wtsi/Ieg^ (ID: 07602) strain were obtained from the European Mutant Mouse Archive (EMMA). Sperm carrying the tm1a KO first/pre-conditional allele and oocytes from WT donor females were used to perform IVF at the MSKCC Mouse Genetics Core Facility. Heterozygous mice were then crossed to the FLPo deleter strain B6.129S4-Gt(ROSA)26Sor^tm2(FLP*)Sor/J^ (JAX stock number 012930) to eliminate the lacZ reporter and neo cassette and generate a clean conditional floxed allele. Homozygous *Slc2a5*^fl/fl^ mice with loxP sites flanking exon 6 of the gene were crossed to FVB-Tg(Csf1r-icre)1Jwp/J (JAX 021024) to ultimate generate *Slc2a5*^fl/fl^ cre negative or *Slc2a5*^fl/fl^ CSF1r-cre positive mice (always heterozygous for CSF1r-cre) for experiments. For each dietary condition, *Slc2a5*^fl/fl^ cre negative females were paired with *Slc2a5*^fl/fl^ Csf1r-cre positive males to generate litters containing cKO mice and WT littermate controls.

### Early life high fructose diet and delivery

Gamma irradiated isocaloric control, high fructose, and high glucose diets are modified forms of the AIN-93G Formula generated by Research Diets, Inc. Control diet (CD, D19060506) contains 0 kcal% sucrose, high fructose diet (HF, D19060507) contains 15 kcal% fructose, and high glucose diet (HG, D19082305i) contains 15 kcal% glucose. Dams of each cohort (CD and HF) were placed on respective diets at least one week before mating and diets were maintained throughout gestation and lactation. For intragastric delivery of fructose, mice received 50 μL of sterile water with or without fructose at 45 mg/mouse. Solutions were delivered daily via injections into the visible milk spot of wildtype (WT) or *Slc2a5*^–/–^ neonates from perinatal day (P)1 to P7. The selected fructose dosage is 45% of the typical 100 mg/mouse dose delivered daily by oral gavage to adult mice which was determined to be equivalent to 3% of total daily caloric intake and analogous to human consumption of less than 12 oz of sugar sweetened beverage^59^. Timed matings were used for cross-fostering experiments, in which litters born to dams on high fructose diet were switched with litters born to dams on control diet on P0. These litters were either exposed to high fructose during gestation but control diet during lactation or were exposed to control diet during gestation but high fructose diet during lactation. Adult mice for weight experiments were weighed daily for 3 weeks while on either control, high fructose, or high glucose diet.

### Immunofluorescence and confocal microscopy

Neonatal mice were anesthetized with CO_2_, perfused with 10 mL cold PBS (Corning, 21-040-CM), and harvested brains were submerged in 4% PFA (StatLab, 28530-1) for 24-48 hours at 4°C. Tissues were then washed 3x in PBS, embedded in 2% SeaPlaque Agarose (Lonza, 50101), and sectioned by vibratome (Leica, VT1000S) into 50 μm coronal sections. Sections were placed in 12 or 24 well plates for permeabilization using 1% Triton X-100 (Sigma-Aldrich, T9284) and 5% BSA (Sigma-Aldrich, A7888) in PBS overnight at 4°C with rotation. After 3x PBS washes, free floating sections were blocked with 5% normal goat serum (Jackson ImmunoResearch, 005-000-121) and 0.1% Triton X-100 in PBS (blocking buffer) for 1 hour at room temperature. Sections were incubated for 48-72 hours with the following primary antibodies: rabbit anti-Iba1 (1:400; Wako, 019-19741), rat anti-CD68 (Bio-Rad MCA1957), and mouse anti-PSD-95 (Sigma-Aldrich, MAB1598). Sections were then washed 3x with PBS containing 0.05% Tween 20 and incubated with Alexa Fluorophore conjugated goat anti-rabbit IgG (H+L) secondary antibody Alexa Fluor 488 (Thermo Fisher, A-11034) and goat anti-mouse Alexa Fluor Plus 647 (Thermo Fisher, A32728) at 1:400 in blocking buffer covered at room temperature for 2 hours with rotation. Sections were washed again 3x with PBS containing 0.05% Tween 20 (Sigma-Aldrich, P1379) but with the addition of 1:2000 Hoechst 33342 (Thermo Fisher, H3570) during the second wash before mounting with ProLong Gold antifade reagent (Thermo Fisher, P36934). For TUNEL staining, manufacturer’s instructions for the In Situ Cell Death Detection Kit, TMR red or Fluorescein (Roche, 12156792910 or 11684795910), were followed before mounting. For TUNEL and density, images of neonatal brain sections were taken as 20 μm Z-stacks on a Zeiss LSM 980 (20x objective, 20 μm thick image stacks, 2 μm step-size). For each mouse, a minimum of 4 fields of view (FOV) were obtained from the prefrontal cortex before maximum intensity z projections were created in Fiji (ImageJ2) for quantification and sem-automated morphological analysis. For imaging of the hippocampus, a 10x objective was used to acquire images encompassing the CA1, CA2, CA3, and DG regions. Max intensity z projections were compiled from 20X images to best capture larger population level effects.

For 3D reconstruction, confocal microscopy was performed using a Zeiss LSM 980 (63x oil immersion objective, 20 μm thick image stacks, 0.3 μm step-size). Immunofluorescence staining was performed as described above with an additional primary antibody, guinea-pig anti-VGLUT1 (Millipore Sigma AB5905). Imaris software (v10.2; Bitplane) and its Surface segmentation module was used to quantify Iba1, CD68, PSD95, and VGLUT1 volumes by applying 3D surface rendering of z-stacks in individual channels and establishing identical intensity and voxel thresholds. To quantify engulfment of PSD95 and VGLUT1, only puncta contained within CD68+ volumes inside Iba1+ cells were assessed using the mask function. Consistent with established methods, we normalized to Iba1+ cell volume^40, 60, 61^.

Imaging of synaptophysin and PSD95 in the prefrontal cortex of adolescent WT and cKO mice after the conclusion of behavioral experiments was performed as described in Chu et al^17^. Immunofluorescence staining was performed as described above with an additional primary antibody, mouse anti-synaptophysin (Millipore Sigma S5768). Confocal microscopy was performed using a Zeiss LSM 980 (63x oil immersion objective, 5 um thick image stacks, 0.5 um step-size) and 5 FOVs were acquired for each mouse. Signal area was analyzed using Fiji.

### Analysis of microglia morphology

Iba1-labeled microglia in images obtained by confocal microscopy (20x objective, 20 μm thick, 2 μm step-size) were converted to gray scale using the MorphoLibJ plugin in Fiji (opening filter, area minimum: 25 pixels, connectivity: 8) and aligned to an octagon morphological filter element (radius pixels: 1) as previously described^62^. Threshold detection levels were adjusted to align automated microglia counts with previously obtained cell counts in Fiji to optimize detection of complete cell somas. Microglia were analyzed using the Analyze Particles function with a size limit of 10 pixels to exclude background noise. Major axis length was derived from the longest axis of an ellipse fit to each microglia soma. A combination of major axis length and soma area were used to compute a cell roundness score per cell (Roundness $=\frac{4A}{\pi M^{2}},\text{A}=$ soma area, M = major axis length)^62^, and then cell roundness values were averaged per field of view (4 FOVs per animal). For analysis of ramification and branching, identical images were imported into Fiji and filtered by the Unsharp Mask function (radius: 3 pixels). A new threshold was obtained to capture branching and junctions. The Close binary function was applied and the binary skeletonized as previously described^63^. Once skeletonized, despeckling and outlier removal were applied as needed and branching metrics exported for analysis.

### Mixed glial cultures

Meninges were removed from cortices harvested from WT P2-P4 neonates in cold HBSS (Thermo Fisher, 14025092). The dissected cortices were then manually dissociated in HBSS and spun at 350 G for 5 min. The homogenate was resuspended in 10 mL warm DMEM (Corning, 10-017-CV) supplemented with 10% (vol/vol) heat-inactivated FBS (Sigma, 12306C), 100 U/mL of penicillin and 100 μg/mL streptomycin (Gibco, 15140-122), and 2 mM L-glutamine. Cortices from two neonates were cultured in each Poly-D-Lysine (Thermo Fisher A3890401) coated T25 flask with 10 mL DMEM with or without 5 mM fructose, with osmolarity controlled by mannitol (Sigma-Aldrich, M4125). Mixed glial cultures were grown in a standard incubator in humidified 5% CO_2_ at 37°C. Media was changed 2 days later and then every 3-4 days. After 18 days, microglia were shaken off at 200 rpm at 37 °C for 1 hour and quantified per flask using the Countess II (Thermo Fisher) to obtain mixed glial culture yield for both control and fructose treated flasks.

### Primary microglia isolation and culture

Neonatal brains were harvested from P2-P4 WT and *Slc2a5* deficient mice (KO or cKO) and dissociated using the Miltenyi gentleMACS Octo Dissociator with Heaters, brain dissociation kits (Miltenyi Biotec 130-096-427, 130-107-677, 130-092-628), and gentleMACS C Tubes (Miltenyi Biotech 130-093-237), following manufacturer’s instructions. Microglia were purified using CD11b MicroBeads (Miltenyi Biotec 130-097-142), LS Columns (Miltenyi Biotech 130-042-401) and the QuadroMACS separator (Miltenyi Biotech 130-091-051), following manufacturer’s instructions. Up to 5 brains per pooled in each C Tube, and cKO mice were genotyped immediately prior to isolation to enable pooling. Isolated primary microglia were then cultured in previously established serum-free TIC media^30^ containing key factors including TGF-β2, IL34, and cholesterol, but modified to use DMEM without glucose (Gibco, A14430-01) to create specific conditions with various glucose (Sigma-Aldrich, 68270) and fructose (Sigma-Aldrich, F0127) concentrations along with the nonmetabolizable sugar mannitol to control for osmolarity. For *in vitro* conditioning, microglia were grown in standard tissue culture incubator conditions, except with 3.5% O_2_ in the BioSpherix X3 Xvivo System.

### Microglia phagocytosis assays

For all experiments, microglia from P2-P4 WT and *Slc2a5*^–/–^ (KO or cKO) mice were isolated and incubated as described above with serum free media conditions containing indicated hexose combinations. For synaptosome phagocytosis assays, synaptosomes were first purified from WT adult mice using the Syn-PER Synaptic Protein Extraction Reagent according to manufacturer’s instructions (Thermo Scientific, 87793). Synaptosomes were then stained with CypHer5E (GE Life Sciences, PA15401) via agitation, washing, and sonication. A BCA assay was used to determine the staining CypHer5E concentration (10 mM CypHer5E/50 uL). CypHer5E+ synaptosomes were added to each chambered well (Nunc, 155409) conditioned at 3.5% O_2_ with hexose concentrations corresponding to the growth condition for each well (0 mM, 1 mM, or 5 mM fructose with constant 5 mM glucose, and 20 mM, 19 mM, or 15 mM mannitol, respectively, to control for osmolarity). Phagocytosis was assessed using time-lapse confocal microscopy on a Zeiss Axio Observer Z1-7 inverted fluorescence microscope fully encased in a black-out environmental chamber, equipped with a Z PIEZO stage encapsulated with a heating/gas-controlled insert, a Zeiss 20x PlanApo (0.8 NA) objective, a 6-channel, 7-laser LSM 980 (405nm, 445nm, 488nm, 514nm, 561nm, 594nm, 639nm), and a Airyscan 2 multiplex detector. Data was acquired using Zen Blue software (Zeiss). Chambered glass slides containing cells were removed from 3.5% O_2_ at appropriate time intervals so that images for each condition could be immediately taken at the same time point. For each condition, 4-6 regions containing similar cell numbers were selected and imaged. After using identical microscope settings for each condition, analysis was performed after applying fixed thresholds in Fiji for CypHer signal intensity across all images within each experiment.

For apoptotic neuron efferocytosis assays, apoptosis was induced in the Neuro-2a (N2A) neuroblastoma cell line (ATCC, CCL-131) using 0.25 μM staurosporine (Cayman Chemical, 81590) for 12-14 hours. Primary microglia were isolated, spot plated (50,000 cells in 50 μL) in 24 well plates and conditioned for one week as described above. Microglia were incubated with CypHer stained apoptotic N2As (1uM for 45min) at a 1:1 phagocyte:target ratio for 30 min at 3.5% O_2_, scraped off wells, and CypHer5E+ microglia were analyzed using an Attune NxT flow cytometer (software v. 3.2.1; Thermo Fisher). Samples were analyzed with FlowJo v10.9 (BD). When primary microglia are used as phagocytes, there is an inherent difference in the absolute percentage uptake of corpses between experiments performed on different days. Therefore, phagocytic index was used to compile data from multiple flow cytometry experiments. Phagocytic index = percent engulfment (experimental/control).

For HK2 inhibition experiments, BV2 microglia and N2A cells were maintained in DMEM (Corning, 10-017-CV) supplemented with 10% (vol/vol) heat-inactivated fetal bovine serum (Sigma, 12306C), 100 U/mL of penicillin and 100ug/mL streptomycin (Gibco, 15140-122), and 2 mM L-glutamine, at 37°C and 5% CO2 in humidified incubators. BV2 microglia were conditioned as described above for 72 hours, with the HK2 inhibitor 3-BP (30 μM) added 48 hours prior to engulfment. Apoptotic N2A cells were fed at a 0.5:1 target-to-phagocyte-ratio. CypHer5E+ microglia were analyzed by flow cytometry.

### Human pluripotent stem cell-derived microglia generation

Human pluripotent stem cells (PSCs) were generated from anonymized human PBMCs based on published protocols^64^ using Sendai viral vectors (Thermo Fisher, A16517) driving high expression of pluripotency markers NANOG and OCT4, which were confirmed by flow cytometry. PSCs were maintained on CF1 mouse embryonic fibroblasts (Thermo Fisher, A34181) in Embryonic Stem Cell (ESC) medium (Thermo Fisher, KO-DMEM 10829-018; 20% KO serum replacement 10828-028; 2 mM L-glutamine 25030-024; 1% nonessential amino acids 11140-035; 1% penicillin/streptomycin 15140163; 0.2% β-mercaptoethanol 31350-010) supplemented with 10 ng/mL basic fibroblast growth factor bFGF (PeproTech, 100-18B). The strategy for PSC hematopoietic differentiation was adapted from a previously published protocol^65^ in which embryoid bodies (EBs) were formed by dissociating and maintaining PSCs in ESC media supplemented with 10 μM Rho-associated protein kinase (ROCK) inhibitor (Sigma, Y0503) while being kept on an orbital shaker at 100 rpm for 6 days to allow for spontaneous formation of EBs with hematopoietic potential. At day 6 of differentiation, 200-500 μm cystic EBs were selected under a dissecting microscope and transferred onto adherent tissue culture plates (~2.5 EBs/cm^2^) for cultivation in Hematopoietic Differentiation (HD) medium (APEL 2, Stem Cell Tech, 05270; Protein Free Hybridoma, Thermo Fisher, 12040077; penicillin and streptomycin, Thermo Fisher, 15140163) from day 6 up to day 18 from the start of differentiation, supplemented with 25 ng/mL human IL-3 (Peprotech, 200-03) and 50 ng/mL human M-CSF (Peprotech, 300-25). At day 18 of differentiation, macrophages produced by EBs were collected from suspension and cultivated on tissue culture plates at a density of ~15,000 cells/cm^2^ in RPMI (Thermo Fisher, 61870036) supplemented with 10% FBS and human M-CSF (100 ng/mL) for 6 days before use for downstream experiments. Engulfment assays using PSC derived cells were performed as described above for primary microglia, except cells were plated at 15,000/cm^2^ in 24 well plates with constant 10 mM glucose plus 0 mM fructose and 15 mM mannitol (to control for osmolarity), 5 mM fructose and 10 mM mannitol, or 15 mM fructose and 0 mM mannitol.

### [2-^13^C]-fructose NMR

WT and *Slc2a5*^–/–^ P7 neonatal mice born to dams on HF or CD were injected intraperitoneally via the quadriceps with 4 g/kg [2-^13^C]-fructose (Cambridge Isotope Laboratories, CLM-1527) in PBS (20 mg in 200 μL PBS). Brains were harvested and flash frozen in liquid nitrogen. Sample extraction was performed using methanol (Sigma-Aldrich, 34860), chloroform (Sigma-Aldrich, 366927), beads (Fisherbrand 15-340-151), and a Bead Mill (Fisherbrand 15-340-163). Brain extracts were spun in a centrifugal evaporator (GeneVac) for 5 hours, and pellets were dissolved in 600 μL of 1 mM TSP, 10 mM imidazole, and 0.2 wt% sodium azide in deuterium oxide. After sonication for 30 minutes, the solutions were vortexed and added to NMR tubes. Carbon and proton NMR was performed using 1 mM TSP as a chemical shift and concentration internal standard.

### ManCou14 flow cytometry

ManCou14 was synthesized as previously described^36^, and 1mM Mancou14 was obtained by diluting a 10 mM stock in DMSO with PBS. 200 μL was injected into WT and KO mice between 8-10 weeks of age via tail vein. After 30 min, animals were euthanized with CO2 and perfused with cold PBS and 5 mM EDTA (Invitrogen, 15575-038). Brains were minced and incubated at 37°C in PBS containing collagenase D (Millipore Sigma, 11088866001), 5% heat-inactivated FBS (Sigma, 12306C), and 10 mM HEPES (Gibco, 15630-080). Enzymatic activity was halted by incubating with 10 mM EDTA for 5 min. Tissues were then dissociated using 70 μm cell strainers and resuspension. After centrifugation at 1500 rpm for 5 min at 4°C, cell pellets were resuspended in 38% isotonic Percoll (10% 10X PBS and 90% Percoll, Cytiva, 17-0891-01) and centrifuged at 2000 rpm for 30 min at RT with no brake. After washing and red blood cell lysis (Millipore Sigma R7757), samples were incubated with anti-mouse CD16/32 antibody (1:50, Bio X Cell, BE0307) for 10 min on ice. Samples were then further incubated on ice with the following fluorochrome conjugated antibodies: anti-mouse CD11b FITC (11-0112-82 eBioscience), anti-mouse CD45 APC (BioLegend 103112), and anti-mouse CX3CR1 BV785 (BioLegend 149029). After 15 min, samples were washed and then analyzed on a Cytek Aurora, given Mancou14’s excitation/emission (λmax) of 366nm/452nm. Flow cytometry data were analyzed using FlowJo v10.10 (BD Biosciences), and microglia were gated on CD45intCX3CR1+CD11b+ cells (Extended Data Fig. 8b).

### [U-^13^C]-fructose tracing metabolomics

Primary microglia were isolated as described above using CD11b microbeads. One million cells were pooled from 4-5 P2-P4 neonates for each of the three biological replicates per condition. Cells were cultured for 1 week at 3.5% O_2_ in 1 mM or 5 mM fructose plus constant 5 mM glucose in TIC media, with a media change after 4 days. On the final day all media conditions were replaced with 5 mM [U-^13^C]-fructose (Cambridge Isotope Laboratories, CLM-1553). Metabolites were extracted using 80% methanol 24 hours later, on dry ice. The supernatants containing polar metabolites were dried down and dissolved in water. Targeted LC-MS analyses were performed on a Q Exactive Orbitrap mass spectrometer (Thermo Fisher) in polarity-switching mode, coupled to a Vanquish UPLC system (Thermo Fisher). A Sequant ZIC-HILIC column (2.1 mm i.d. × 150 mm, Merck) was used for separation of metabolites. Flow rate was set to 150 μL/min. Buffers consisted of 100% acetonitrile for mobile B, and 0.1% NH_4_OH/20 mM CH_3_COONH_4_ in water for mobile A. Gradient was from 85% to 30% B in 20 min followed by a wash with 30% B and re-equilibration at 85% B. Data analysis was performed using El-MAVEN (v0.12.0). Metabolites and their ^13^C isotopologues were identified based on exact mass within 5 ppm and standard retention times. Relative metabolite quantitation was performed based on peak area for each metabolite.

### HK2 mitochondrial colocalization

WT and cKO microglia were isolated, plated, and conditioned at 3.5% O2 as described above. MitoTracker Red CMXRos (Thermo Fisher Scientific M7512) was added 15 min before CypHer labeled apoptotic N2A cells were added for 30 min of engulfment. Cells in chamber slides were then immediately fixed in 4% PFA upon exiting 3.5% O2 for 10 min at RT. Samples were washed 3x with PBS, permeabilized with 0.1% Triton-X100 for 10 min, and washed 3x again with PBS. Samples were then blocked with 4% donkey serum (Jackson ImmunoResearch 017-000-121) and 1% BSA in PBS (blocking buffer) at RT for 1 hour, and incubated with goat anti-Iba1 (1:200, Wako 011-27991) and rabbit anti-hexokinase 2 antibody (1:200, Abcam ab227198) in blocking buffer overnight at 4°C. After 3x washes with PBS, samples were incubated with donkey anti-rabbit IgG (H+L) Alexa Fluor Plus 405 (1:400, Invitrogen A48258) and donkey anti-goat IgG (H+L) Alexa Fluor Plus 488 (1:400, Invitrogen A32814) in blocking buffer covered at RT for 2 hours. After 3x washes with PBS, coverslips were added to chamber slides with Prolong gold. Z-stack images (7 μm, 0.5 μm step-size) were obtained using a 63x objective for each condition, and max intensity Z-projections were generated in Fiji. ROIs were generated for individual cells containing apoptotic corpses, and colocalization analysis was performed using the BIOP JACoP^66^ plugin in Fiji to obtain a Pearson’s coefficient for each cell.

### Novel object recognition

The novel object recognition (NOR) task was conducted as previously described^67, 68^. Briefly, all tests were conducted in a plastic box measuring 29 cm × 47 cm × 30 cm, with stimuli consisting of similarly sized plastic objects that varied in color and shape. A camera mounted directly above the box recorded all testing sessions for video analysis. One day prior to testing, mice were acclimated to the room and chamber, with 30 min in the testing room and 5 min to explore the empty arena. Mice were placed in the same arena 24 hours later in the presence of two identical objects and were allowed to explore for 5 min. After an intersession interval of 1 hour, mice were placed in the same box, with one of the two objects replaced by a novel object, and were allowed to explore for 5 min. Exploratory behavior was assessed manually, and exploration of an object was defined as a mouse sniffing the object or touching the object while looking at it. Placing the forepaws on the objects was considered as exploratory behavior, but climbing on the objects was not. Minimal exploration time (10 sec) for both objects during the test phase was used, and no significant difference in total exploration time was detected. The amount of time taken to explore the novel object was expressed as a percentage of the total exploration time and used to provide an index of recognition memory. Any-maze v7.1 (Stoelting) was used for acquisition and analysis of data.

### Barnes Maze

The Barnes maze test was performed as previously described^67, 68^. Briefly, the maze consists of a circular open surface (90 cm in diameter) elevated to 90 cm and 20 circular holes (5 cm in diameter) equally spaced around the perimeter that were positioned 2.5 cm from the edge. A wooden escape box (11 × 6 × 5 cm) was positioned beneath one of the holes, and aversive stimuli included neon lamps and a buzzer. Extra-maze visual cues consisted of objects within the room including a table, computer, sink, door, plus the experimenter. No wall or intra-maze visual cues were placed around the edge of the maze. The ANY-maze tracking system (Stoelting) was used to record the movement of mice on the maze. Between trials, mice were placed into cages in a dark room adjacent to the test room for the intertrial interval (20-30 min). The acquisition phase consisted of three consecutive training days (days 1-3), with three trials per day during which the escape hole was located at the same location. For each trial, a mouse was placed into a start tube located in the center of the maze, the start tube was raised, and the buzzer was turned on until the mouse entered the escape hole. Between trials, the maze and escape box were cleaned with 10% ethanol in water to minimize olfactory cues. Mice were given 3 min to locate the escape hole for each trial and were guided to the escape box if they failed to enter the escape hole. Parameters of learning performance that were recorded included: latency to locate the escape hole (primary latency), latency to enter the escape hole (total latency), the number of errors made before locating the escape hole (primary errors), and the number of errors made before entering the escape hole (total errors). A mouse dipping its head into any hole not containing the escape box was considered an error. On days 4 and 5, the location of the escape hole was moved 180° from its previous location and two trials per day were performed.

### Fear conditioning and extinction

Fear conditioning and extinction assays were performed as previously described^17^. Briefly, mice were placed in shock chambers (Coulbourn Instruments) and were fear conditioned after 2 min of habituation with 3 tone-shock pairings consisting of a 30 sec (5 kHz, 70 dB) tone (conditioned stimulus, CS) that co-terminated with a 1 sec (0.7 mA) foot shock (unconditioned stimulus, US). The intertrial intervals (ITIs) between each tone-shock pairing were 30 sec. Mice remained in the conditioning chambers after the final tone-shock pairing for 1 min. For classical fear extinction with one session per day for 3 days, 24 hours after fear conditioning, mice were placed in extinction chambers, habituated for 2 min, and then exposed to 5 presentations of the tone (CS) in the absence of the shock (US), with each tone lasting for 30 sec with an ITI of 30 sec (5 trials on days 1 and 2, and 20 trials on day 3 to confirm maximal extinction). Mice remained in the extinction chambers for 1 min after the final tone presentation. Experiments were performed using Graphic State software (Coulbourn instruments) and all mice were video recorded for analysis. Freezing behavior was scored using previously validated MATLAB code and percent time spent freezing (freezing %) was calculated by dividing the amount of time spent freezing during the 30 sec tone presentations by tone duration.

### Reverse transcription quantitative PCR

Total RNA was extracted from microglia after CD11b microbead isolation from P2-P4 and P7 WT or *Slc2a5* deficient (KO or cKO) neonates born to and nursed by dams on standard diet, control diet, or high fructose diet using the NucleoSpin RNA kit (Macherey-Nagel, 740955.250). The QuantiTect Reverse Transcription Kit (Qiagen, 205314) was used to synthesize cDNA according to the manufacturer’s instruction. Quantitative gene expression for mouse and human genes was performed using TaqMan probes and TaqMan Fast Advanced Master Mix (Applied Biosystems/Thermo Fisher, 4444554) with a QuantStudio 6 Pro (Applied Biosystems). For murine *Slc2a5*, 10 commercially available probes collectively spanning all 14 exons of the gene were tested and quantified relative to 18S. Details for TaqMan probes are listed in Extended Data Fig. 6.

### Western blot

For SLC2A5 western blotting, primary microglia were isolated as described above, and cell pellets containing 1 million cells each were lysed in RIPA buffer (Thermo Fisher, 89900) with protease and phosphatase inhibitors (Thermo Fisher, 78440). Lysates were passed through a syringe with an 18G needle, spun at max speed for 20 min, and quantified using the BCA protein assay (Thermo Fisher, 23227) according to manufacturer’s instructions. Lysates were not boiled prior to loading, and 40 μg of each protein lysate were resolved on 4-12% Bis-Tris gels (Thermo Fisher, NP0322BOX) under denaturing conditions. Proteins were blotted onto a nitrocellulose membrane (Thermo Fisher, LC2009) and probed with anti-SLC2A5 (Santa Cruz, sc-271055) and anti-GAPDH (Cell Signaling Technology, 2118) antibodies. Membranes were visualized using an anti-mouse HRP-conjugated secondary antibody (Cytiva, NA931), ECL reagent (RPN2209) and film (Fisher Scientific, NC9556985).

For HK2 western blotting, 1 million BV2 cells for each media condition were conditioned for 72 hours at 3.5% O2, washed with PBS, and scraped from 6 well plates. Samples were lysed on ice for 10 min in RIPA Buffer (Thermo Scientific 89901) supplemented with Halt Protease and Phosphatase Inhibitor Cocktails (Thermo Scientific 78443), and were then centrifuged at 14,000 rpm for 10 min at 4°C. Supernatant was combined with 4x Laemmli Sample Buffer (Bio-Rad 1610747) containing beta-mercaptoethanol (Thermo Fisher Scientific 31350010) and treated at 95°C for 5 min. Samples were then run in pre-cast NuPAGE Bis-Tris Mini Protein Gels 4-12% (Thermo Fisher Scientific NP0335BOX) and NuPAGE MOPS SDS Running Buffer (Thermo Fisher Scientific NP0001), and were transferred onto PVDF using a semi-dry transfer protocol (Bio-Rad 1704272). Membranes were incubated in blocking buffer, comprised of TBST containing 5% non-fat dry milk (Fisher Scientific NC0115668), for 1 hour and then incubated overnight at 4°C in blocking buffer containing rabbit anti-hexokinase 2 (1:200, Cell Signaling Technology 2867S) and mouse anti-beta actin (1:10,000, Thermo Fisher Scientific MA5-15739) primary antibodies. Membranes were then washed with TBST 4x for 15 min at RT and incubated with blocking buffer containing anti-rabbit (1:2,000, Cytiva NA934V) and anti-mouse (1:2,000, Cytiva NA931V) HRP conjugated secondary antibodies for 2 hours at RT. After 4x washes with TBST, membranes were incubated with SuperSignal West Pico PLUS Chemiluminescent Substrate (Thermo Fisher Scientific 34578) and imaged on an iBright 1500 (Thermo Fisher Scientific).

### Statistics and reproducibility

Data were analyzed using GraphPad Prism 10.4.1. Significance was determined using one of the following: two-tailed unpaired t-test, Welch’s t-test, one-way ANOVA, or two-way ANOVA. Microglia from 4-5 P2-P4 neonates of the same genotype were pooled in independent experiments in order to generate sufficient material for U-^13^C fructose tracing and phagocytosis assays. A minimum of 4 fields of view (FOVs) per animal or condition were analyzed by confocal microscopy. Statistics were performed based on the mean for each group of animals for each condition. Where representative data is shown, observations were confirmed across all replicates.

## Extended Data

## Supplementary Material

Extended data file

Supplementary Information

## Data Availability

All data supporting the findings of this study are available within this manuscript, in the Source Data file, and in Supplementary Information. Extended Data Fig. 5 consists of secondary analysis of publicly available datasets^69–76^.
