# Supplementary material for: Early life high fructose exposure disrupts microglia phagocytosis and impedes neurodevelopment
Source: Nature. Author manuscript; Available in PMC 2025 Aug 21. (PMC7617807; doi:10.1038/s41586-025-09098-5)

Extended Data Fig. 1

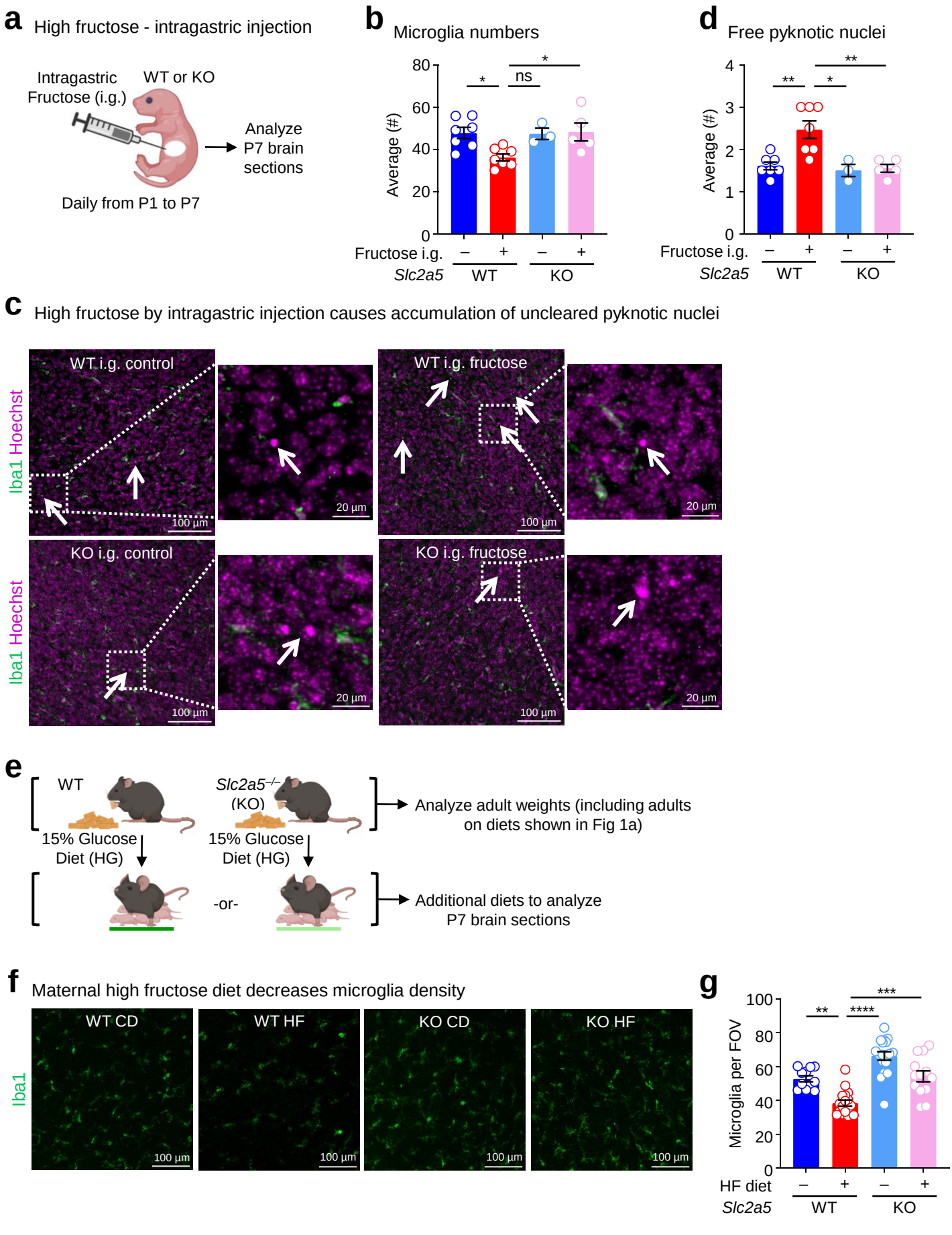

Extended Data Fig. 2

a High fructose diet does not affect branch number/length

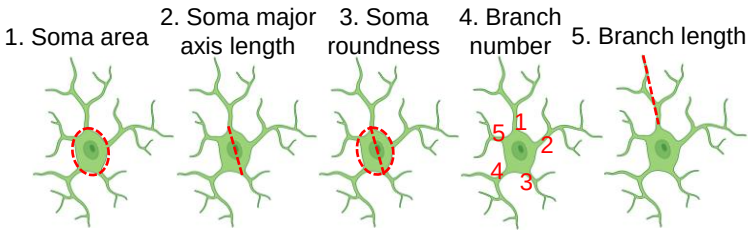

b

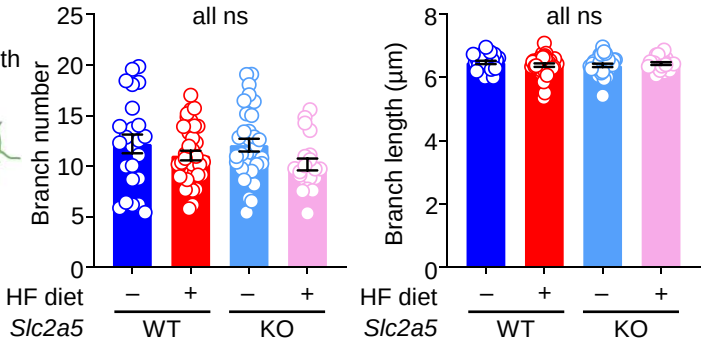

c High fructose diet induces quiescent microglia morphology

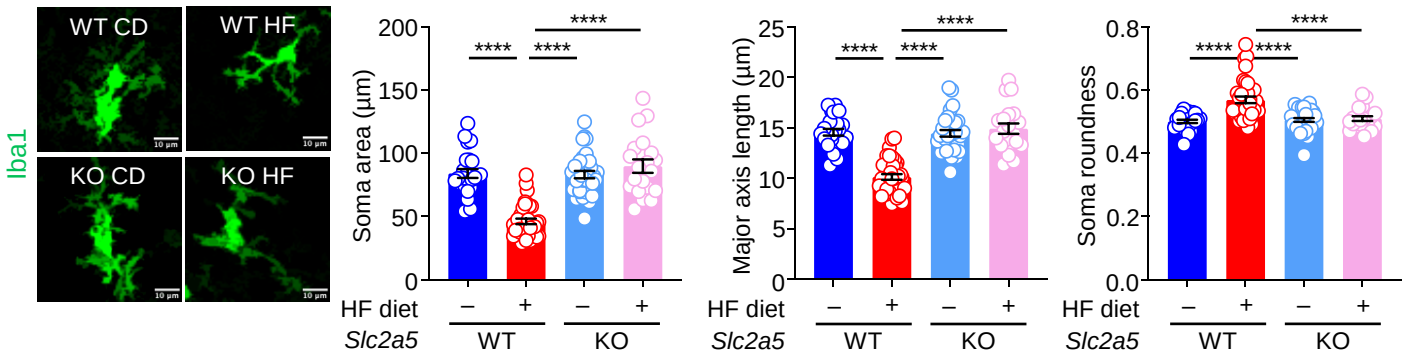

Extended Data Fig. 3

a High fructose diet causes accumulation of uncleared pyknotic nuclei

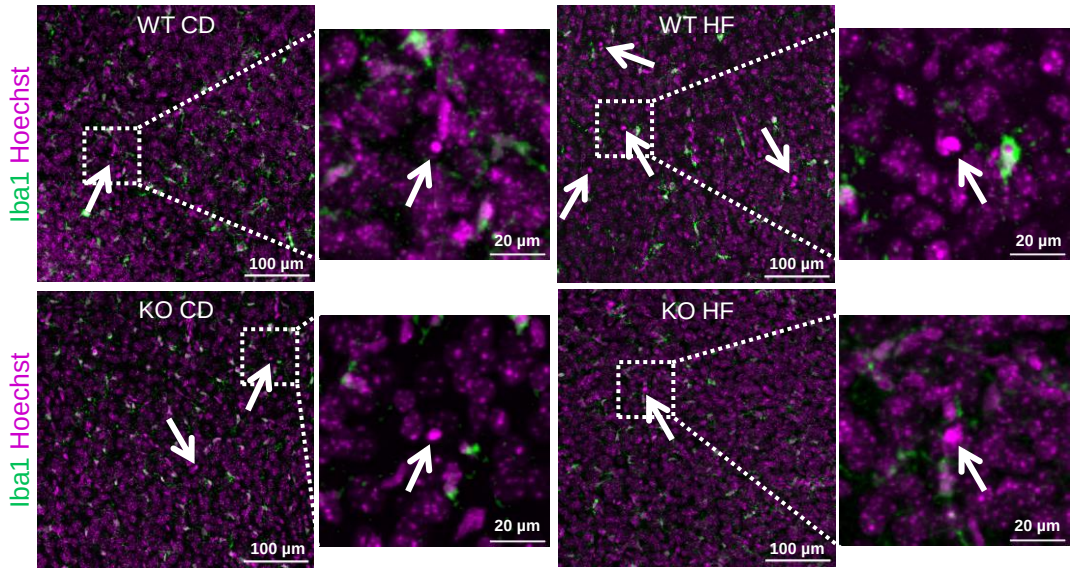

b Free pyknotic nuclei

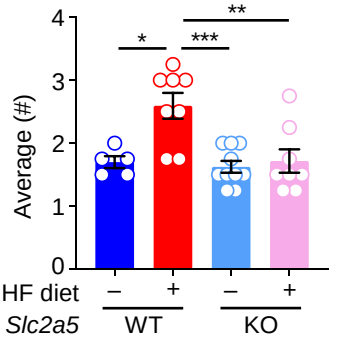

c TUNEL bound/internalized vs free examples

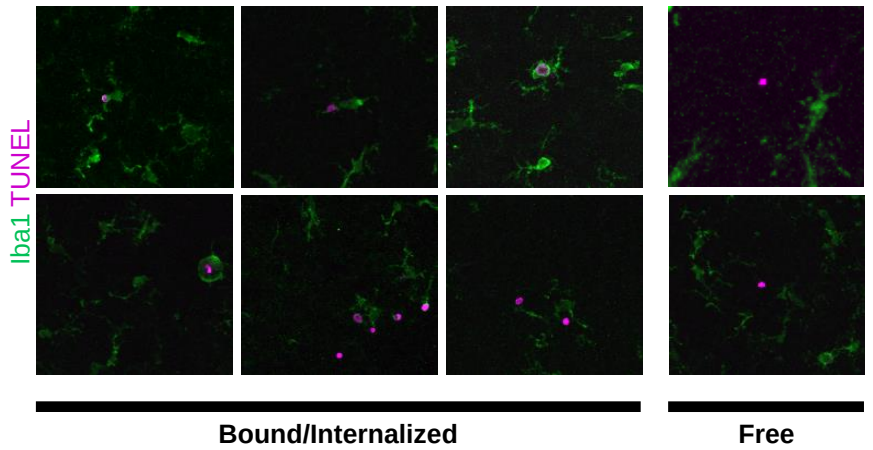

d High fructose diet impairs engulfment of PSD95+ material

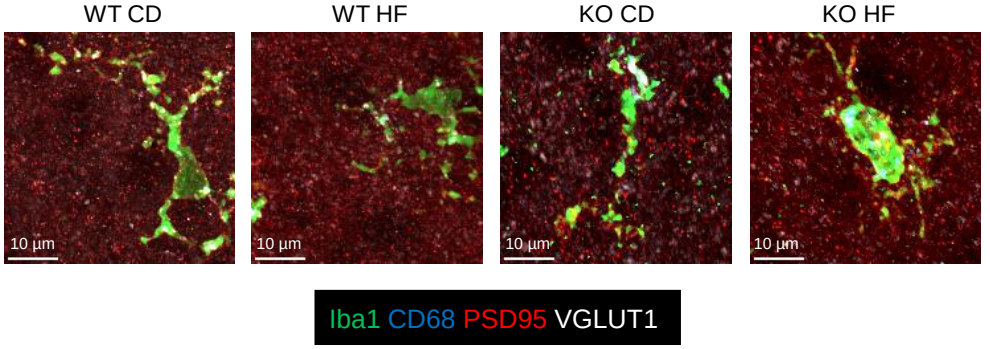

e VGLUT1 engulfment

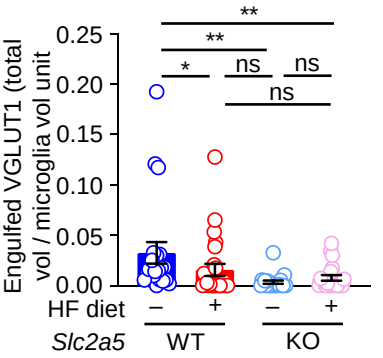

Extended Data Fig. 4

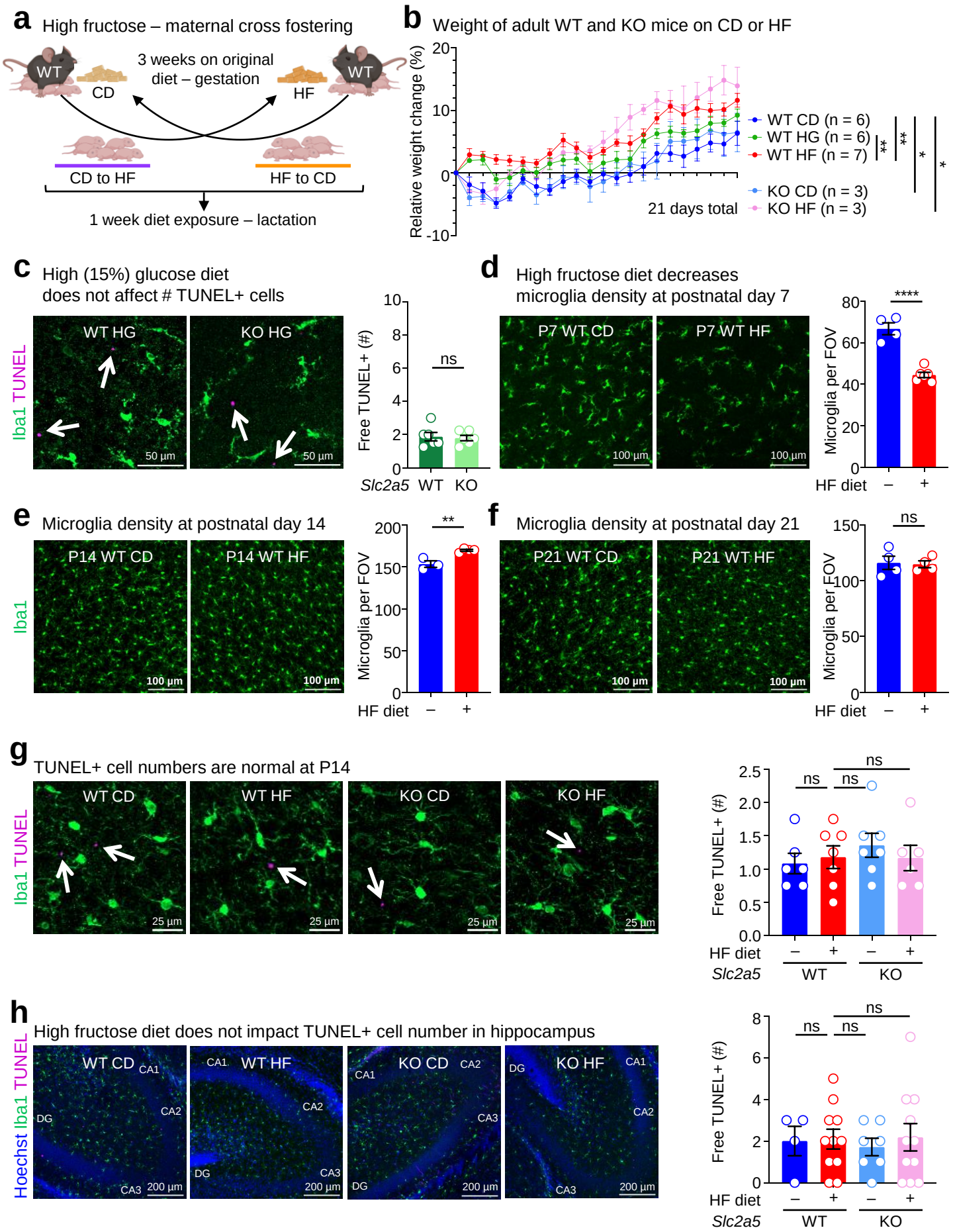

Extended Data Fig. 5

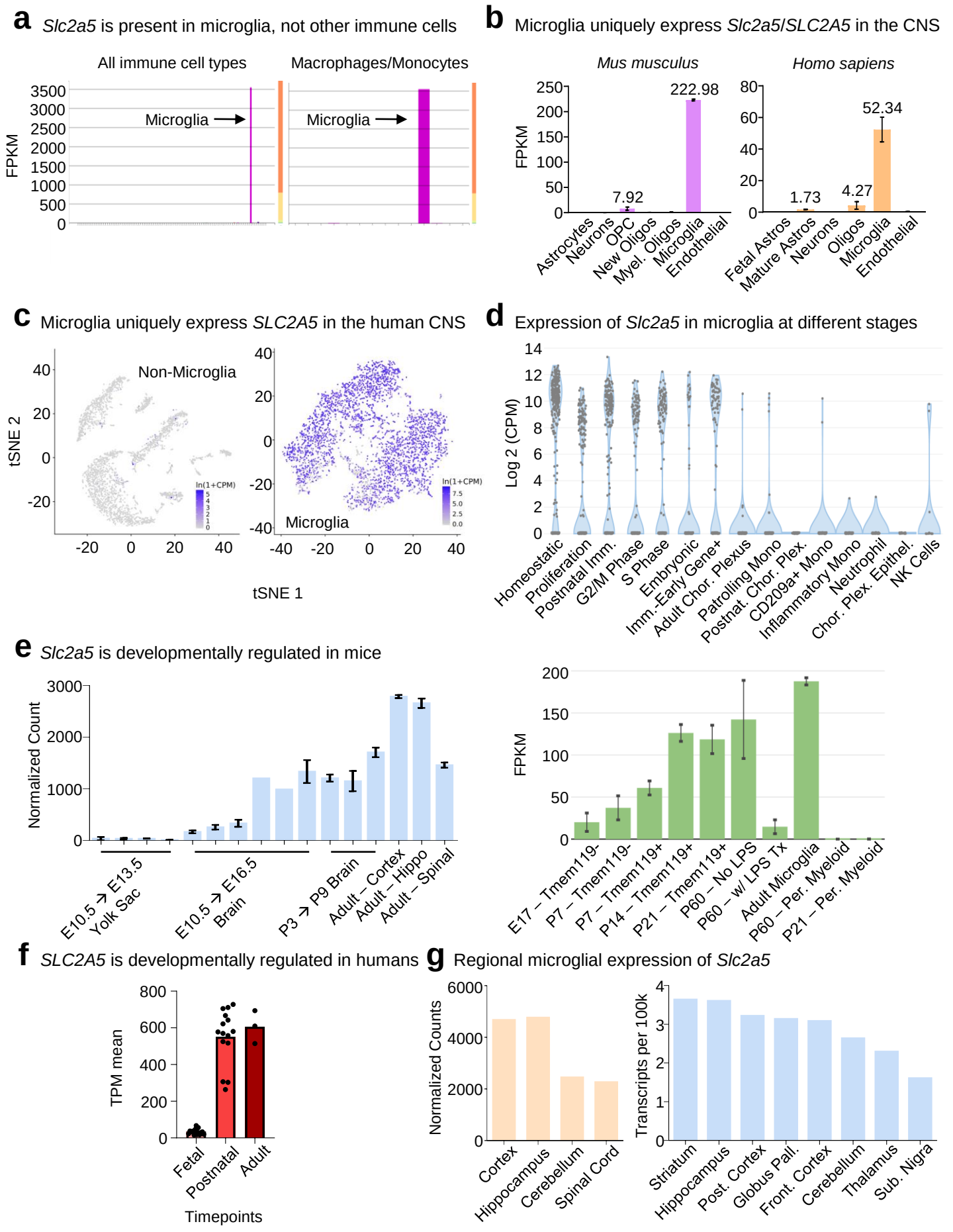

Extended Data Fig. 6

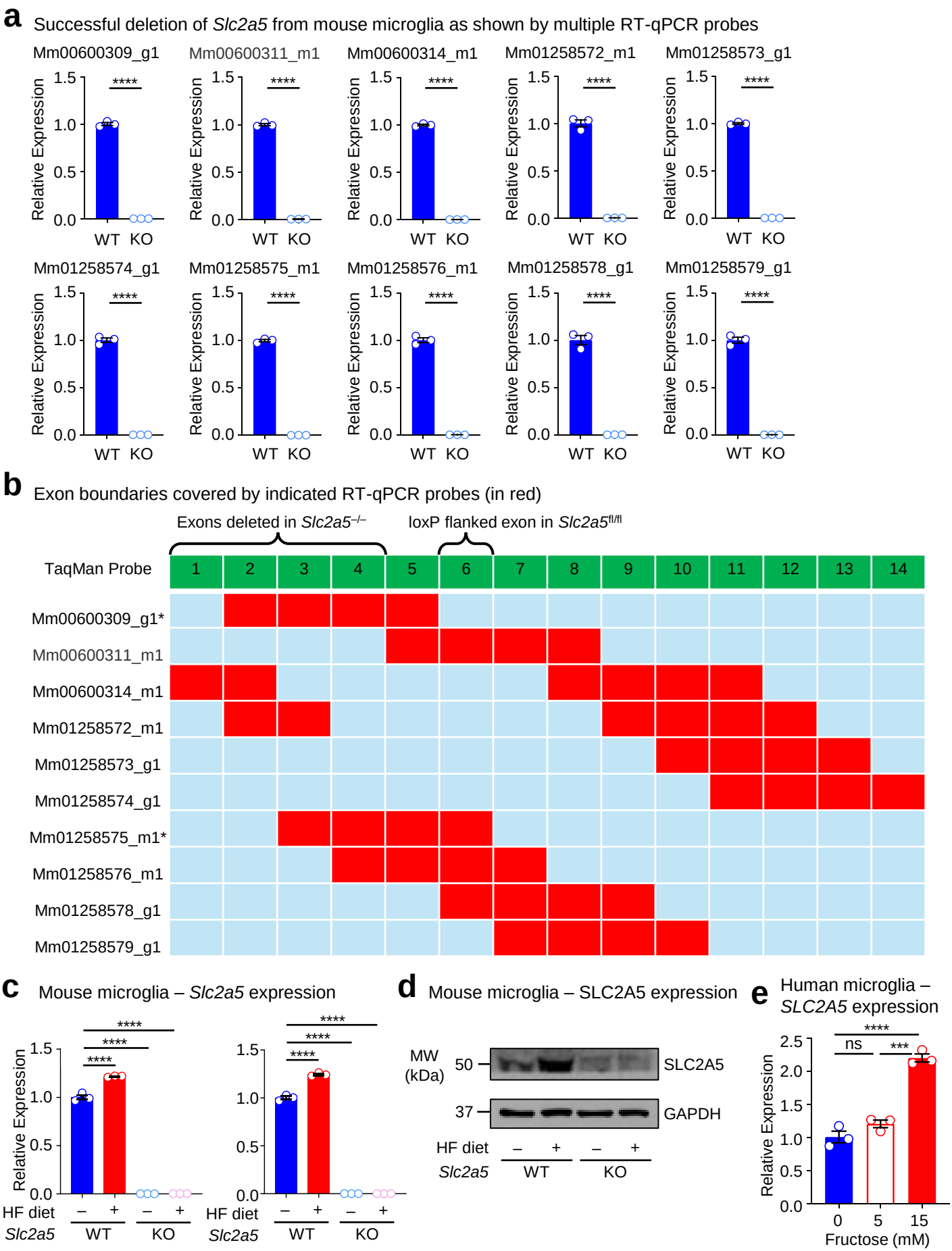

Extended Data Fig. 7

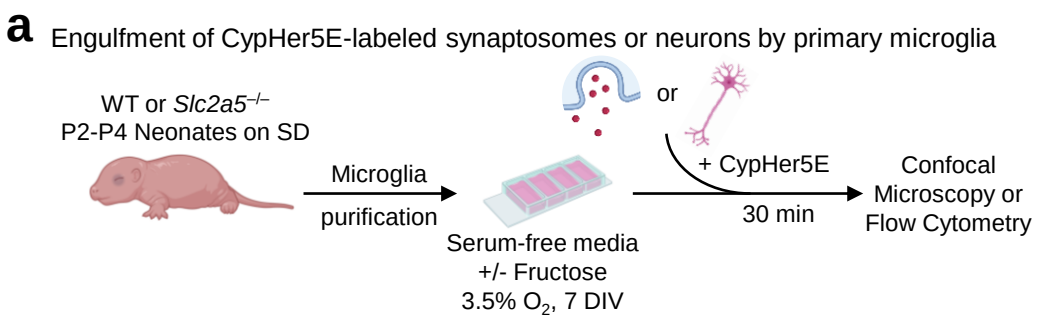

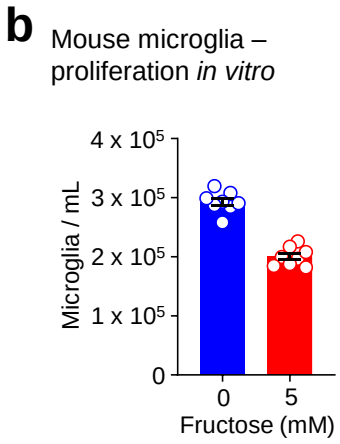

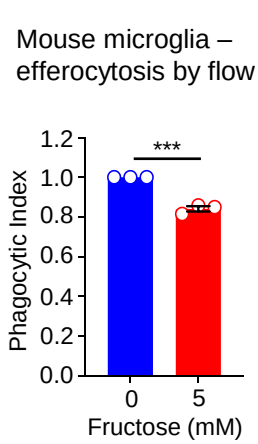

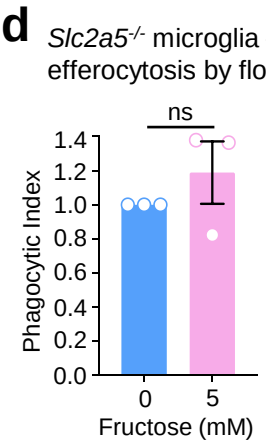

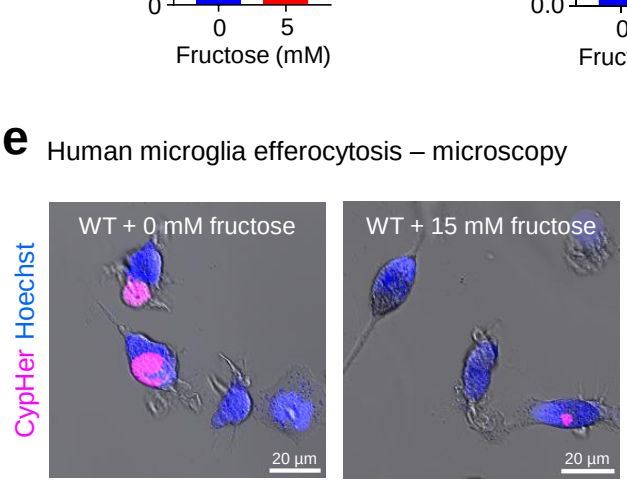

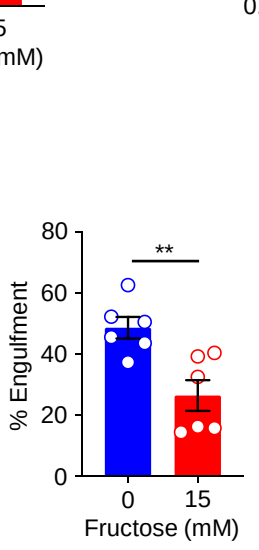

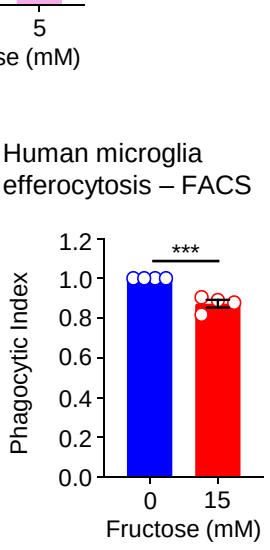

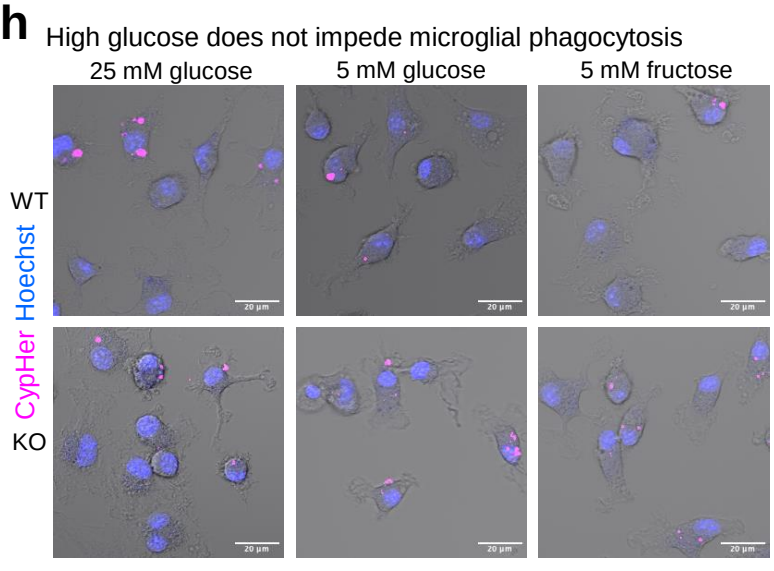

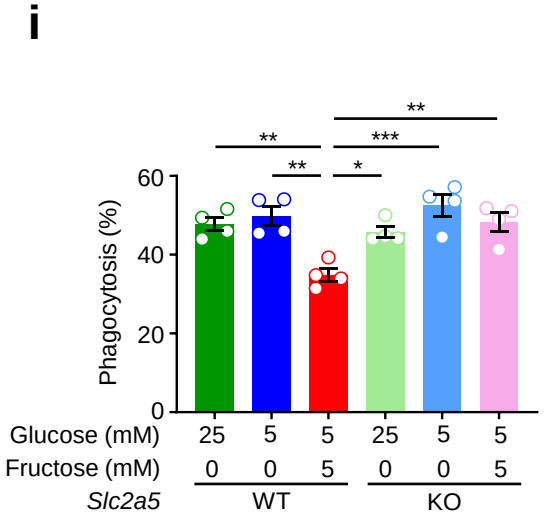

Extended Data Fig. 8

a Uptake of SLC2A5 specific probe ManCou14 by primary microglia

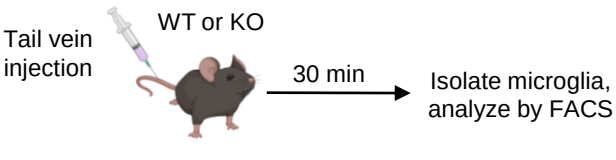

Excitation/Emission (λ_{max}): 366nm/452nm

b ManCou14 – gating strategy

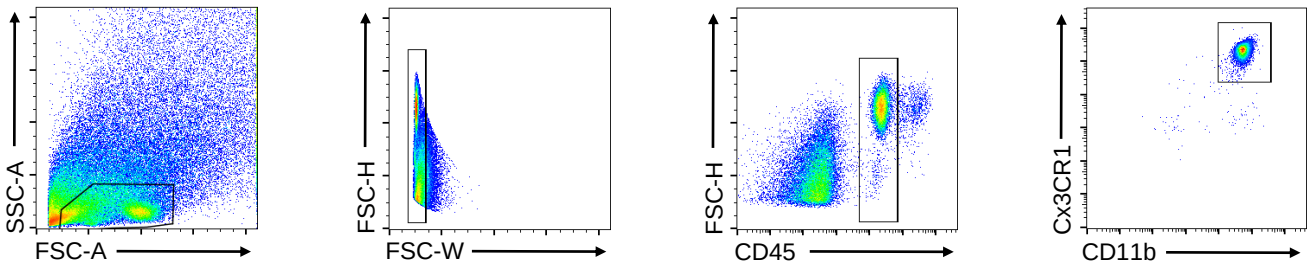

c Expression of CD11b and CX3CR1

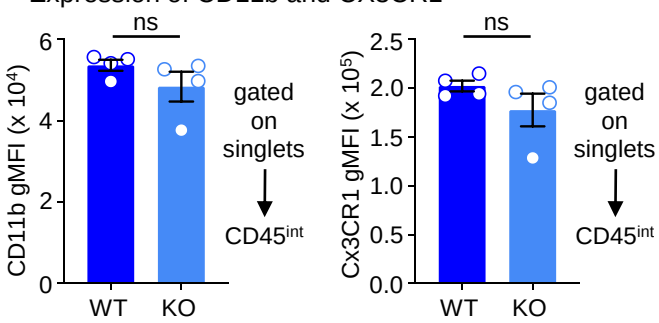

d Fructofuranose interactions with Glut5$_{out}$

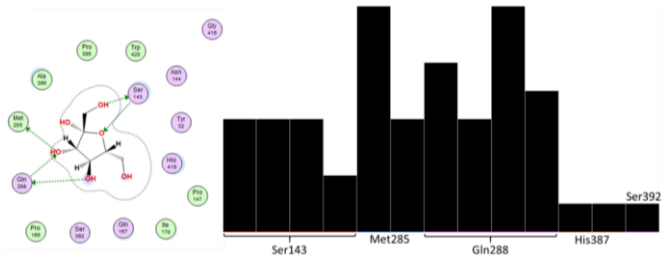

e ManCou14 interactions with Glut5$_{out}$

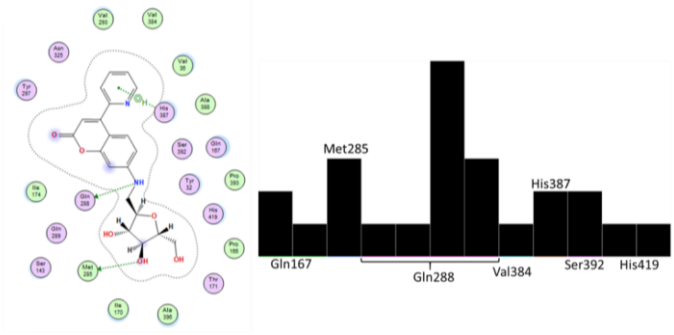

f Docking of fructofuranose (yellow) and ManCou14 (gray) in the binding site of GLUT5

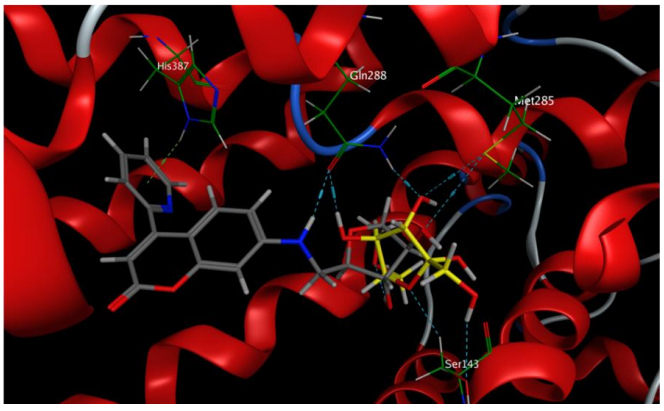

Extended Data Fig. 9

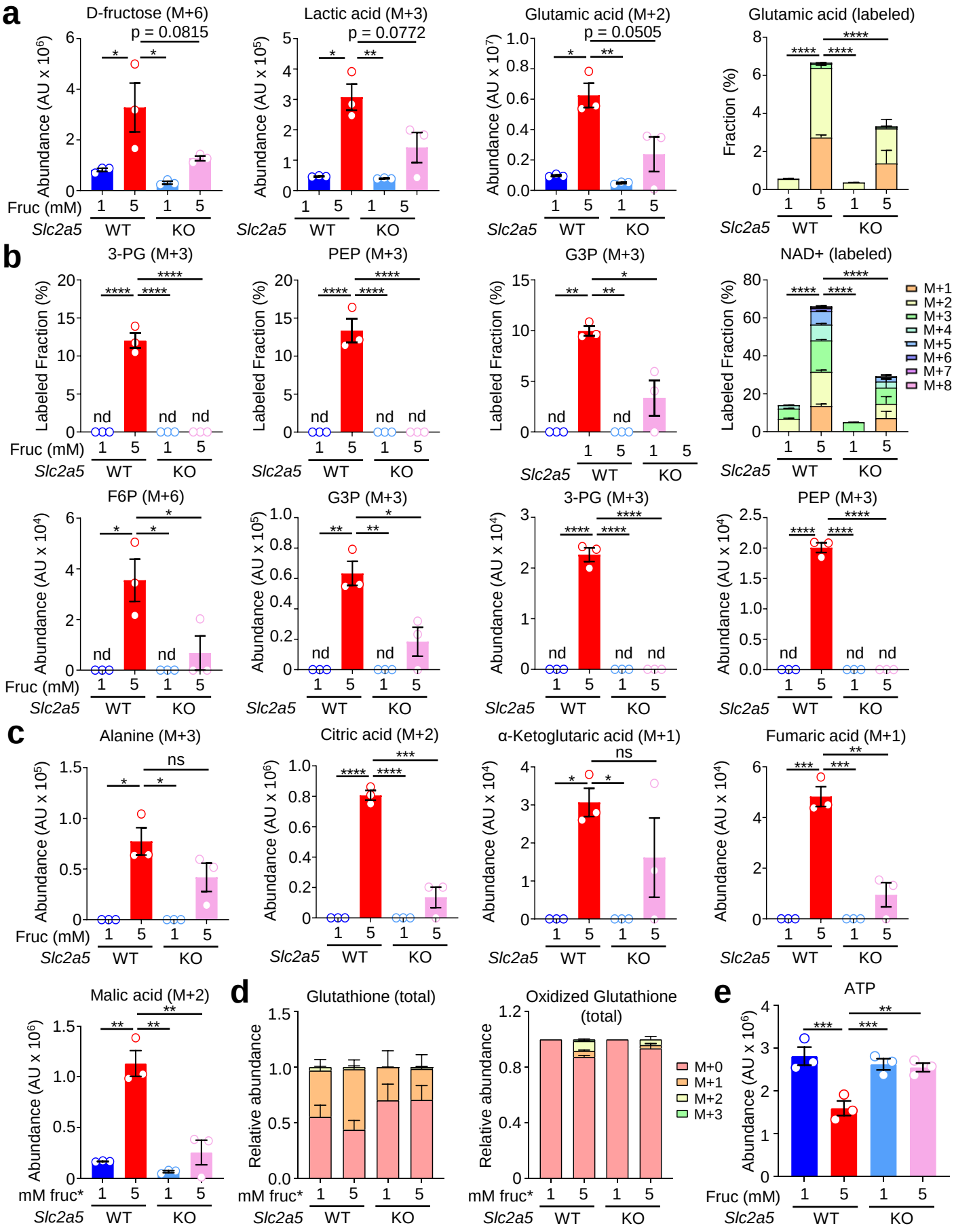

Extended Data Fig. 10

a HK2 inhibition rescues phagocytosis

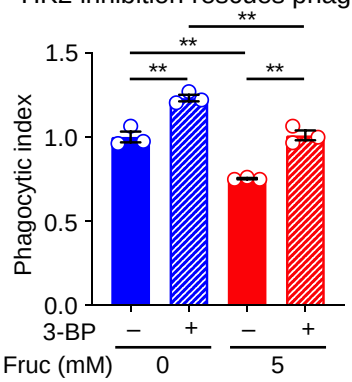

b HK2 protein expression

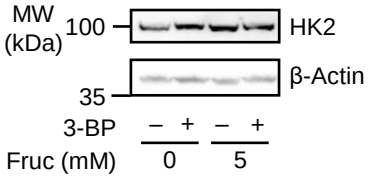

d HK2 mitochondrial colocalization

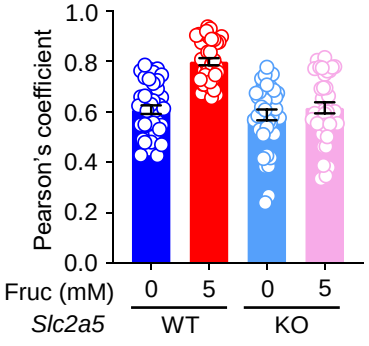

c Analysis of HK2 localization in glucose or high fructose treated microglia

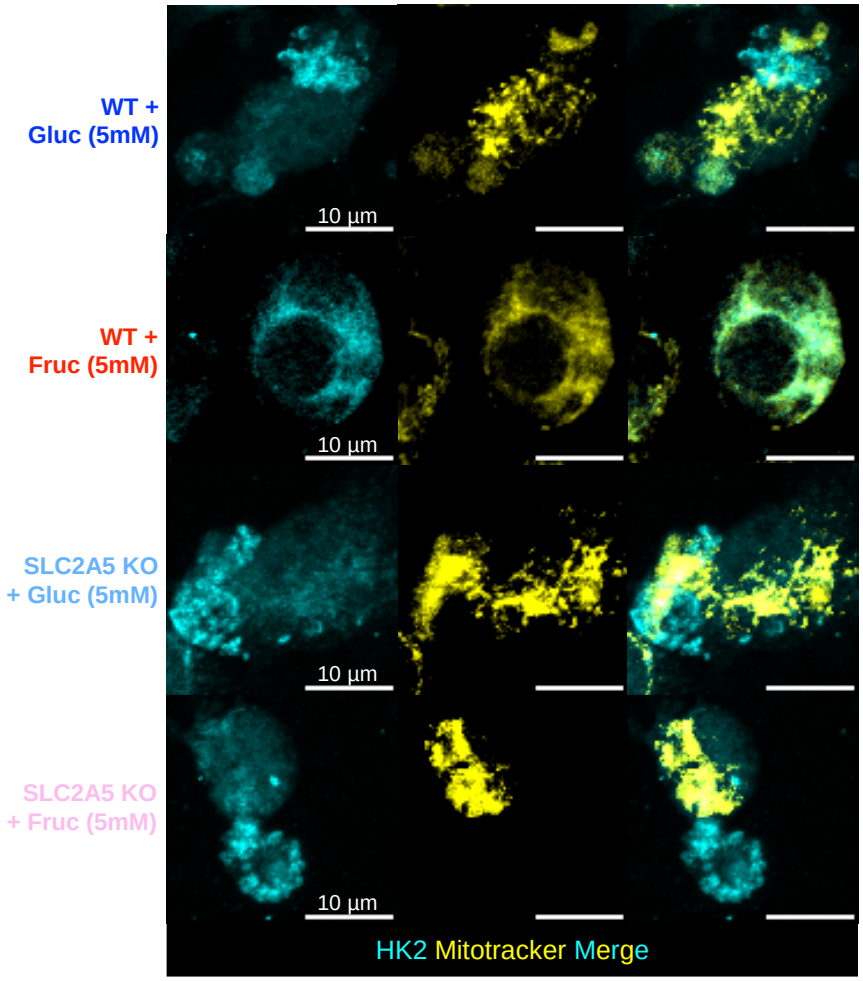

Extended Data Fig. 11

a High fructose exposure does not impair spatial or working memory

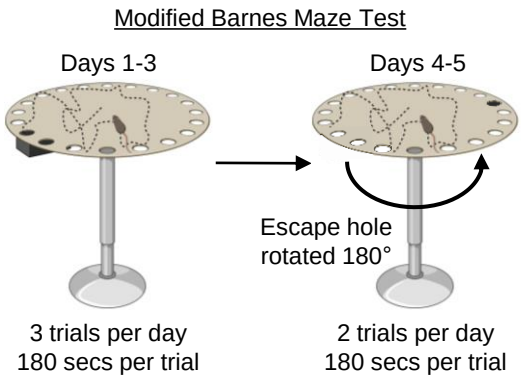

b

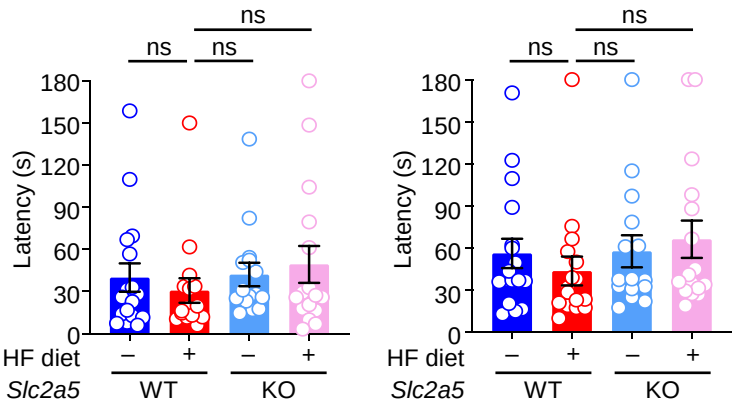

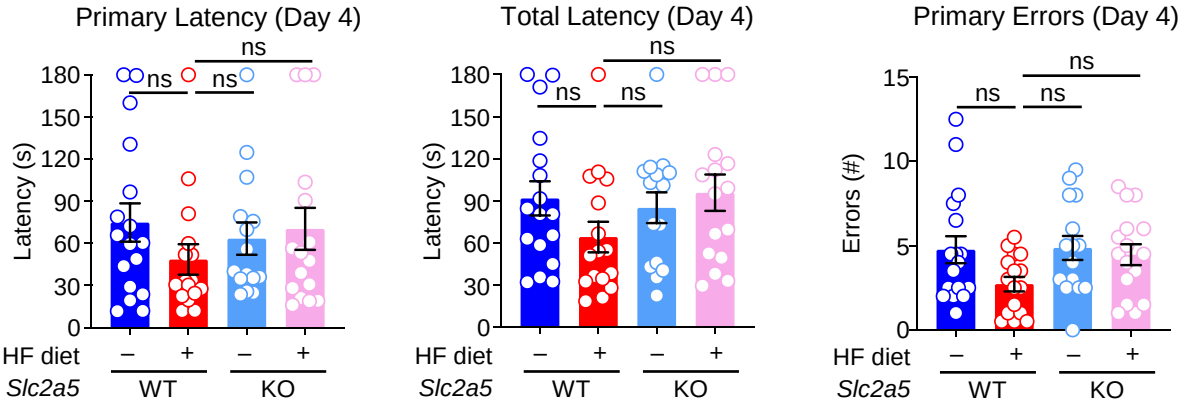

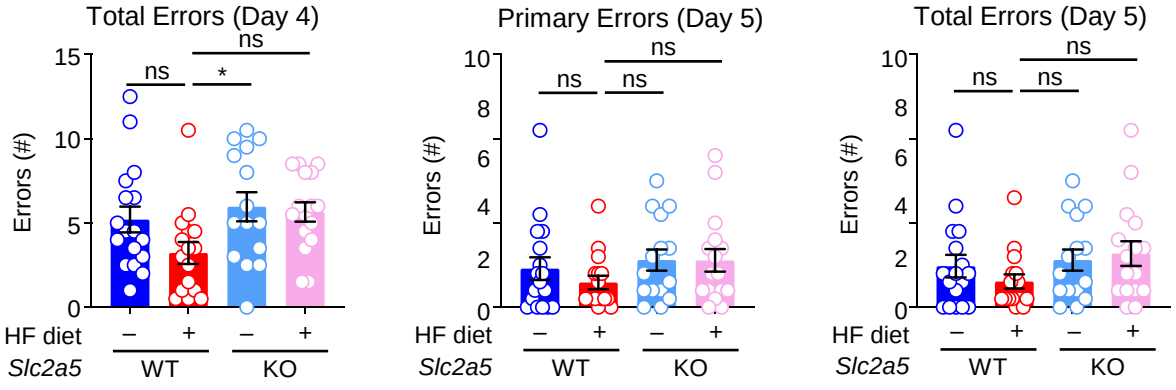

Extended Data Fig. 12

a Deletion of *Slc2a5* in cKO shown by RT-qPCR

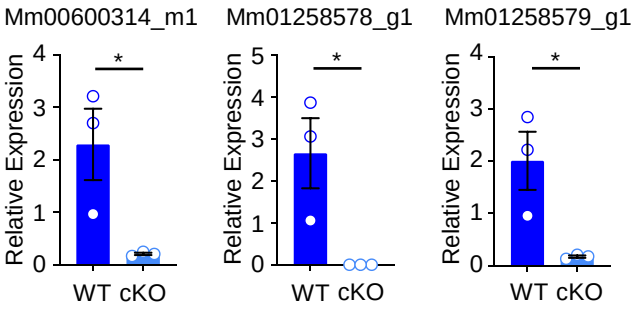

b Schematic of WT and cKO mice on CD or HF diet

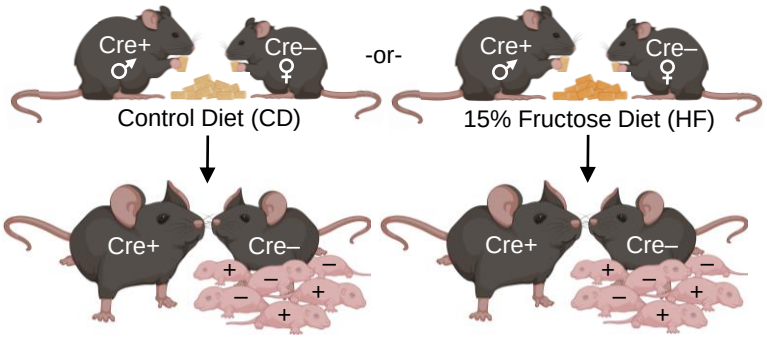

c Microglia density in WT and cKO mice

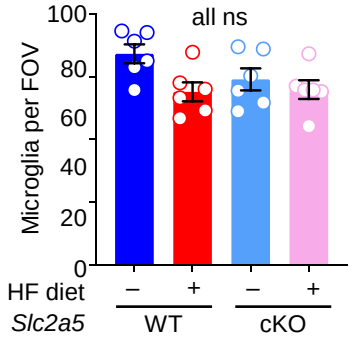

d Conditional deletion of *Slc2a5* rescues the increase in TUNEL+ cells

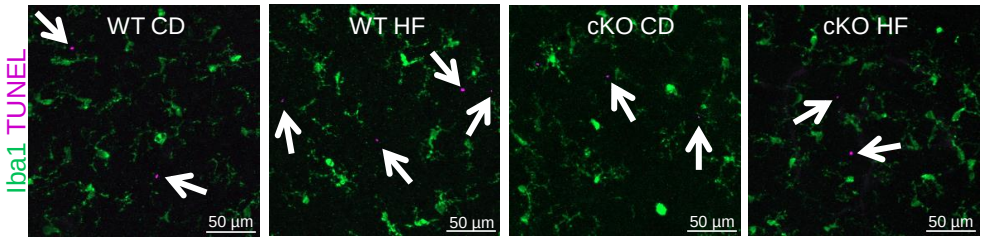

e Max intensity z projections corresponding to Imaris images from WT and cKO mice

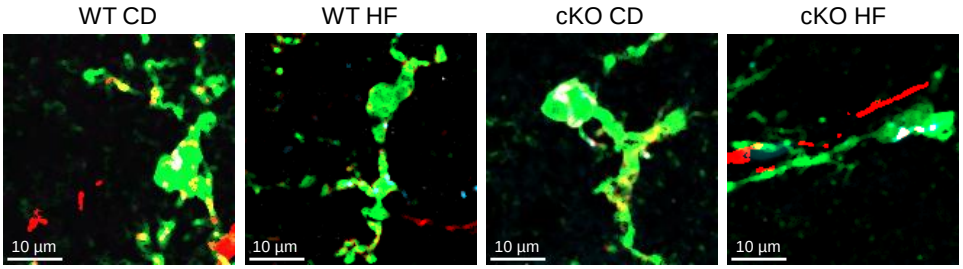

f VGLUT1 engulfment

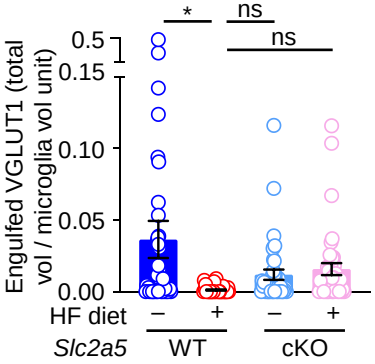

g Synaptosome engulfment in WT or cKO cells

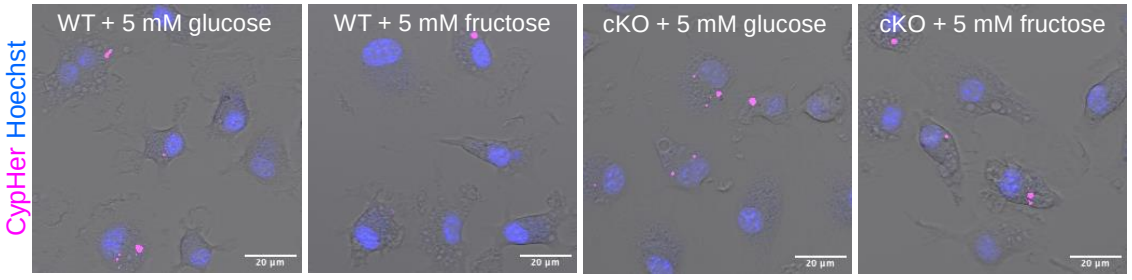

Extended Data Fig. 13

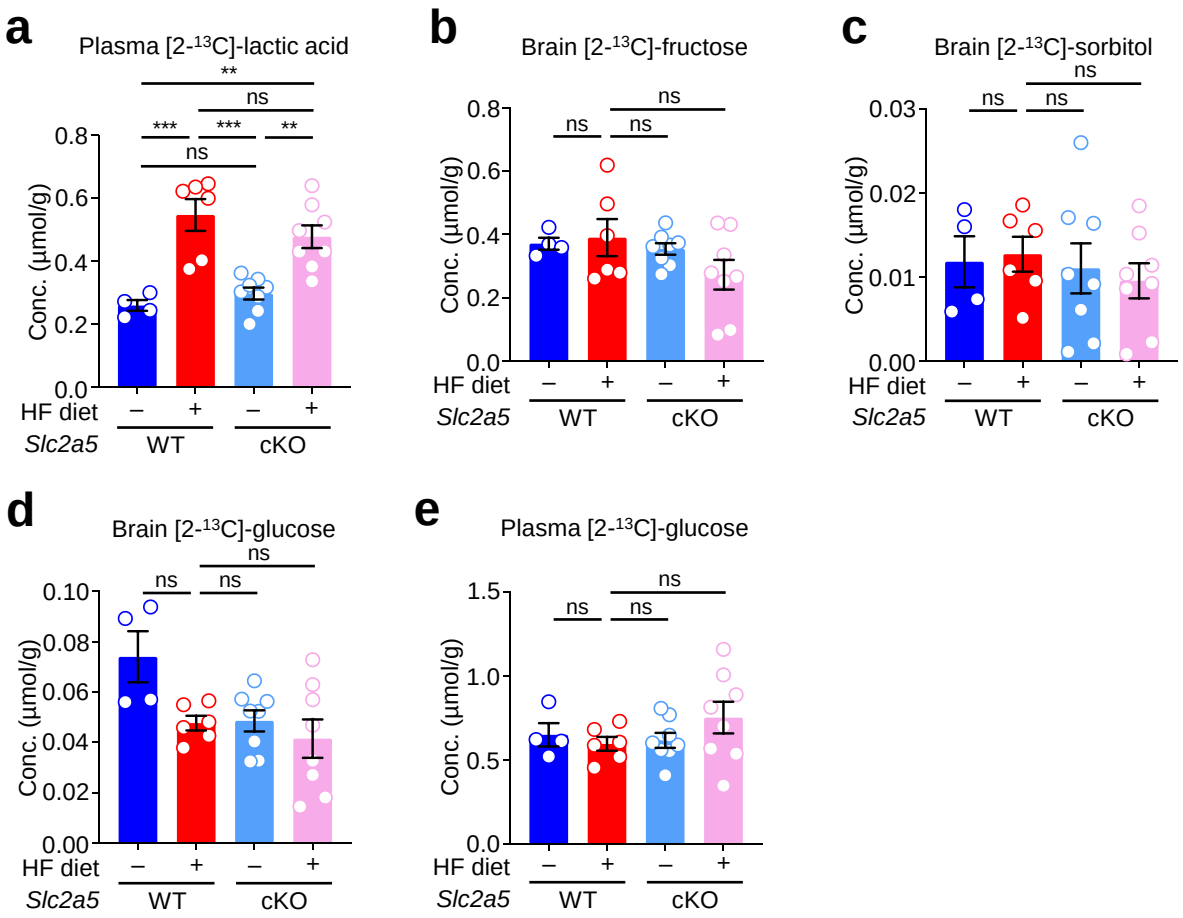

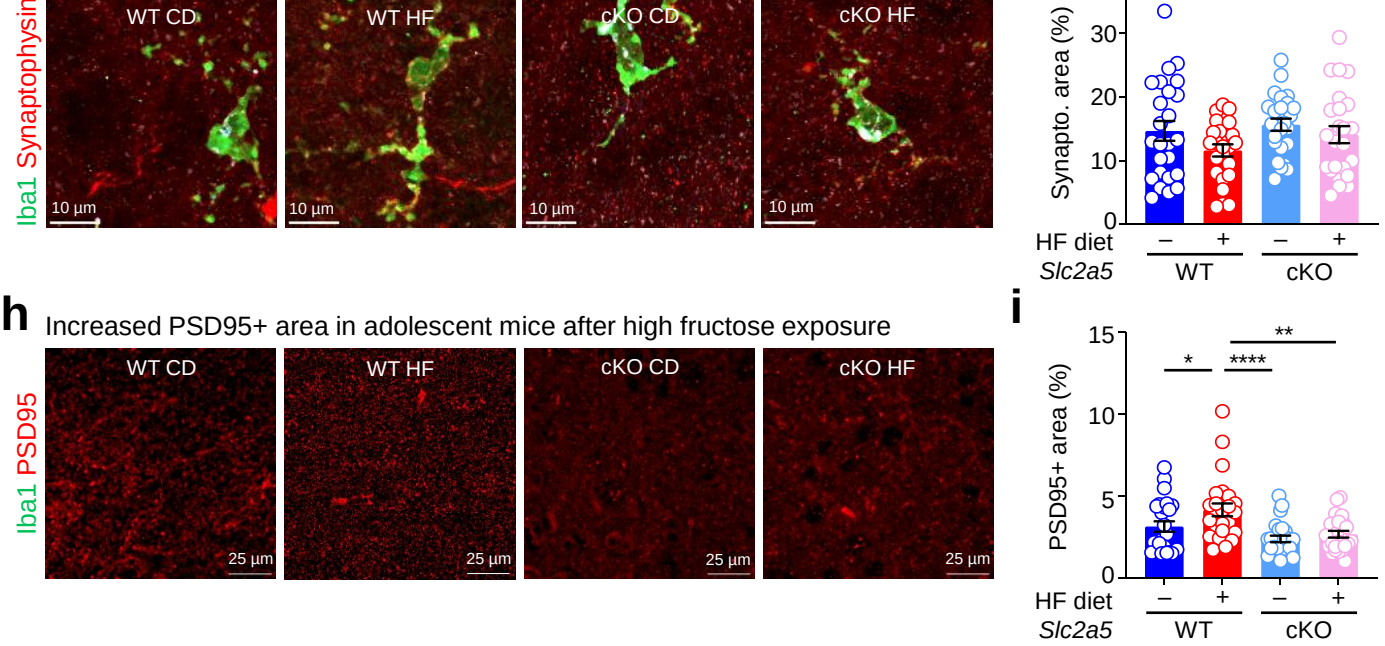

Supplement: Extended data file [file EMS206505-supplement-Extended_data_file.pdf]
