# Supplementary material for: Early life high fructose exposure disrupts microglia phagocytosis and impedes neurodevelopment
Source: Nature. Author manuscript; Available in PMC 2025 Aug 21. (PMC7617807; doi:10.1038/s41586-025-09098-5)
Supplement: Supplementary Information [file EMS206505-supplement-Supplementary_Information.pdf]

nature portfolio

https://doi.org/10.1038/s41586-025-09098-5

Supplementary information

Early life high fructose impairs microglial phagocytosis and neurodevelopment

In the format provided by the authors and unedited

Supplementary Source Data

Full uncropped and unprocessed scans for:

GLUT5
GAPDH

HK2
actin

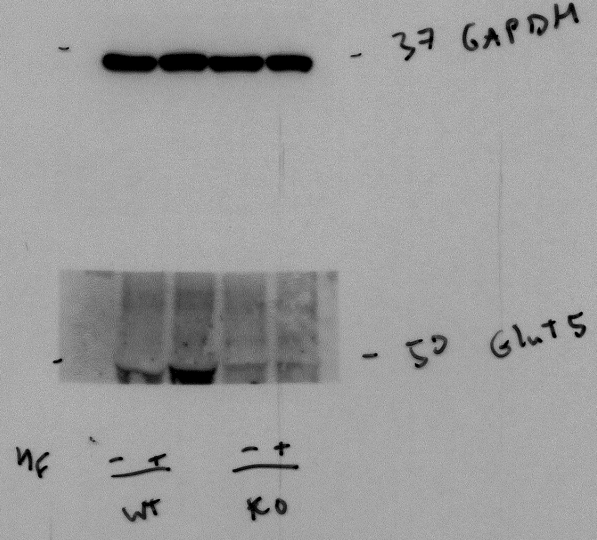
37 GAPDH
50 Glut5
- +
- +
WT
KO

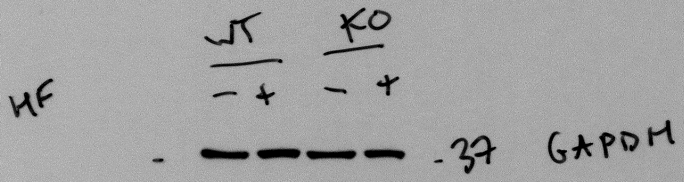
HF
WT
KO
− + − +
37
GAPDH

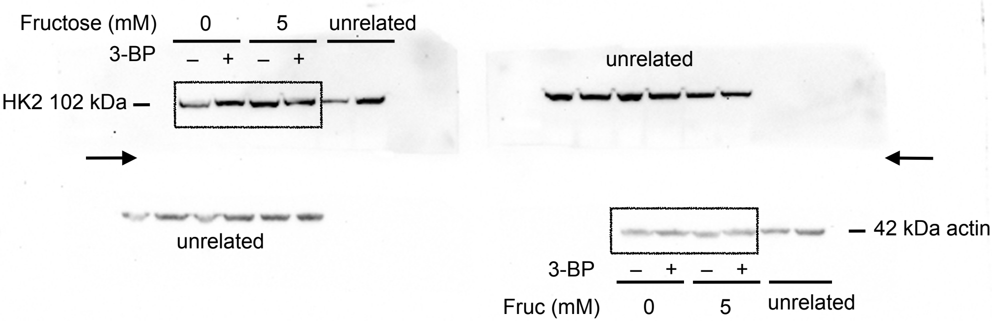
Fructose (mM)
0
5
unrelated
3-BP
–
+
–
+
HK2 102 kDa
unrelated
unrelated
42 kDa actin
3-BP
–
+
–
+
Fruc (mM)
0
5
unrelated
